# Rapid Dopaminergic Modulation of the Fish Hypothalamic Transcriptome and Proteome

**DOI:** 10.1371/journal.pone.0012338

**Published:** 2010-08-20

**Authors:** Jason T. Popesku, Christopher J. Martyniuk, Nancy D. Denslow, Vance L. Trudeau

**Affiliations:** 1 Centre for Advanced Research in Environmental Genomics, Department of Biology, University of Ottawa, Ottawa, Ontario, Canada; 2 Department of Physiological Sciences and Center for Environmental and Human Toxicology, University of Florida, Gainesville, Florida, United States of America; Ecole Normale Supérieure de Lyon, France

## Abstract

**Background:**

Dopamine (DA) is a major neurotransmitter playing an important role in the regulation of vertebrate reproduction. We developed a novel method for the comparison of transcriptomic and proteomic data obtained from *in vivo* experiments designed to study the neuroendocrine actions of DA.

**Methods and Findings:**

Female goldfish were injected (i.p.) with DA agonists (D1-specific; SKF 38393, or D2-specific; LY 171555) and sacrificed after 5 h. Serum LH levels were reduced by 57% and 75% by SKF 38393 and LY 171555, respectively, indicating that the treatments produced physiologically relevant responses *in vivo*. Bioinformatic strategies and a ray-finned fish database were established for microarray and iTRAQ proteomic analysis of the hypothalamus, revealing a total of 3088 mRNAs and 42 proteins as being differentially regulated by the treatments. Twenty one proteins and mRNAs corresponding to these proteins appeared on both lists. Many of the mRNAs and proteins affected by the treatments were grouped into the Gene Ontology categorizations of protein complex, signal transduction, response to stimulus, and regulation of cellular processes. There was a 57% and 14% directional agreement between the differentially-regulated mRNAs and proteins for SKF 38393 and LY 171555, respectively.

**Conclusions:**

The results demonstrate the applicability of advanced high-throughput genomic and proteomic analyses in an amendable well-studied teleost model species whose genome has yet to be sequenced. We demonstrate that DA rapidly regulates multiple hypothalamic pathways and processes that are also known to be involved in pathologies of the central nervous system.

## Introduction

Cellular regulation of the transcriptome and proteome is complex and the relationship between gene expression and protein changes *in vivo* remain poorly understood in fishes [1]. We used the adult female goldfish hypothalamus as a model system to characterize the rapid transcriptomic and proteomic responses to injection of dopamine (DA) receptor agonists. DA is widely distributed in the vertebrate brain and is involved in motivation, cognition, movement, and endocrine responses. DA exerts its effects via the D1- and D2-classes of 7-transmembrane domain G-protein-coupled receptors [2]. In fish, it is well understood that DA, acting through the D1 and D2 receptor, stimulates growth hormone release and inhibits luteinizing hormone (LH) release, respectively [3]. Upon ligand binding, the D1-receptor stimulates adenylate cyclase (AC) activity whereas the D2-receptor inhibits AC activity [4], leading us to hypothesize that the specific receptor agonists would lead to distinct transcriptomic and proteomic profiles in the hypothalamus that reflect the mode of action of the distinct receptors. Both D1 and D2 receptors also modulate intracellular calcium levels [2]. We chose to characterize the response to DA because it is a major central nervous system (CNS) neurotransmitter with a fundamental inhibitory role in vertebrate reproduction [3], [5] and because of the importance of DA to neurological disorders in humans [6], [7], [8].

Our model organism of choice was the goldfish, *Carassius auratus* [3] because i) the role of DA as a central regulator of reproductive processes is best-described in the goldfish; ii) a goldfish EST project has been initiated; iii) transcriptomic analysis is possible because of the development of a goldfish-carp cDNA microarray; iv) it is a member of the *Cyprinidae*, one of the largest vertebrate classes with over 2,400 species; and because v) goldfish are more amenable to physiological and endocrine manipulations than smaller fish such as zebrafish and medaka. On the other hand, the paucity of genomic and proteomic data in goldfish, and in many other important animal models other than laboratory rodents and humans, presents a major challenge to evolutionary and comparative physiologists.

To address this challenge, we developed a method for transcriptomic and proteomic comparison and demonstrate its utility for use on a model species with value to physiology and endocrinology but having limited genomic information. We provide insights into the hypothalamic processes that are under the regulation of DA in relation to its potent inhibitory actions on pituitary luteinizing hormone (LH) release and thus vertebrate reproductive function [3], [5].

## Materials and Methods

### Ethics Statement

All procedures used were approved by the University of Ottawa Protocol Review Committee (permit BL-234) and followed standard Canadian Council on Animal Care guidelines on the use of animals in research.

### Experimental animals and design

Common adult female goldfish were purchased from a commercial supplier (Aleong's International Inc., Mississauga, ON, Canada) and maintained at 18°C under a natural simulated photoperiod on standard flaked goldfish food. Goldfish were anaesthetized using 3-aminobenzoic acid ethylester (MS222) for all handling, injection, and dissection procedures. Sexually mature, pre-spawning (mid-May; GSI 4.5±1.3%) female goldfish (15–40 g) were injected intraperitoneally with either SKF 38393 (D_1_ agonist; SKF; 1-phenyl-2,3,4,5-tetrahydro-(1H)-3-benzazepine-7,8-diol; 40 µg/g) or LY 171555 (D_2_ agonist; LY; (-)-Quinpirole hydrochloride; 2 µg/g) purchased from Tocris (Ballwin, MO, USA). The experimental design and doses chosen were identical to Otto et *al.* [9] who showed rapid effects on goldfish brain somatostatin mRNAs. SKF was first dissolved in a minimal amount (0.099% final concentration) of dimethylsulfoxide (DMSO), and subsequently diluted with physiological fish saline (0.6% NaCl). Concentrations of DMSO up to 0.1% do not affect GH or LH levels [9]. LY was dissolved in saline. Control fish received 2 i.p. injections (5 µL/g body weight) of saline or the DMSO vehicle. SKF and LY-treated animals respectively received a second injection of either saline or DMSO to control for the 2 different drug vehicles.

After 5 hours, blood was sampled (400–600 µL) by puncture of the caudal vasculature via a 25-gauge needle attached to a 1-mL syringe. The fish were sacrificed by spinal transection and hypothalamic tissues were rapidly dissected and immediately frozen on dry ice. Hypothalami were pooled (3/tube) to increase RNA yield prior to RNA isolation. Serum was collected by centrifuging the blood at 4,000 g at 4°C for 10 minutes. Serum was stored at −80°C until used for the radioimmunoassay.

### Radioimmunoassay for Luteinizing Hormone

The double antibody RIA protocol of Peter *et al.* [10] was used to analyze serum LH levels, with minor modifications described by Zhao *et al*. [11]. Data were tested for normality using SPSS v17.0 and determined not to be normally distributed. Data were therefore log-transformed, determined to be normally distributed, and a one-way ANOVA was performed to test for significant differences (p<0.05).

### RNA isolation and quality and cDNA synthesis

RNA was isolated with the TRIzol method (Invitrogen, Burlington, ON, Canada) as per the manufacturer's protocol. Samples were treated with DNase on-column in an RNeasy Mini kit (Qiagen, Mississauga, ON, Canada). RNA quantity was evaluated using the NanoDrop ND-1000 spectrophotometer (Thermo Fisher Scientific). RNA quality was evaluated using the 2100 BioAnalyzer (Agilent); the RNA integrity number for all samples was >8.4.

### Microarray hybridizations

We previously described and validated the production and use of our goldfish-carp cDNA microarray [12], [13], [14]. The array contains 8832 cDNAs printed in duplicate and a detailed description is published elsewhere [15]. Four microarray hybridizations were performed for hypothalamic tissue for both D1 and D2 agonists (total of 8 arrays) to screen for the effects of the agonists in the neuroendocrine brain. Three separate pools of RNA from treated fish were hybridized to the microarrays, and a fourth hybridization was a replicate dye-reversal of one of the three RNA pooled samples. Hybridizations were carried out relative to a common pool of control samples (∼30 control fish) for each tissue, which decreases technical variation as only one reference is utilized while maintaining biological variation of the treatment samples [16]. All cDNA synthesis, labeling, and hybridizations were performed using the Genisphere 3DNA Array 900MPX kit according to the manufacturer's protocol (Genisphere, Hatfield, PA). Hybridizations and scanning protocols were described previously [12], [13], [14]. Briefly, microarrays were scanned at full-speed 10-µm resolution with the ScanArray 5000 XL system (Packard Biosciences/PerkinElmer, Woodbridge, ON, Canada) using both red and blue lasers. Images were obtained with ScanArray Express software using automatic calibration sensitivity varying photomultiplier (PMT) gain (PMT starting at 65% for Cy5 and 70% for Cy3) with fixed laser power at 80% and the target intensity set for 90%. Microarray images were analyzed with QuantArray (Packard Biosciences/Perkin Elmer), and raw signal intensity values were obtained for duplicate spots of genes. Raw intensity values for all microarray data and microarray platform information have been deposited in the NCBI Gene Expression Omnibus database (Series accession no. GSE14607 (SKF) and GSE14610 (LY)) under MIAME compliance. Generalized Procrustes Analysis [17] was used for normalization of the array data and the Significance Analysis of Microarrays (SAM) method [18] was used to identify significantly regulated transcripts.

### Protein quantification and database search using iTRAQ labeling

The iTRAQ labelling protocol has been previously described in detail in Martyniuk *et al.* [19]. Briefly, approximately 20 mg of hypothalamic tissue was collected and mechanically disrupted and homogenized in 500 µL RIPA (25 mM Tris-HCl pH 7.6, 150 mM NaCl, 1% nonyl phenoxylpolyethoxylethanol-40, 1% sodium deoxycholate and 0.1% SDS) (Pierce, Thermo Fisher Scientific Inc. Rockford, IL., USA) and proteins were precipitated in 3 mL of acetone. After removal of acetone, proteins were resuspended in iTRAQ dissolution buffer (Applied Biosystems Inc, Foster City, CA) and vortexed. Using 100 µg total protein/sample, we performed three independent iTRAQ labeling experiments following the manufacturer's protocol (Applied Biosystems Inc,). For proteomics analysis, each labeling reaction consisted of a single hypothalamus for control (label 114), LY 171555 (D_2_ agonist; label 115), and SKF 38393 (D_1_ agonist; label 117) (total n = 9 samples used; n = 3 per iTRAQ experiment). After labelling the independent samples for each iTRAQ experiment, they were mixed together and processed through desalting via a macrospin column Vydac Silica C18 (The Nest Group Inc, Southboro, MA), each and then subjected to off-line SCX fractionation on a polysulfoethylA column. The following fractions were collected for each of the three iTRAQ experiments: 7 (iTRAQ 1), 11 (iTRAQ 2), and 10 (iTRAQ 3). LC-MS/MS analysis on each of these fractions was performed on a hybrid quadrupole-TOF mass spectrometer QSTAR XL (Applied Biosystems).

Peptides were searched against a ray-finned fish database (details in [19] using MS/MS data interpretation algorithms within Protein Pilot™ (Paragon™ algorithm, v 2.0, Applied Biosystems). The Paragon algorithm searched iTRAQ 4-plex samples as variable modifications with methyl methanethiosulfonate as a fixed modification [20]. The Protein Pilot™ algorithm was selected to search automatically for biological modifications such as homocysteines. The confidence level for protein identification was set up to 1.3 (95%), which is the default setting for the detected protein threshold in a Paragon™ method. Proteomics System Performance Evaluation Pipeline (ProteomicS PEP, Applied Biosystems) in Protein Pilot™ was used to create a reversed ray-finned fish database to calculate a false discovery rate (FDR). When searching the ProteomicS PEP reverse database, 621 proteins were identified with an FDR of 1%, thus there is high confidence (>99%) in the peptide-protein assignments in this study. Differential expression ratios for proteins were obtained from Protein Pilot™ which calculates protein ratios using only ratios from the spectra that are distinct to each protein, excluding the shared peptides of protein isoforms. Peptides with low spectral counts were also excluded from the calculation of averages by setting the intensity threshold for the sum of the signal-to-noise ratio for all the peak pairs at >9. A protein with three high quality peptide spectra used in quantitation is considered to be a confident quantitation. However, we also report proteins in which two spectra were used in the quantitation for comparison. To calculate differential expression ratios, all identified spectra from a protein were used to obtain an average protein ratio relative to the control label (i.e. fold change). The p-value was calculated using the confidence intervals from the error factor generated in Protein Pilot™.

### Bioinformatics

Protein sequences from proteins identified by iTRAQ analysis as differentially expressed were downloaded from NCBI using extracted GI numbers with a BioPerl script (Fig. S1). The protein sequences were converted into a searchable database using formatdb. All of the nucleotide sequences identified as being differentially expressed (q<5%) from the agonist experiment were compared (blast-2.2.19) against the above database through Blast2GO [21]. A graphical depiction of the workflow is presented in Fig. S2.

## Results and Discussion

**Figure 1:**
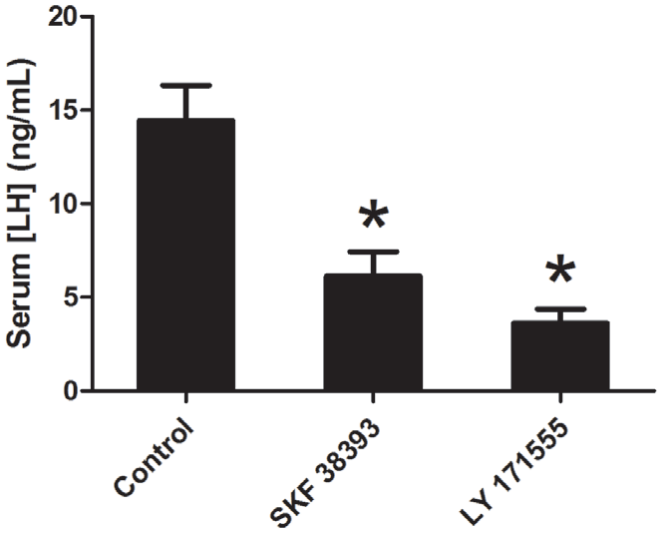
Serum LH concentration following DA agonist injections. Mean (± SEM) serum LH concentration in control and injected (40 ug/g SKF 38393 or 2 ug/g LY 171555) female goldfish (n = 23−26 each). Results presented are the average of 2 identical but independent experiments that showed similar results. Data was log-transformed to approximate normality and a one-way ANOVA was performed in SPSS v16 with significance considered at p<0.05, followed by Tukey's HSD multiple comparisons as data was homoscedastic. * signifies p<0.001 relative to control.

Our *in vivo* treatments both confirmed previous research and provided new hormone-regulatory data. Circulating serum LH was rapidly suppressed following DA agonist injections (Fig. 1). It is well known that DA, via the pituitary D2 receptor, is the primary inhibitor of LH release in goldfish and numerous other teleosts [5], [22], [23], [24], [25], [26], [27], [28], [29], [30], [31]. Here we corroborate these findings and show that LY 171555 (LY) rapidly reduced circulating LH levels to 25% of control. Unexpectantly, we found that the DA-D1 agonist SKF 38393 (SKF) decreased LH by 43%, which is a novel finding for DA regulation of *in vivo* LH release in fish. It is known that activation of D1-receptors inhibits the release of gonadotropin-releasing hormone (GnRH) [32], and thus may have an impact on GnRH-stimulated LH release. We have subsequently begun further investigation the involvement of D1 receptors in LH release [33]. Most relevant here, however, is that our DA agonist treatments produced physiologically relevant changes in circulating hormone levels, so we proceeded to analyse transcriptomic and proteomic responses in the hypothalamus, the central integrator of external and endogenous signals. Compared to other vertebrates, fish have very high hypothalamic levels of DA due to a duplicated tyrosine hydroxylase gene (*th2*) [34]. Importantly, the goldfish posterior tuberculum (TPp; or nucleus posterior tuberis; NPT), a region with intense immunostaining for *th1* but lacking immunostaining for dopamine β-hydroxylase, lies within the hypothalamus [35], [36], [37]. Thus, we proceeded to determine the effects of DA agonist injection on hypothalamic function.

### Transcripts identified in the goldfish hypothalamus as differentially regulated by dopamine agonists

In total, 3088 ESTs were identified as being statistically (q<5%) significantly differentially expressed following either D_1_- or D_2_-receptor stimulation in the hypothalamus. Many of these are as yet uncharacterized (Fig. S3). Of the 1042 ESTs that are annotated, gene ontology (GO) classifications (Fig. S4) revealed that a large percentage are involved in the regulation of biological process (13%), signal transduction (10%) and nucleotide binding (19%). Furthermore, 29% of the cDNAs were localized to GO Cellular Component category of the protein complex, suggesting that many of the proteins are involved in macromolecular complexes, reflecting the receptor targets for the agonists.

A recent review by Altar et *al*. [38] summarized targets for the identification of CNS diseases using transcriptional profiling of human post-mortem brain, animal models, and cell culture studies. Many of the transcriptional targets reported by Altar et *al.* [38] were also differentially regulated by DA in the goldfish hypothalamus. For example, mRNAs for glutamic acid decarboxylase (GAD) 1, microtubule-associated protein tau, serpin A, malate dehydrogenase, regulator of G-protein signaling, transferrin, s100 calcium binding protein, glutathione-S-transferase, calmodulin, α-amino-3-hydroxyl-5-methyl-4-isoxazole-propionate (AMPA) receptors, glial fibrillary acidic protein, N-methyl-D-aspartic acid (NMDA) receptor 1, glutamate transporter, calbindin, alpha enolase, peroxiredoxin, fructose-bisphosphate aldolase c, glutamine synthetase, and DA receptors and a DA transporter were all identified as being differentially regulated (Table S1) and are linked to CNS diseases such as Alzheimer's disease (AD), Parkinson's disease (PD), and schizophrenia, as well as brain aging [38], [39], [40].

Injection of SKF modulated hypothalamic mRNA levels for key transcripts in the glutamate and γ-aminobutyric acid (GABA) pathways (Table S1) in goldfish [3], [41]. In goldfish, GABA has a prominent stimulatory action on LH release by enhancing GnRH release and by reducing DA turnover in the hypothalamus [42], [43]. Our working hypothesis is that GABAergic systems transduce environmental (e.g. temperature) and endocrine (e.g. sex steroid) signals by rapid effects on both GnRH and DA to enhance LH release during seasonal gonadal redevelopment [41], [44], [45]. Results from the current study support this hypothesis.

### Protein identification in the goldfish hypothalamus

For this experiment, we repeated DA agonist treatments on the same date the following year using an identical design. Serum LH was similarly decreased in both years and the data presented were combined (Fig. 1). The hypothalami from this experiment were subjected to iTRAQ proteomic analysis.

There were 621 proteins identified in this study using a ray-finned fish database previously constructed [19] (Table S2). The total number of peptide spectra detected was 8569, representing 4779 distinct peptides that are listed in Dataset S1. Of the peptides identified, 59.7% could be assigned to a protein, leaving approximately 40% of the spectra unidentified by homology searches against other ray-finned fishes.

**Figure 2:**
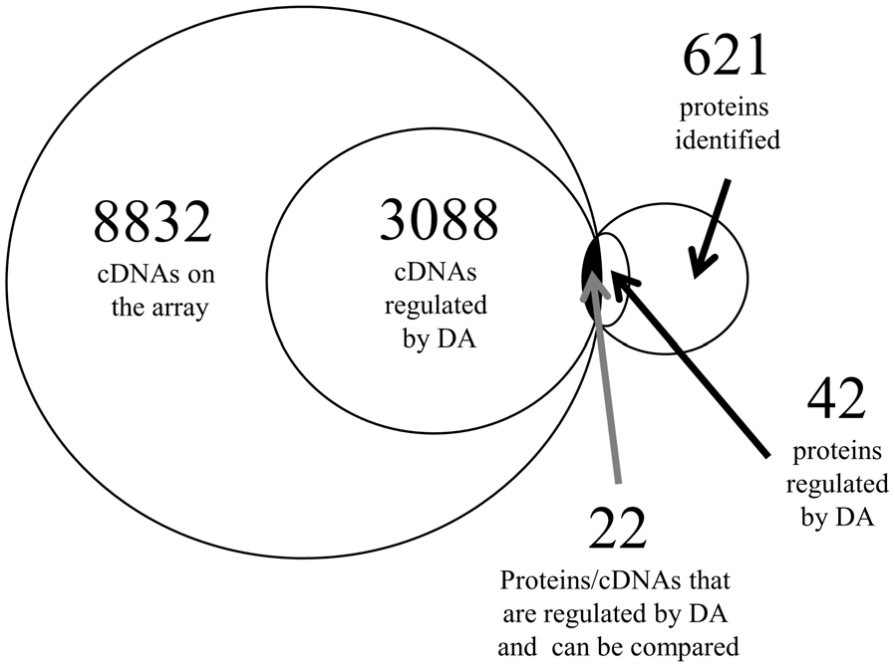
Venn Diagram summarizing the number of cDNAs and proteins found in the hypothalamus of female goldfish. The comparison of those regulated by DA was limited to cDNAs (q<5%) and proteins (FDR-adj p<0.05) identified as being statistically significant. Duplicate cDNAs were removed; cDNAs and proteins were counted once regardless if they were regulated by both agonists. The complete listing of cDNAs and proteins are listed in Tables S1 and S2, respectively.

**Figure 3:**
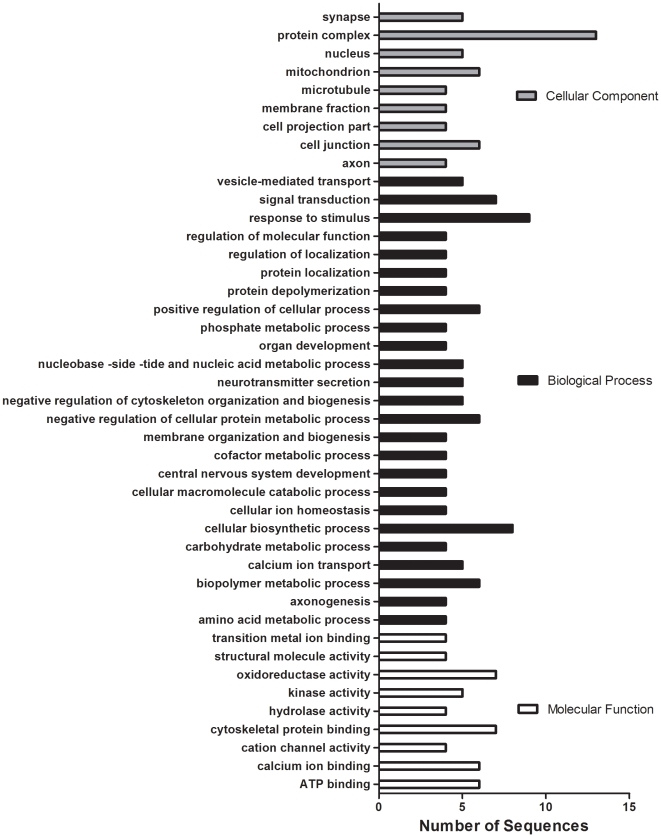
GO categorization of differentially expressed proteins identified in the current study. Proteins identified by iTRAQ (42; p<0.05) were binned into multilevel GO categorizations with a sequence cut-off of 3. Both treatments (D1 and D2) and both directions are included in this analysis but are counted only once if the protein is common to both treatments.

Of the 621 identifiable proteins, 42 were determined as being significantly (*p*<0.05) differentially regulated by either SKF or LY (Fig. 2). The protein dataset (for both D1 and D2 results combined) was analyzed using Blast2GO and binned into their corresponding GO terms (Fig. 3). Similarly to the mRNAs affected in this study, many of the proteins affected by the treatments are localized to the GO Cellular Component of the protein complex and are involved in a wide variety of biological processes, including signal transduction, response to stimulus, and both positive and negative regulation of cellular process. Of interest here are the proteins in the GO category of Biological Processes as related to neurotransmitter secretion and calcium ion transport. Calcium/calmodulin-dependent kinase II α subunit (CaMKIIα), calbindin 2, neuronal calcium-binding protein 2, plasma membrane calcium ATPase 4, and calmodulin (CaM) proteins were significantly affected by at least 1 of the DA agonists (Table 1).

**Table 1:** Proteins and mRNAs identified and affected by DA agonists in the hypothalamus of goldfish.

Protein				Corresponding mRNA			
Accession	Name	Fold Change		Accession	Blast2GO-annotated mRNA (NCBI)	Fold Change	
		SKF	LY			SKF	LY
AAW82445	14 kDa apolipoprotein*	−1.3		CA967592	14 kda apolipoprotein	−1.6	
AAW82445	14 kDa apolipoprotein*		−1.5	CF662502	14 kda apolipoprotein		1.9
CAG00145	25 kDa synaptosomal-associated protein		−1.5	CA969142	synaptosomal-associated protein 25		1.4
NP_956213	adaptor-related protein complex 2, beta 1 subunit	1.3					
AAH83251	Atp2b4 protein		1.4				
AAZ38450	beta thymosin-like protein		−1.3				
AAF79948	brain-type fatty-acid binding protein; B-Fabp		−1.4				
CAK04737	calbindin 2, like	−1.2					
NP_001017741	calcium/calmodulin-dependent protein kinase II alpha	2.3	1.8				
Q71UH6	Calmodulin		−1.2	CA969795	calmodulin variant 1		1.4
CAF92971	Creatine kinase, brain		−1.4				
NP_942096	creatine kinase, mitochondrial 1		1.2				
CAK10905	cytochrome c oxidase subunit IV isoform 1	1.2					
AAV52802	glutamine synthetase	1.4	−1.3	FG393017	glutamine synthetase	1.9	
ABD67511	glutathione S-transferase rho	−1.3	−1.2	CA964231	glutathione S-transferase rho	−1.6	
AAV52803	glyceraldehyde-3-phosphate dehydrogenase	−1.4		DY231775	GAPDH	1.6	
AAA21578	kainate receptor α subunit	1.8	1.3	FG392717	kainate receptor α subunit	1.4	
AAH63955	Krt5 protein		2.6				
AAM21708	liver-basic fatty acid binding protein*		3.6	CA968596	fatty acid binding protein liver basic		1.4
AAM21708	liver-basic fatty acid binding protein*	8.6		CA970443	fatty acid binding protein liver basic	−1.5	
NP_956241	malate dehydrogenase 1a, NAD (soluble)	1.2		CA964750	malate dehydrogenase nad	2.0	1.3
ABC69306	myoglobin isoform 2	−1.4		CA968088	myoglobin		1.4
NP_958898	N-ethylmaleimide-sensitive factor		1.3	FG392958	n-ethylmaleimide-sensitive factor	1.4	
CAN88379	novel protein sim to vert EF hand calcium binding protein 2 (EFCBP2)		−1.1				
CAK05381	parvalbumin		−1.1	CA969705	parvalbumin		1.5
ABF57553	Pi-class glutathione S-transferase	−1.7	−1.6				
BAA78376	polypeptide elongation factor 1 alpha	1.3		CA967511	eukaryotic translation elongation factor 1 alpha 1	−1.8	
XP_001340376	PREDICTED: myelin basic protein isoform 3	−3.9		CA967910	myelin basic protein	2.0	
CAF98839	PREDICTED: similar to germinal histone H4 gene		−1.1				
XP_001338014	PREDICTED: similar to microtubule-associated protein 1 A		1.4				
XP_696230	PREDICTED: similar to microtubule-associated protein tau	−1.1		CA967834	microtubule-associated protein tau	1.9	
XP_001335551	PREDICTED: similar to Myelin basic protein	−1.5	−1.5				
XP_691535	PREDICTED: similar to Nj-synaphin 2		−2.2				
CAF95822	Putative histone cluster 1, H2bb	−1.6	−1.5				
AAG14350	putative oncoprotein nm23	−1.3		CA964203	non-metastatic cells 2, protein (NM23B)	−1.6	
AAI14255	short chain dehydrogenase/reductase	−1.9	−1.5	CA968680	dehydrogenase reductase sdr family member 12		1.3
NP_001091958	spectrin alpha 2	1.3		FG392760	spectrin alpha 2	1.4	
NP_001017850	stathmin 1/oncoprotein 18*		−1.3	CA969799	stathmin 1 oncoprotein 18 variant 8		1.4
NP_001017850	stathmin 1/oncoprotein 18*	−1.5		CA966170	stathmin 1 oncoprotein 18 variant 8	1.5	
NP_001018488	synuclein, gamma b (breast cancer-specific protein 1)	−1.5					
AAM90972	transferrin variant A1	1.5		CA968595	transferrin variant c	1.9	
AAM90973	transferrin variant B1		−1.9				
NP_705954	triosephosphate isomerase 1b	−1.3	−1.2	CA968504	triosephosphate isomerase 1b	1.8	
NP_997770	tyrosine 3-/tryptophan 5-monooxygenase activation protein, epsilon polypeptide	1.1					
AAQ94569	ubiquitin C	−1.3					

Proteins were determined by iTRAQ as being significantly (FDR-adj p<0.05) differentially regulated in the hypothalamus of female goldfish treated with either SKF 38393 (SKF) or LY 171555 (LY) agonists. This table also show the cDNAs corresponding to the proteins identified by microarray analysis as significantly (q<5%) affected by the same treatments. Negative values indicate a decrease relative to control. Absent values indicate either that no significant change was detected, or, in the case of the mRNAs, that the corresponding cDNA was not present on the array. mRNAs were annotated using Blast2GO's Blast Descriptor Annotator with default values except the Blast ExpectValue was changed from 1.0E-3 to 1.0E-5. Following the Mapping step, the Annotation Configuration E-Value-Hit-Filter was changed from 1.0E-6 (default) to 1.0E-8 to increase the likelihood of proper GO annotation. Duplicates were assessed on the basis of sequence comparison and removed if a similar expression was observed. In the case where different expression profiles were seen (*), both ESTs were included, as it is possible that the sequences correspond to separate genes.

Calmodulin protein was decreased by LY, but not SKF, suggesting that in hypothalamic CaM expression is D2-, rather than D1-, receptor-regulated. Previous research demonstrated that CaM is expressed in the hypothalamus and the pituitary of goldfish and LY, but not SKF, decreased CaM mRNA levels in goldfish pituitary cells [46]. Together the data indicate CaM is under the regulation of the D2 receptor in the goldfish hypothalamo-pituitary system. We have also identified CaM as being important and regulated in the hypothalamus using a meta-type analysis of data from multiple goldfish microarray experiments performed across the seasonal breeding cycle. The mRNA for CaM was relatively highly expressed in the hypothalamus of sexually mature females in May, compared to both sexually regressed (August) or recrudescing animals in the gonadal redevelopment phase (December) [47]. This information, coupled with the changes in mRNA and protein levels of CaM (this study), suggests that CaM may be important for DA inhibition on LH release and thus inhibitory control of reproduction.

CaMKIIα protein levels, whose transcript levels follow the same seasonal profile as CaM (high in May, low in August and December) [47], were increased in both D1- and D2-agonist treated fish suggesting that, as for CaM, CaMKIIα may be important in hypothalamic signalling. CaMKII phosphorylates cAMP response binding element (CREB) protein, thereby inhibiting its function [48], which may lead to downstream transcriptional repression of genes involved in reproduction. Furthermore, CaMKII positively regulates the D2 receptor promoter in rats [49], suggestive of a feedback mechanism of DAergic action.

We observed a decrease in hypothalamic Apo-14 protein expression with both D1- and D2- receptor agonists. Apo-14 appears to be specific to teleost fish [50], although a recent phylogenetic analysis revealed that Apo-14 is the homologue to mammalian ApoA-II [51]. Apo-14 is mainly expressed in liver and brain of adult orange-spotted groupers and has been suggested to play a role in neuronal growth and repair [52], similar to ApoE [53]. Vitale and Carbajal [54] demonstrated that DA induces substantial cytoskeletal remodelling in rat lactotrophs *in vitro*. The decreases in Apo-14, stathmin 1, microtubule-associated protein tau, along with an increase in microtubule-associated protein 1A and spectrin alpha 2 (Table 1) suggests that DA may also have remodelling effects on the cytoskeleton of cells in the goldfish hypothalamus. The likely high energetic demands for such remodelling is supported by the observed increase of mitochondrial creatine kinase, cytochrome c oxidase subunit IV, and malate dehydrogenase protein levels (Table 1).

### A comparison of the differentially expressed transcriptome to the differentially expressed proteome in response to DA agonists

The protein dataset was further compared to the microarray dataset by extracting the GI numbers from the protein results. A BioPerl script (Fig. S1) was used to obtain the corresponding amino acid sequences from GenBank, which were converted into a database that can be queried using the BLAST algorithm. This step was necessary in order to obtain the longest possible protein sequence data for the comparison. The nucleotide sequences represented on the microarray were compared (BLASTx) to this differentially-expressed protein database. The results (Table 1) show directional correlation for some mRNAs and proteins (for example, kainate receptor α subunit and 14 kDa apolipoprotein for D1 and liver-basic fatty acid binding protein for D2), while others are inversely correlated (for example, stathmin 1/oncoprotein 18 with either agonist). The mRNAs and their respective proteins exhibiting discordant directional change following agonist treatments nevertheless indicate that particular pathways and processes are DA-regulated. Differences in the direction of change between transcript and protein is likely related to our single sampling time-point, as the time-series relationship between changes in transcript versus protein *in vivo* are poorly understood in fish [1]. Furthermore, the regulatory mechanisms of the genome and proteome are complex and both turnover and stability of mRNA levels are important for translation of mRNA into protein [55]. For example, if the mRNA is decreased, but the protein is increased, it is possible that the mRNA has already begun to be degraded. Conversely, if the mRNA is increased, but the protein is decreased, there may be regulation of translational pathways, or increased protein degradation leading to induced transcription. These are good candidates for temporal (i.e., 1–3 hr time-course), biochemical (with/without cycloheximide) and pulse-chase analysis to better understand the differences. The interest here, however, lies with those mRNAs and proteins that share a common direction. In the D1-agonist-treated fish, 8 out of the 14 common mRNAs/proteins (57%) share a common directional change, whereas only 1 out of 7 of the common mRNAs/proteins (14%) for the D2-agonist-treated fish that change in parallel (Table 1). These results are comparable to what has been shown in the rat colon mucosa *in vivo* where only 16% direction identity between the transcriptome and the proteome was found [56]. Furthermore, that study included the development of a TRIzol®-based method to analyze both the transcriptome and the proteome from the same sample, which should reduce disagreements in the gene-protein correlation. Our results show that a comparable directional correlation from independent animals and experiments can also be achieved. This is significant, as it shows that the technique is applicable to a species with limited genomic information.

While some of the proteins identified as differentially expressed had corresponding changes in mRNA levels, many other cDNAs representing coding sequences for other proteins that were regulated were not printed on our array. For example, the Nj-synaphin 1 (also known as complexin 1) protein was identified as being down-regulated 2.2-fold in response to the D2-agonist (Table 1) but the complexin cDNA was not on the array.

Interestingly, in addition to complexin 1, proteins for both N-ethylmaleimide-sensitive factor (NSF) and soluble NSF attachment protein- (SNAP-) 25, all of which are major players in the exocytosis of neurosecretory vesicles [57], were affected by the D2 agonist. Current evidence [58], [59] indicates that complexin holds the vesicle in a “ready-to-release” state near the membrane, while preventing the spontaneous zippering of the t- and v-SNAREs and thus spontaneous fusion of the vesicle to the membrane. Upon introduction of Ca^2+^, which binds to synaptotagmin, complexin is removed and the membranes fuse, resulting in exocytosis. Since complexin expression is decreased, our results suggest that DA is stimulating some aspects of exocytosis in the hypothalamus via the D2 receptor. However, we found that SNAP-25 protein levels were reduced by the D2-agonist, suggesting that DA may also be inhibiting some of the exocytotic machinery. Perhaps this is a homeostatic mechanism to prevent or reduce the release of neurotransmitters and neurohormones in response to acute DAergic overstimulation.

NSF protein levels were increased in response to the D2 agonist. NSF transcript levels were initially found to be decreased in schizophrenic patients [60] but this was not observed in subsequent studies [38]. Similarly to CaM, the meta-analysis by Zhang et *al.* [47] identified NSF transcripts as being relatively highly expressed in May when goldfish are sexually mature. This information, coupled with the D1-mediated increase in NSF mRNA or D2-mediated increase in NSF protein found in this study, suggest that the DAergic inhibition of LH release involves hypothalamic NSF-dependent mechanisms.

Glutamine synthetase (GlnS) mRNA and protein levels were increased in response to SKF (Table 1). GlnS converts glutamate (Glu) to glutamine (Gln) and thus may limit the available pool of Glu, which is an excitatory neurotransmitter stimulating LH release in vertebrates including goldfish [41]. Increased GlnS could also potentially limit the Glu available to be converted by GAD to GABA. The observation in this study that GlnS is increased in response to a D1-specific agonist supports this hypothesis and suggests a possible mechanism of decreased LH secretion via D1-receptor stimulation. In contrast to SKF, GlnS protein levels were decreased in response to LY, with no observable effect on GlnS mRNA levels (Table 1). This differential response to the 2 DA agonists is likely due to responses in adenylate cyclase (AC) [61] since both receptors act via this second messenger system [2]. Generally, D1-class receptors, through interactions with G_s_ proteins, stimulate AC, whereas D2-class receptors, through interactions with G_i_ proteins, inhibit AC [2]. Glutamate can also be converted to glutathione-conjugated products through multiple enzymatic steps with the final step being mediated by glutathione S-transferase (GST). GST rho and Pi-class GST (GSTp) protein levels were reduced by both DA receptor agonists in the current study. It is not clear at this time whether the reduced GST protein levels are the result of DA receptor stimulation or rather a consequence of reduced substrate flux through that pathway initiated by limited pool of available Glu, as discussed above. However, it is likely not the latter case, as GST protein levels were reduced by both DA agonists, but GlnS protein levels were affected in different directions.

Glutathione is an antioxidant and helps to protect cells against damage from reactive oxygen species [62]. DA has been shown to induce apoptotic cell death in a CNS-derived catecholaminergic cell line [63] and Ishisaki et *al*. [64] identified GSTp as a candidate that protects against cell death in PC12 cells. Furthermore, inhibition of GSTp increased DAergic neuronal cell death in Swiss-Webster rats treated with MPTP [65], a specific DAergic neurotoxin. Interestingly, Shi et *al.* [66] recently reported increased GSTp protein levels in synaptosomal fractions from the frontal cortices of patients with pathologically-verified PD and suggest that GSTp may be important in the progression of the disease. In studies with GSTp-null mice, Henderson et *al.* [67] demonstrated that GSTp may enhance the hepatotoxicity of acetaminophen. While speculative, the observation of decreased GSTp protein levels in response to either D1- or D2-specific agonists in the current study suggests that DA may also modulate hypothalamic neuronal cell death in fish. This hypothesis is further supported by predominant increases observed in mRNAs for multiple heat shock proteins, ubiquitination enzymes, and proteasomal subunits by both D1- and D2-specific agonists (Table S1).

Several other proteins identified as differentially regulated by DA in goldfish have also been reported to be involved in human neurological disorders. For example, malate dehydrogenase, CaM, transferrin, tyrosine-3-monooxygenase/tryptophan-3-monooxygenase activation protein epsilon (YWHAE), microtubule-associated protein tau and beta-synuclein are among proteins identified that are known to be involved in neurodegenerative and/or psychiatric diseases [38].

There is a discrepancy between the number of mRNAs and proteins that were identified as differentially regulated that must be addressed. Many mRNAs for abundant ribosomal proteins were induced, and some of the corresponding proteins were detected, but did not change. This is not unexpected since it would be difficult to observe a change in protein concentration above the background of these proteins found in ribosomes in eukaryotic tissues. This is similar to what has been demonstrated in yeast [68]. However, for other mRNAs/proteins, there may be other factors restricting concordant changes. For example, our use of a “snapshot” time frame study is a likely limitation that does not allow us to take into account differences in mRNA versus protein half-lives. Furthermore, steady-state mRNA levels for many ESTs were increased by 5 h following agonist treatments, but the translational machinery may require additional time to efficiently produce the corresponding proteins. Importantly, despite the recognized limitations we outline, the level of concordance in the mRNA and protein changes in the goldfish brain are well within the ranges seen with better characterized vertebrate systems [56], [69], [70]. Future studies aimed at examining the temporal correlation between the hypothalamic transcriptome and proteome should reveal further relationships and critical pathways regulated by the neurotransmitter DA, and provide insights into the neural processes governing reproduction.

In conclusion, we have demonstrated the applicability of advanced high-through genomic and proteomic analyses in an amenable well-studied teleost model species whose genome has yet to be sequenced. Furthermore, we demonstrate the first evidence of D1-receptor involvement in the inhibition of LH release and suggest a mechanism through the potential modulation of other stimulatory neurotransmitters, namely glutamate and/or GABA. Refinement of the bioinformatic methods for time-course analysis should further reveal the importance of DA in regulating hypothalamic function.

## Supporting Information

Figure S1 Perl script used to extract amino sequences from GenBank.
(0.24 MB TIF)

Figure S2 Information workflow diagram for comparing mRNAs to proteins.
(0.42 MB TIF)

Figure S3 Number of ESTs identified by microarray analysis as being statistically (q<5%) differentially regulated by dopamine agonists in the hypothalamus of female goldfish 5 h post-i.p.-injection. The data distribution is shown as output from Blast2GO. Duplicates were removed. Overlapping ESTs (i.e. ESTs regulated by more than 1 agonist) are indicated as “Shared between…”.
(0.44 MB TIF)

Figure S4 Multilevel Gene Ontology categorization of the 1042 annotated ESTs into a) Biological Process, b) Molecular Function, and c) Cellular Component. Annotations were first converted to GO-Slim annotations (goslim_generic.obo) and the multilevel chart was constructed using a sequence convergence cutoff of 30 to reduce the complexity of the chart. Both agonists and both up- and down-regulated genes (q<5%) are included in this analysis.
(3.21 MB TIF)

Table S1 Complete list of cDNAs identified as significantly (q<5%) differentially regulated by SKF 38393 (SKF) or LY 171555 (LY). Negative fold changes indicate a decrease in the mRNA level.
(1.07 MB DOC)

Table S2 All goldfish proteins identified in the hypothalamus in this study. Proteins in which a single peptide was used in identification are also presented in this table. % Cov is the amount of amino acid coverage (%) by peptides. Ratios (e.g. 115∶114) are each treatment (tag 115 or 117) divided by control (tag 114) to obtain relative fold change. Pval is the p-value after all peptides for a protein were used for quantitation. The Error Factor (EF) expresses the 95% uncertainty range for a reported ratio. The true protein ratio is expected to be found between the (reported ratio)*(EF) and the (reported ratio)/(EF) 95% of the time. Peptides used in quantification also included all peptides with post-translational modifications and all charge states (Dataset S1). Peptides that do not have a Ratio or P-value were not quantified because 1) peptide signal was too low; 2) peptide did not meet standard for quantitation; or 3) peptide belonged to more than one unique protein.
(0.75 MB DOC)

Dataset S1 The total number of peptide spectra detected in the goldfish hypothalamus
(2.92 MB XLS)
