# Supplementary figures and images for: Rapid Dopaminergic Modulation of the Fish Hypothalamic Transcriptome and Proteome
Source: PLoS One. 2010 Aug 20;5(8):e12338. doi: 10.1371/journal.pone.0012338 (PMC2924890; doi:10.1371/journal.pone.0012338)

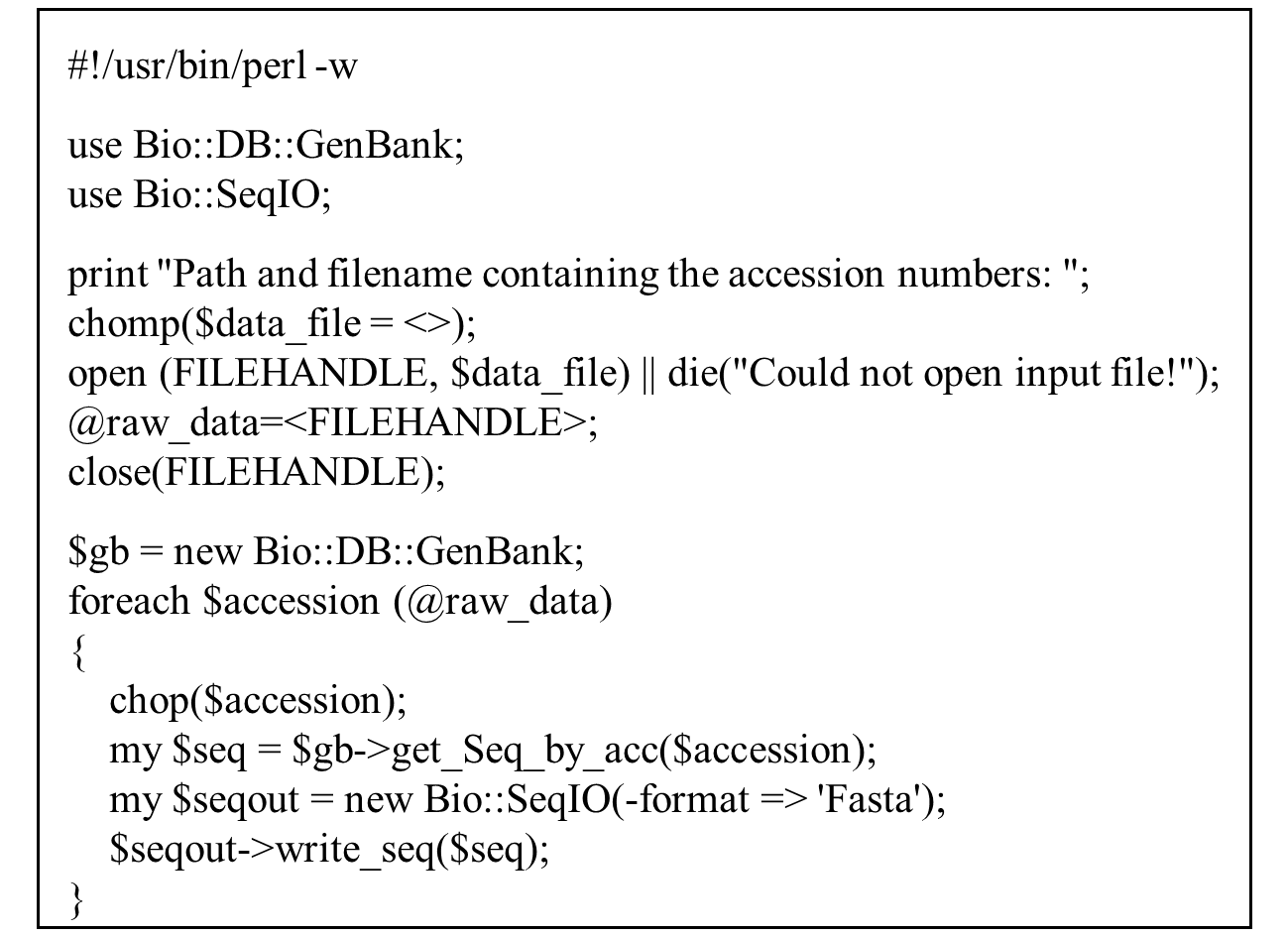

Supplement: Figure S1 — Perl script used to extract amino sequences from GenBank. (0.24 MB TIF) [file pone.0012338.s001.tif]

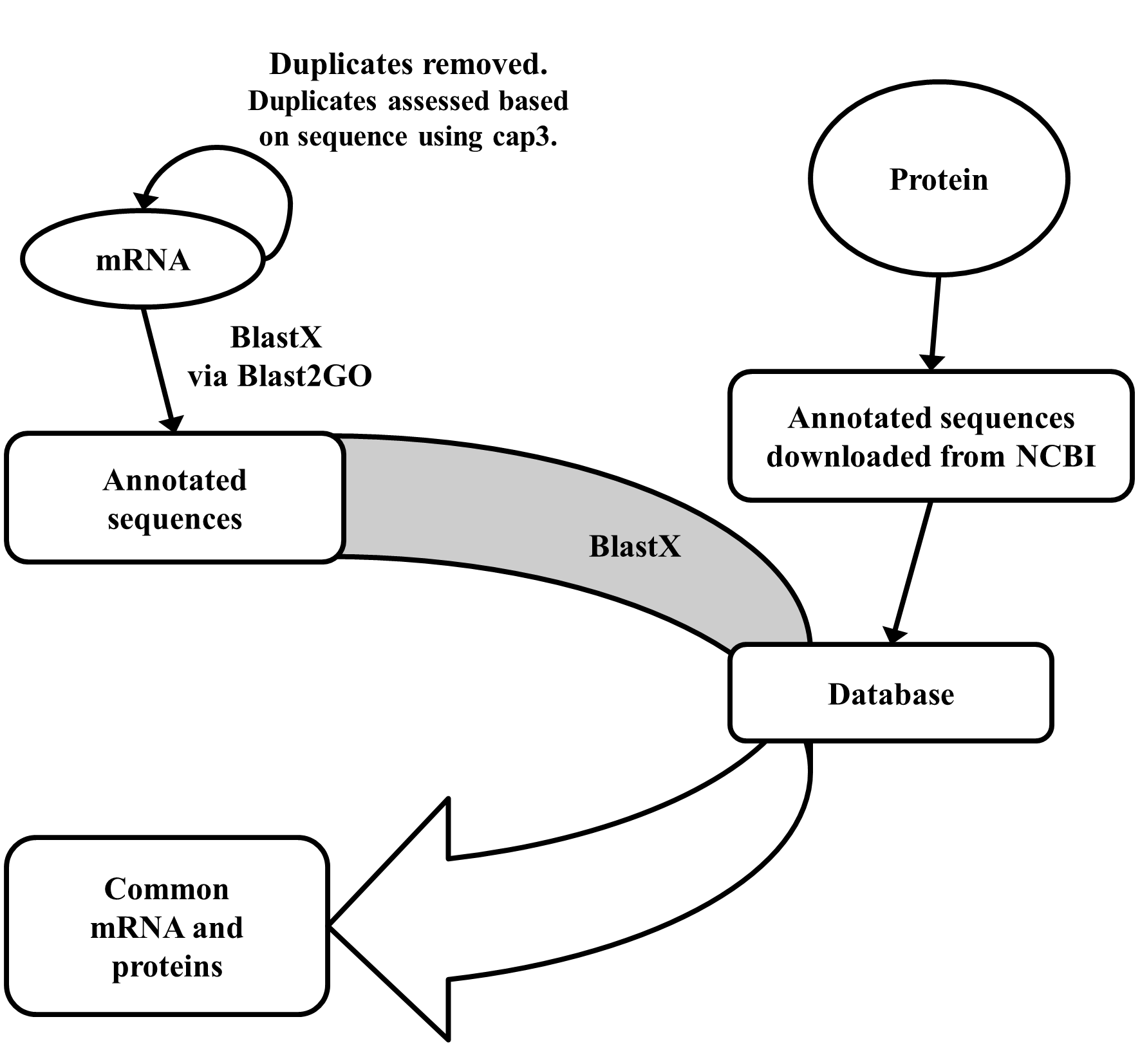

Supplement: Figure S2 — Information workflow diagram for comparing mRNAs to proteins. (0.42 MB TIF) [file pone.0012338.s002.tif]

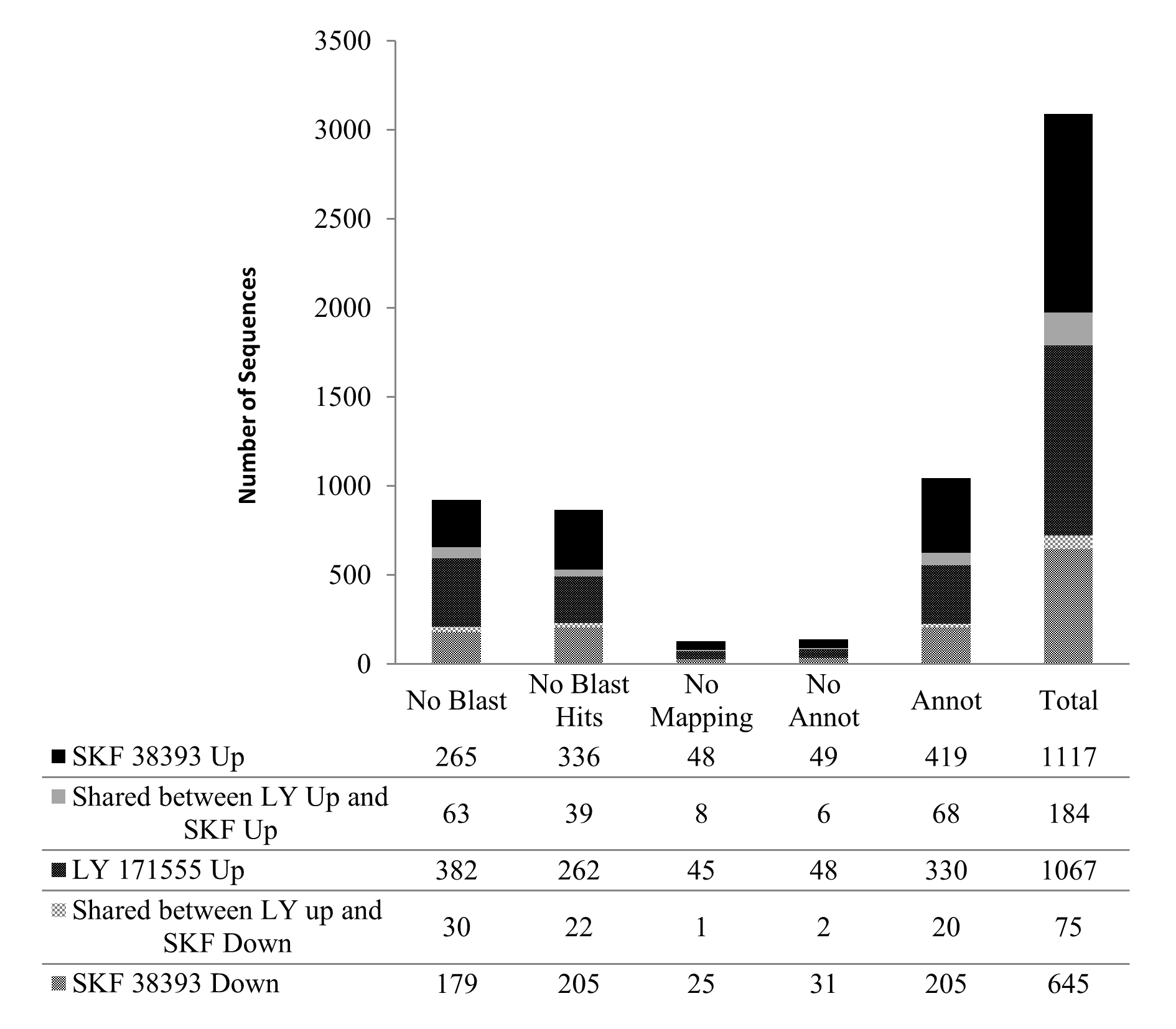

Supplement: Figure S3 — Number of ESTs identified by microarray analysis as being statistically (q<5%) differentially regulated by dopamine agonists in the hypothalamus of female goldfish 5 h post-i.p.-injection. The data distribution is shown as output from Blast2GO. Duplicates were removed. Overlapping ESTs (i.e. ESTs regulated by more than 1 agonist) are indicated as “Shared between…”. (0.44 MB TIF) [file pone.0012338.s003.tif]

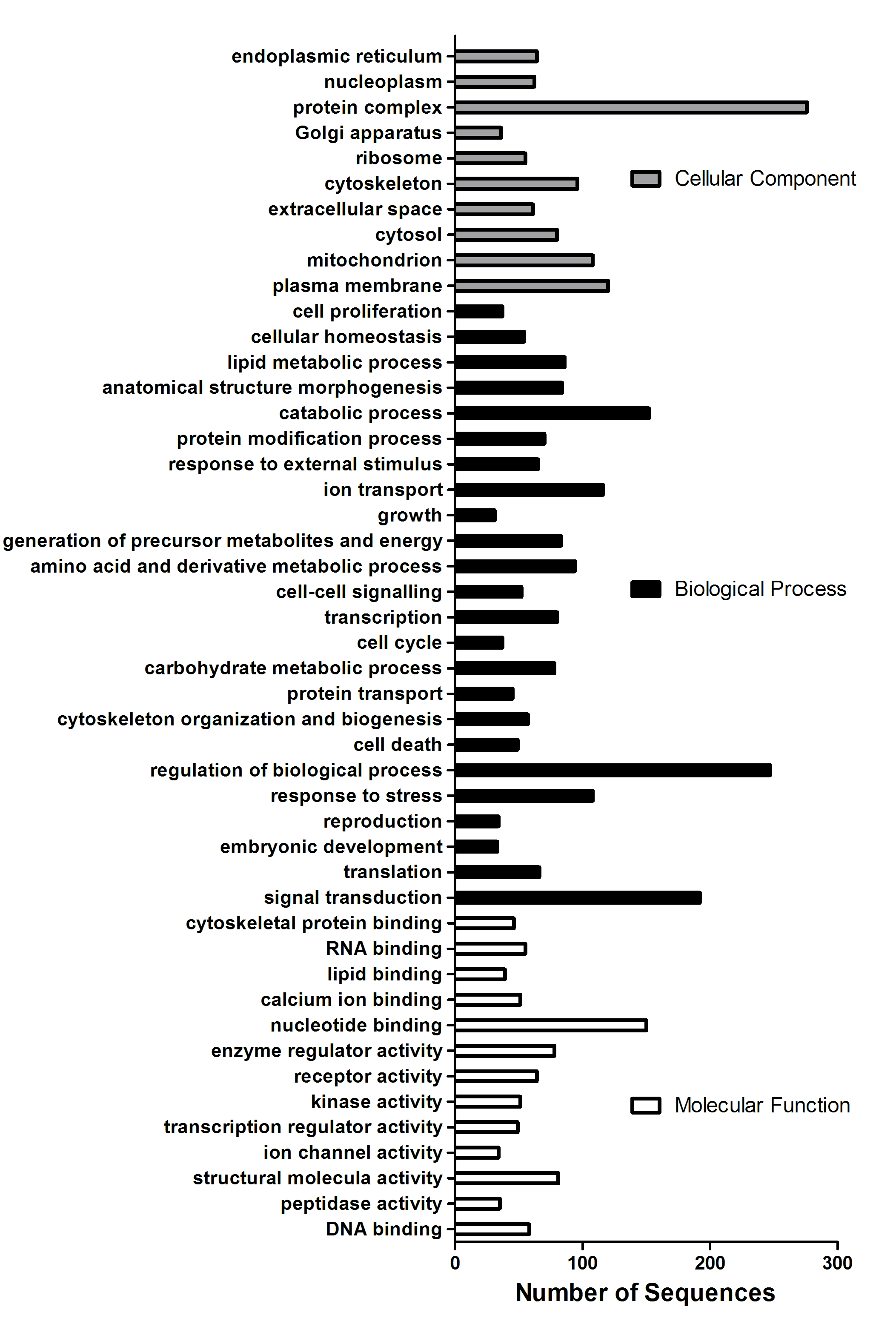

Supplement: Figure S4 — Multilevel Gene Ontology categorization of the 1042 annotated ESTs into a) Biological Process, b) Molecular Function, and c) Cellular Component. Annotations were first converted to GO-Slim annotations (goslim_generic.obo) and the multilevel chart was constructed using a sequence convergence cutoff of 30 to reduce the complexity of the chart. Both agonists and both up- and down-regulated genes (q<5%) are included in this analysis. (3.21 MB TIF) [file pone.0012338.s004.tif]
