# Supplementary material for: Rapid Dopaminergic Modulation of the Fish Hypothalamic Transcriptome and Proteome
Source: PLoS One. 2010 Aug 20;5(8):e12338. doi: 10.1371/journal.pone.0012338 (PMC2924890; doi:10.1371/journal.pone.0012338)
Supplement: Table S1 — Complete list of cDNAs identified as significantly (q<5%) differentially regulated by SKF 38393 (SKF) or LY 171555 (LY). Negative fold changes indicate a decrease in the mRNA level. (1.07 MB DOC) [file pone.0012338.s005.doc]

| **AURATUS GeneID** | **dbEST Accession** | **Blast2GO-annotated EST** | **Agonist** | **Fold Change** |
| --- | --- | --- | --- | --- |
| 09a17 | CA964109 | 11-beta-hydroxysteroid dehydrogenase-like protein | SKF | 1.6 |
| 06i22 | CF662502 | 14 kda apolipoprotein | LY | 1.9 |
| 08p07 | CA968826 | 14 kda apolipoprotein | SKF | 2.6 |
| 05o22 | CA967592 | 14 kda apolipoprotein | SKF | -1.6 |
| 11f14 | CA966092 | 24-dehydrocholesterol reductase | SKF | 1.8 |
| 06h10 | CA965430 | 2-aminoethanethioldioxygenase | LY | 1.4 |
| 24b12 | CA969740 | 3-hydroxy-3-methylglutaryl-coenzyme a reductase | SKF | -1.9 |
| 07b14 | CA968457 | 3-hydroxyanthranilate-dioxygenase | SKF | 1.6 |
| 19k03 | CA966211 | 3-hydroxybutyrate dehydrogenase type 2 | SKF | 2.1 |
| 08f14 | CA968676 | 40s ribosomal protein s27 | LY | 1.3 |
| 08a02 | CA968588 | 60s acidic ribosomal protein p1 | SKF | 1.8 |
| 08a02 | CA968588 | 60s acidic ribosomal protein p1 | LY | 1.5 |
| 10m03 | CA970457 | 60s acidic ribosomal protein p1 | LY | 1.3 |
| 13k14 | CF662709 | 60s ribosomal protein l5 | SKF | 1.4 |
| 08f09 | CA968671 | 60s ribosomal protein l5 | LY | 1.3 |
| 12h08 | CA967022 | 6-phosphofructo-2-kinase fructose--biphosphatase 1 | SKF | 2 |
| 19h15 | CA969115 | 6-phosphofructo-2-kinase fructose--biphosphatase 4 | SKF | 2.1 |
| 07f01 | CA967283 | abhydrolase domain containing 12 | SKF | 1.8 |
| 14g20 | CA964395 | abi genemember 3binding protein | SKF | -2.1 |
| 24e04 | CA969790 | ac2-210 isoform 2 | LY | 1.3 |
| 11h16 | CA966103 | acid phosphatasetartrate resistant | SKF | 1.9 |
| 07e21 | CA964021 | actin | LY | 1.3 |
| 14h02 | CA964398 | actin related protein 2 3subunit 3 | LY | 1.5 |
| 24b10 | CA969738 | actin related protein 2 3subunit41kda | LY | 1.6 |
| 20h08 | CA967040 | actin-binding lim protein 1 | SKF | 1.4 |
| 09c14 | CA964782 | acyl-coenzyme a oxidasepalmitoyl | SKF | 2 |
| 09c14 | CA964782 | acyl-coenzyme a oxidasepalmitoyl | LY | 1.3 |
| 16f09 | CF662760 | acyl-coenzyme avery long chain | LY | 1.3 |
| 07k04 | CA964061 | adducin 3 | LY | 1.3 |
| 21p13 | CA969216 | adenosine monophosphate deaminase 3 | LY | 1.6 |
| 04n13 | FG392996 | adenylate cyclase activating polypeptide 1b | SKF | 1.5 |
| 24l12 | CA969940 | adenylate kinase 1 | SKF | -1.5 |
| 24h02 | CA969848 | adenylate kinase 3-like 1 | LY | 1.5 |
| 24h02 | CA969848 | adenylate kinase 3-like 1 | SKF | -1.6 |
| 24g11 | CA969838 | adenylosuccinate lyase | LY | 1.3 |
| 11o13 | CA965981 | adipose differentiation-related protein | SKF | 2.1 |
| 09m16 | CA964881 | adp-ribosylation factor gtpase activating protein 2 | SKF | -1.5 |
| 18p01 | CA969058 | adp-ribosylation factor-like 3 | SKF | 2.1 |
| 03n15 | FG392663 | adp-ribosylation factor-like 6 interacting protein 1 | SKF | 2.3 |
| 09h20 | CA964835 | af363273_1 toxin-1 | SKF | 1.8 |
| 20h14 | CF662863 | afg3 atpase family gene 3-like 2 | LY | 1.4 |
| 23d14 | CA969576 | agmatinase | LY | 1.3 |
| 19a15 | CA967231 | aig2-like domain 1 | SKF | 1.5 |
| 19a15 | CA967231 | aig2-like domain 1 | LY | 1.3 |
| 09b18 | CA964773 | alcohol dehydrogenase 5 | SKF | 1.6 |
| 08m02 | CA968782 | alcohol dehydrogenase 5 (class iii)chi polypeptide | SKF | 1.8 |
| 08m02 | CA968782 | alcohol dehydrogenase 5 (class iii)chi polypeptide | LY | 1.5 |
| 05i24 | CF662442 | alcohol dehydrogenase class 3 | LY | 1.3 |
| 12n16 | CA965472 | aldehyde dehydrogenase 1member l2 | LY | 1.5 |
| 08k20 | CA968758 | aldehyde dehydrogenase 7member a1 | LY | 1.3 |
| 09k05 | CA964152 | aldh9a1a protein | SKF | 1.8 |
| 09g24 | CA964827 | aldo-keto reductase familymember a1 (aldehyde reductase) | SKF | 1.7 |
| 17o10 | CA966472 | aldolase a | LY | 1.6 |
| 13g14 | CF662701 | aldolase a | SKF | 2.4 |
| 22b24 | CA969260 | aldolase a | SKF | -1.7 |
| 19a22 | CA965001 | aldolase a | SKF | 1.4 |
| 07b05 | CA968448 | aldolase b | SKF | 2 |
| 06g20 | CA965426 | aldolasefructose-bisphosphate | LY | 1.6 |
| 08h06 | CA968701 | allantoicase | SKF | 1.6 |
| 11m07 | CF662642 | allantoicase | LY | 1.4 |
| 08o14 | CA968816 | alpha 1 microglobulin bikunin | SKF | 2.6 |
| 22d20 | CA969301 | alpha 2 | LY | 1.4 |
| 12i14 | CA970261 | alpha 2a pancreatic | SKF | 2.4 |
| 08k16 | CA968755 | alpha 2a pancreatic | LY | 1.6 |
| 22i20 | CA969400 | alpha 3 | LY | 1.3 |
| 13f14 | CA965474 | alpha 8 like | SKF | 1.7 |
| 20e23 | CA966276 | alpha 8 like | SKF | -1.6 |
| 22g09 | CA969352 | alpha 8 like 4 | SKF | -1.8 |
| 23g09 | CA966809 | alpha 8 like 4 | LY | 1.4 |
| 04a05 | FG392684 | alpha 8 like 4 | SKF | 1.5 |
| 22k22 | CA969438 | alpha actin | SKF | -1.8 |
| 19o15 | CA966239 | alpha actin | SKF | 1.7 |
| 22a16 | CA969232 | alpha actin | SKF | -1.9 |
| 24l20 | CA969948 | alpha actin | LY | 1.4 |
| 08p02 | CA968822 | alpha globin | SKF | 2.7 |
| 12e18 | CA970244 | alpha globin | LY | 1.4 |
| 05b05 | CA966877 | alpha-aminoadipate aminotransferase | LY | 1.3 |
| 07e01 | CA964007 | amp-activated protein kinase alpha 2 catalytic subunit | SKF | 1.6 |
| 09c15 | CA964116 | amylo-6-4-alpha-glucanotransferase isoform 1 | SKF | 1.7 |
| 22e19 | CA969319 | amyloid betaprecursor protein-familymember 2 | LY | 1.3 |
| 11f02 | CA966086 | angiopoietin-like 3 | SKF | 1.9 |
| 21d13 | CA969172 | angiotensin i converting enzyme (peptidyl-dipeptidase a) 2 | LY | 1.7 |
| 06e16 | CF662481 | angiotensinogen | LY | 1.5 |
| 09p02 | CA964907 | angiotensinogen precursor | SKF | 1.8 |
| 11h15 | CF662627 | angiotensinogen precursor | SKF | 1.6 |
| 17m11 | CA964982 | annexin a2a | SKF | -1.8 |
| 18o02 | CA964723 | anterior pharynx defective 1b homolog | SKF | 1.8 |
| 05o15 | CF662455 | antizyme inhibitor 1 | LY | 1.4 |
| 06h17 | CF662493 | aoc2 protein | LY | 1.4 |
| 10k10 | CA967684 | apobec1 complementation factor | LY | 1.7 |
| 09g06 | CA964819 | apolipoprotein a-i | SKF | 1.6 |
| 07k17 | CA964073 | apolipoprotein a-i | SKF | 1.6 |
| 10i03 | CA967660 | apolipoprotein a-i | SKF | -1.7 |
| 09e08 | CA964799 | apolipoprotein a-i | LY | 1.4 |
| 08o04 | CA968811 | apolipoprotein a-iv | SKF | 1.9 |
| 07j03 | CA966715 | apolipoprotein a-iv | SKF | 1.9 |
| 07g22 | CA964039 | apolipoprotein a-iv | LY | 1.5 |
| 09o08 | CA964899 | apolipoprotein a-iv | LY | 1.5 |
| 07k06 | CA964063 | apolipoprotein a-iv | SKF | -1.5 |
| 06o14 | CF662535 | apolipoprotein b | LY | 1.6 |
| 05b15 | CA965378 | apolipoprotein c-i | SKF | 1.6 |
| 09b20 | CA964774 | apolipoprotein c-i | LY | 1.6 |
| 10f23 | CA968871 | apolipoprotein c-i | SKF | -1.7 |
| 02p15 | DY231777 | apolipoprotein c-i | SKF | 1.5 |
| 04i14 | FG392943 | apolipoprotein c-i | SKF | 1.5 |
| 07c18 | CA964001 | apolipoprotein c-i | SKF | -1.7 |
| 16n14 | CF662778 | apolipoprotein e | SKF | 2.4 |
| 14e02 | CA964343 | apolipoprotein e | SKF | 2.2 |
| 19k05 | CA966212 | apolipoprotein e | SKF | -1.8 |
| 20c11 | CA966259 | apolipoprotein e | LY | 1.4 |
| 02l03 | DY231671 | apolipoprotein e | SKF | 1.4 |
| 17a20 | CF662782 | arachidonate 5-lipoxygenase-activating protein | SKF | -1.7 |
| 08c03 | CA968622 | arginine-mutated in early stage tumors | LY | 1.5 |
| 14k14 | CA964468 | arp2 actin-related protein 2 homolog | SKF | 2.3 |
| 14k14 | CA964468 | arp2 actin-related protein 2 homolog | LY | 1.3 |
| 06b04 | CF662465 | arrestin domain containing 3 | LY | 1.6 |
| 19m02 | CA965055 | arsenic (+3 oxidation state) methyltransferase | SKF | 2.2 |
| 12l13 | CA966040 | asf1 anti-silencing function 1 homolog b | SKF | 1.9 |
| 22g08 | CA969351 | ash2 (or homeotic)-like | SKF | -1.6 |
| 19p03 | CA969147 | asparaginase-like 1 protein | SKF | 2 |
| 23i09 | CA968043 | at rich interactive domain 2 | LY | 1.5 |
| 19k17 | CA966218 | atp synthase mitochondrial f1 complex assembly factor 1 | LY | 1.4 |
| 13g17 | CA966763 | atp synthase subunit b | SKF | -1.5 |
| 02p17 | DY231779 | atp1a3 protein | SKF | 1.4 |
| 24h14 | CA969859 | atpase h+ transporting v1 subunit g isoform 1 | SKF | -1.7 |
| 12c06 | CA970231 | atpase type 13a1 | SKF | 1.6 |
| 12c06 | CA970231 | atpase type 13a1 | LY | 1.3 |
| 12f17 | CA966012 | atp-bindingsub-family b (mdr tap)member 6 | SKF | 1.5 |
| 23l24 | CA969669 | atp-bindingsub-family b (mdr tap)member 8 | SKF | -2 |
| 10i19 | CA967672 | atp-bindingsub-family c (cftr mrp)member 1 | SKF | -1.7 |
| 19h02 | CA965822 | atp-bindingsub-family fmember 2 | SKF | 1.9 |
| 19h02 | CA965822 | atp-bindingsub-family fmember 2 | LY | 1.4 |
| 08i03 | CA968716 | atph+mitochondrial f0subunitisoform 2 | SKF | 1.7 |
| 16e05 | CA966367 | atph+mitochondrial f1delta subunit | SKF | 1.5 |
| 12e06 | CA970238 | autophagy protein 5 | LY | 1.3 |
| 21j04 |  | bardet-biedl syndrome 4 | SKF | 1.7 |
| 06n22 | CF662530 | basic helix-loop-helix domainclass2 | LY | 1.5 |
| 12p22 | CA966352 | bcl2 adenovirus e1b 19kd interacting protein 1 | LY | 1.2 |
| 21h03 | CA969184 | bcl-2 adenovirus e1b 19kd interaction protein 3c | LY | 1.4 |
| 13f13 | CF662698 | beta-2 microglobulin | SKF | 2.6 |
| 02l13 | DY231736 | beta-actin | SKF | 2.4 |
| 03o22 | DY232011 | beta-actin | SKF | -1.6 |
| 07b06 | CA968449 | beta-actin | LY | 1.4 |
| 03d03 | DY231820 | beta-amyloid precursor protein a | SKF | 1.6 |
| 22m11 | CA969470 | beta-carotene oxygenase 2a | SKF | -2 |
| 18i03 | CA964661 | beta-galactoside-binding lectin | SKF | 1.4 |
| 10f05 | CA968862 | betaine-homocysteine methyltransferase | SKF | -1.8 |
| 03k03 | DY231939 | b-gal alpha peptide | SKF | 1.8 |
| 03n02 | DY231958 | b-gal alpha peptide | SKF | 2.4 |
| 20o14 | CA965911 | biliverdin reductase b (flavin reductase) | SKF | 1.6 |
| 15l10 | CA965673 | bladder cancer associated protein | LY | 1.9 |
| 16o14 | CA966992 | bladder cancer associated protein | SKF | 1.9 |
| 17j08 | CA966515 | bladder cancer associated protein | SKF | -1.5 |
| 09h14 | CA964832 | brain protein 44 | SKF | 2.2 |
| 10k07 | CA967681 | brain protein 44 | SKF | -1.7 |
| 14f16 | CA964375 | brain protein 44-like protein | SKF | 1.5 |
| 20e19 | CA966274 | brain-specific angiogenesis inhibitor 1-associated protein 2 | LY | 1.3 |
| 04d14 | FG392797 | brain-specific angiogenesis inhibitor 3 | SKF | 1.5 |
| 11d18 | CA966083 | branched chain aminotransferasecytosolic | SKF | 1.5 |
| 22l24 | CA969461 | branched chain ketoacid dehydrogenase kinase | SKF | -1.8 |
| 18m19 | CA964717 | bridging integrator 1 | SKF | -1.7 |
| 22a18 | CA969234 | bromodomain and wd repeat domain containing 3 | LY | 1.5 |
| 22a18 | CA969234 | bromodomain and wd repeat domain containing 3 | SKF | -1.5 |
| 10h22 | CA964219 | btb and cnc homologybasic leucine zipper transcription factor 1 | LY | 1.3 |
| 20p06 | CA966562 | bud31 homolog | LY | 1.4 |
| 23j12 | CA969636 | c10 protein | SKF | -1.6 |
| 15j16 | CA965656 | c1q and tumor necrosis factor related protein 5 | SKF | 1.7 |
| 17f01 | CA965752 | c5orf4 protein | LY | 1.3 |
| 03n13 | FG392661 | calcium calmodulin-dependent protein kinase ii inhibitor 1 | SKF | 1.5 |
| 18a03 | CA964579 | calciumvoltage-beta 1 subunit isoform 2 | SKF | 1.4 |
| 21h01 | CA966781 | calmodulin 2 | SKF | 3 |
| 21h01 | CA966781 | calmodulin 2 | LY | 1.4 |
| 11b04 | CA966066 | calmodulin variant 1 | SKF | 1.8 |
| 24e09 | CA969795 | calmodulin variant 1 | LY | 1.4 |
| 11k02 | CA966988 | calponin 2 | SKF | 2.1 |
| 14d03 | CA964323 | capping protein (actin filament) muscle z-beta | SKF | 1.4 |
| 12i17 | CA970263 | carbonic anhydrase v | SKF | 1.5 |
| 09o16 | CA964903 | carboxypeptidase a1 | SKF | 1.6 |
| 15o22 | CA965717 | carboxypeptidase e | LY | 1.5 |
| 12n15 | CA966050 | cardiac myosin light chain-1 | SKF | 2.4 |
| 12n15 | CA966050 | cardiac myosin light chain-1 | LY | 1.4 |
| 05g01 | CF662434 | casein kinasealpha 1 | SKF | 1.7 |
| 03f05 | FG392563 | casein kinasealpha 1 polypeptide | SKF | 1.7 |
| 20o23 | CA967005 | caseinolytic protease x homolog | LY | 1.3 |
| 17e11 | CA964941 | cask interacting protein 1 | SKF | -1.8 |
| 07g21 | CA964038 | catalase | LY | 1.4 |
| 12b15 | CA965988 | cathepsin b | LY | 1.5 |
| 22e21 | CA969321 | cation transport regulator-like 1 | LY | 1.4 |
| 17e03 | CA964937 | caveolin 2 | SKF | -1.8 |
| 17e03 | CA964937 | caveolin 2 | LY | 1.4 |
| 23o21 | CA968107 | cayman type | LY | 1.7 |
| 23o21 | CA968107 | cayman type | SKF | -1.7 |
| 12p10 | CA966879 | ccaat enhancer binding protein beta | SKF | 1.8 |
| 12p10 | CA966879 | ccaat enhancer binding protein beta | LY | 1.4 |
| 23h10 | CA969613 | ccd72_tetngcoiled-coil domain-containing protein 72 | LY | 1.4 |
| 19h06 | CA965824 | cd4-like protein | LY | 1.4 |
| 18l21 | CA969037 | cd59 glycoprotein precursor | LY | 1.7 |
| 19c14 | CA965009 | cd59 glycoprotein precursor | SKF | 2.3 |
| 18l22 | CA969038 | cd59 glycoprotein precursor | SKF | -1.6 |
| 10m24 | CA967710 | cd9 antigen | SKF | -2.1 |
| 19f05 | CA969101 | cdp-diacylglycerol--inositol 3-phosphatidyltransferase (phosphatidylinositol synthase) | SKF | 2 |
| 23g02 | CA968017 | cdp-diacylglycerol--inositol 3-phosphatidyltransferase (phosphatidylinositol synthase) | SKF | -1.8 |
| 23i18 | CA968049 | cell adhesion molecule with homology to l1cam | SKF | -1.6 |
| 08a06 | CA966754 | cell cycle associated protein 1 | LY | 1.6 |
| 08a06 | CA966754 | cell cycle associated protein 1 | SKF | 1.5 |
| 06g06 | CF662485 | cellular myelocytomatosis oncogene | SKF | -2.7 |
| 18p16 | CA969072 | centromere protein b | SKF | -1.7 |
| 24c20 | CA969767 | cerebellin 1 precursor | SKF | -1.6 |
| 15b22 | CA965560 | cg18480 cg18480-pa | LY | 1.5 |
| 09c13 | CF662574 | chaperonin containingsubunit 3 | SKF | 2.1 |
| 09a09 | CA964108 | chemokine (c-c motif) ligand 13 | LY | 1.4 |
| 14l02 | CA964480 | chemokine ck-1 | SKF | 2.2 |
| 12b23 | CA965992 | chemokine cxcl-c1c | LY | 1.3 |
| 10i10 | CA970450 | chk1 checkpoint-like protein | SKF | -1.7 |
| 15g21 | CA967026 | chmpmember 7 | SKF | -1.8 |
| 14d15 | CA964334 | chondroitin sulfatect-2 | SKF | 2 |
| 20i01 | CA966287 | chromatin accessibility complex 1 | LY | 1.5 |
| 16l16 | CA965494 | chromatin modifying protein 1a | SKF | 1.6 |
| 20g14 | CA965884 | chromatin modifying protein 1b | LY | 1.4 |
| 08i02 | CA968715 | chromobox homolog 3 | SKF | 3 |
| 08i02 | CA968715 | chromobox homolog 3 | LY | 1.3 |
| 11n07 | CF662649 | chromosome 1 open reading frame 9 | SKF | 1.7 |
| 22p01 | CA969525 | chromosome 1 open reading frameisoform cra_a | LY | 1.5 |
| 06b15 | CA965408 | chromosome 13 open reading frame 1 | LY | 1.6 |
| 21i15 | CA967938 | chromosome 16 open reading frame 62 protein | SKF | 1.9 |
| 21i15 | CA967938 | chromosome 16 open reading frame 62 protein | LY | 1.3 |
| 15c12 | CA965567 | chromosome 17 open reading frame 75 | LY | 1.3 |
| 06n20 | CF662528 | chromosome 20 open reading frame 108 | LY | 1.5 |
| 13k19 | CA967860 | chromosome 20 open reading frame 149 | LY | 1.3 |
| 12f14 | CA966347 | chromosome 20 open reading frame 43 | SKF | 2 |
| 11o18 | CA965465 | chromosome 22 open reading frame 9 | LY | 1.3 |
| 22f20 | CA969340 | chromosome 5 open reading frame 28 | LY | 1.4 |
| 06c14 | CF662470 | chromosome 6 open reading frame 58 | LY | 1.4 |
| 18l13 | CA969030 | chromosome 8 open reading frame 40 | SKF | 1.9 |
| 18l04 | CA969023 | chromosome 9 open reading frame 16 | SKF | 1.5 |
| 18g24 | CA964658 | chromosome 9 open reading frame 25 | SKF | -1.5 |
| 16e02 | CA966153 | chromosome 9 open reading frame 82 | SKF | 1.6 |
| 15j13 | CA965653 | chromosome 9 open reading frame 86 | SKF | 2.8 |
| 08k22 | CA968760 | chymotrypsinogen b1 | SKF | 1.5 |
| 06i12 | CF662498 | chymotrypsinogen b1 | SKF | -2 |
| 06i12 | CF662498 | chymotrypsinogen b1 | LY | 1.4 |
| 15l11 | CA965674 | cisplatin resistance related protein crr9p | LY | 1.5 |
| 17n01 | CA965782 | citrate synthase | SKF | 1.6 |
| 20a08 | CA965860 | clasp1 protein | LY | 1.3 |
| 14g01 | CA964745 | claudin 23 | SKF | 1.8 |
| 22d16 | CA969298 | cleavage stimulation3 pre-subunit77kda | SKF | -1.7 |
| 04e13 | FG392808 | clusterin | SKF | 1.7 |
| 24h08 | CA967007 | clusterin | LY | 1.5 |
| 22h02 | CA969366 | cnksr family member 3 | SKF | -2.2 |
| 07n23 | CA968571 | coagulation factor vii | LY | 1.5 |
| 18c21 | CA964616 | coatomer proteinsubunit epsilon | SKF | -1.8 |
| 15b15 | CA965554 | coiled-coil domain containing 3 | SKF | 1.7 |
| 14k02 | CA964457 | coiled-coil domain containing 47 | SKF | 2.1 |
| 12k08 | CA970270 | coiled-coil domain containing 49 | LY | 1.5 |
| 09n22 | CA964894 | coiled-coil domain containing 80 | SKF | -1.8 |
| 09d02 | CA964787 | coiled-coil domain containing 94 | LY | 1.4 |
| 19d01 | CA969088 | comm domain containing 10 | SKF | 3.1 |
| 24j10 | CA969898 | comm domain containing 3 | LY | 1.4 |
| 11h10 | CF662626 | complement c3-h1 | LY | 1.7 |
| 09h15 | CF662584 | complement c3-h1 | SKF | 1.7 |
| 09d07 | CF662577 | complement c3-h1 | SKF | 1.6 |
| 09p08 | CA964909 | complement c3-h1 | LY | 1.5 |
| 08l02 | CA968762 | complement c3-h1 | SKF | 1.4 |
| 07a14 | CA966737 | complement c3-h1 | SKF | 1.6 |
| 09f08 | CA964808 | complement c3-h1 | LY | 1.3 |
| 08o15 | CA970421 | complement component c3 | SKF | 1.6 |
| 08k14 | CA968754 | complement component c5 | SKF | 2.9 |
| 08o02 | CA968810 | complement componentq subcomponent-like 4 | SKF | 1.5 |
| 08b20 | CA968617 | complement componentq subcomponent-like 4 | LY | 1.3 |
| 21m13 | CA967957 | complement componentqc chain | SKF | 1.5 |
| 08c17 | CA970381 | complement factor b | SKF | 1.9 |
| 06p18 | CA967018 | complement factor b | SKF | -1.7 |
| 24a18 | CA969724 | cop9 constitutive photomorphogenic homolog subunit 3 | SKF | -1.5 |
| 15b05 | CA965550 | cop9 constitutive photomorphogenic homolog subunit 4 | SKF | 1.7 |
| 02a14 | DY231591 | corticotropin releasing hormone | SKF | 1.7 |
| 03n17 | FG392664 | corticotropin releasing hormone binding protein | SKF | 1.5 |
| 12c10 | CA970233 | cox16 cytochrome c oxidase assembly homolog | LY | 1.4 |
| 09m05 | CA964163 | cpb1 protein | LY | 1.5 |
| 02c23 | DY231608 | creatine kinase b variant 1 | SKF | -1.6 |
| 13o09 | CA967875 | creatine kinase b variant 1 | LY | 1.5 |
| 04h13 | FG392841 | creatinebrain | SKF | 2.6 |
| 14h03 | CA964399 | creatinemuscle a | SKF | 1.7 |
| 22g22 | CA969363 | creatinemuscle a | LY | 1.5 |
| 14a04 | CA964273 | creatinemuscle a | LY | 1.4 |
| 19a04 | CA964993 | csde1 protein | SKF | 1.5 |
| 19a04 | CA964993 | csde1 protein | LY | 1.3 |
| 19o06 | CA965066 | c-terminal binding protein 1 | SKF | -1.6 |
| 18c13 | CA964608 | c-type natriuretic peptide-1 | SKF | 1.7 |
| 19a14 | CA964997 | cubilin (intrinsic factor-cobalamin receptor) | SKF | 1.4 |
| 15m10 | CA966748 | cullin 1 | LY | 1.5 |
| 11a04 | CF662606 | cut-like homeobox 1 | SKF | 1.5 |
| 08k02 | CA968746 | cwc15 homolog | SKF | 2.1 |
| 08k02 | CA968746 | cwc15 homolog | LY | 1.3 |
| 17g09 | CA964951 | cxxc finger 1 (phd domain) | SKF | -1.7 |
| 07b18 | CA968460 | cyclin g2 | LY | 1.4 |
| 20c14 | CA965871 | cyclin-dependent kinase 2 | LY | 1.3 |
| 11n16 | CF662651 | cystathionase (cystathionine gamma-lyase) | SKF | 2 |
| 15m06 | CA966732 | cystatin precursor | LY | 1.6 |
| 08k17 | CA968756 | cystatin precursor | SKF | 1.5 |
| 05m04 | CA967567 | cysteine conjugate-beta lyase 2 | LY | 1.2 |
| 21a16 | CA966524 | cysteine-rich pdz-binding protein | LY | 1.3 |
| 13a01 | CF662681 | cysteinetype i | LY | 1.4 |
| 24l14 | CA969942 | cytochrome b reductase 1 | LY | 1.4 |
| 11e02 | CA965460 | cytochrome b5 | SKF | 1.8 |
| 08i08 | CA968719 | cytochrome c oxidase polypeptide viii-mitochondrial precursor | LY | 1.4 |
| 04a09 | FG392687 | cytochrome c oxidase subunit ii | SKF | -1.6 |
| 18i13 | CA964671 | cytochrome c oxidase subunit vb | SKF | 1.5 |
| 03p18 | DY232013 | cytochrome c oxidase subunit vib isoform 1 | SKF | -1.5 |
| 11e14 | CF662619 | cytochrome c oxidase subunit viia polypeptide 2 like | SKF | 2 |
| 21d15 | CA967006 | cytochrome c oxidase subunit viia polypeptide 2 like | SKF | -1.5 |
| 12f21 | CA966014 | cytochrome c oxidase subunit viia polypeptide 2 like | LY | 1.3 |
| 12c16 | CA966760 | cytochrome c oxidase subunitmitochondrial precursor | SKF | 1.6 |
| 20o15 | CA967237 | cytochrome c-1 | SKF | 1.9 |
| 17n23 | CA966996 | cytochrome c-1 | SKF | -1.6 |
| 11i13 | CA965958 | cytochrome csubunit va | SKF | 1.7 |
| 08l16 | CA968773 | cytochrome csubunit viia 2 | SKF | 1.7 |
| 08l16 | CA968773 | cytochrome csubunit viia 2 | LY | 1.4 |
| 07m14 | CA964087 | cytochrome p450 like | SKF | 2.1 |
| 06d13 | CA965416 | cytochrome p450 like | LY | 1.6 |
| 05c09 | CA967490 | cytochromefamilysubfamilypolypeptide 2 | SKF | -1.6 |
| 24o05 | CA969991 | cytohesin 1 | SKF | -1.6 |
| 24o05 | CA969991 | cytohesin 1 | LY | 1.4 |
| 05l01 | CA966987 | cytokine induced apoptosis inhibitor 1 | SKF | 2.3 |
| 15m08 | CA965689 | dazap1 protein | SKF | -1.6 |
| 22e09 | CA969311 | d-dopachrome tautomerase | LY | 1.3 |
| 23b20 | CA969559 | dead (asp-glu-ala-asp) box polypeptide 17 | LY | 1.3 |
| 19f06 | CA965817 | dead (asp-glu-ala-asp) box polypeptide 46 | LY | 1.3 |
| 16g04 | CA966158 | dead (asp-glu-ala-asp) box polypeptide 56 | SKF | 1.8 |
| 24c04 | CA969754 | deah (asp-glu-ala-his) box polypeptide 8 | LY | 1.4 |
| 19k15 | CA966217 | death associated protein 3 | SKF | 1.9 |
| 08k18 | CA968757 | dedd1 protein | SKF | 1.4 |
| 08k18 | CA968757 | dedd1 protein | LY | 1.3 |
| 12f07 | CA966007 | dedicator of cyto-kinesispartial | SKF | 1.9 |
| 23k20 | CA968071 | dehydrogenase reductase (sdr family) member 1 | SKF | -1.7 |
| 23k20 | CA968071 | dehydrogenase reductase (sdr family) member 1 | LY | 1.3 |
| 12e05 | CA967749 | dehydrogenase reductase (sdr family) member 7 | SKF | 1.9 |
| 08f19 | CA968680 | dehydrogenase reductase sdr family member 12 | LY | 1.3 |
| 08k08 | CA970401 | delta-6 fatty acyl desaturase | LY | 1.3 |
| 15d15 | CA965582 | delta-aminolevulinic acid dehydratase | SKF | 1.8 |
| 15d15 | CA965582 | delta-aminolevulinic acid dehydratase | LY | 1.3 |
| 06n21 | CF662529 | delta-synthetase 1 | LY | 1.4 |
| 14p01 | CA964557 | denn madd domain containing 1a | SKF | 1.9 |
| 16n15 | CA965500 | density-regulated protein | SKF | 2.8 |
| 23b11 | CA969551 | density-regulated protein | LY | 1.4 |
| 12d01 | CA965993 | deoxycytidine kinase | SKF | 2.3 |
| 16m01 | CA965496 | developmentally regulated gtp binding protein 2 | SKF | 2 |
| 05m01 | CA967565 | dihydrolipoamide dehydrogenase | SKF | 1.7 |
| 14p22 | CA964574 | dihydrolipoamide dehydrogenase | SKF | -1.7 |
| 10c08 | CA967614 | dihydrolipoamide s-acetyltransferase (e2 component of pyruvate dehydrogenase complex) | SKF | -1.6 |
| 12h01 | CA966016 | dipeptidylpeptidase 8 | SKF | 1.7 |
| 17f15 | CA965758 | domain containing 2 | SKF | -1.6 |
| 03l04 | DY231945 | dopa decarboxylase | SKF | 1.8 |
| 03o12 | DY231965 | dopamine beta-hydroxylase | SKF | -1.6 |
| 03l12 | DY231949 | dopamine d3 receptor | SKF | 1.4 |
| 03j04 | DY231937 | dopamine receptor d1 | SKF | 1.9 |
| 03n12 | DY231961 | dopamine transporter | SKF | 1.5 |
| 18c17 | CA964612 | doublecortin-like kinase 1 | SKF | 1.4 |
| 22j10 | CA969411 | downregulated in ovarian cancer 1 isoform 2 | LY | 1.4 |
| 08e17 | CA970385 | dr1-associated protein 1 (negative cofactor 2 alpha) | SKF | 1.6 |
| 24c06 | CA969755 | dynactin 6 | LY | 1.5 |
| 18j22 | CA969018 | dynein light chain lc8-type 2 | SKF | -1.5 |
| 20g08 | CA965882 | dystrobrevin binding protein 1 | LY | 1.6 |
| 14o02 | CA964538 | egl nine homolog 1 | SKF | 2.7 |
| 09i08 | CA964840 | elastase-like serine protease | SKF | 2.7 |
| 09i08 | CA964840 | elastase-like serine protease | LY | 1.7 |
| 09j01 | CF662587 | elastasepancreatic | SKF | 2.2 |
| 06f20 | CF662483 | elongation factor 1-alpha | LY | 1.5 |
| 12a09 | CA967733 | elongation factor-2 kinase | LY | 1.3 |
| 10g16 | CA967654 | elongation of very long chain fatty acids (fen1 sur4 yeast)-like 1 | LY | 1.3 |
| 03b05 | DY231800 | enolase | SKF | 1.4 |
| 14b18 | CA964298 | enolasebeta muscle | LY | 1.3 |
| 12d21 | CA966002 | enoyl coenzyme a hydrataseperoxisomal | LY | 1.3 |
| 24c23 | CA969770 | eosinophil chemotactic cytokine | LY | 1.4 |
| 21e20 | CF662886 | eosinophil chemotactic cytokine | LY | 1.4 |
| 02j15 | DY231716 | ependymin-II | SKF | 1.7 |
| 14o06 | CA964540 | ependymin | LY | 1.6 |
| 03o21 | DY232010 | ependymin | SKF | -1.7 |
| 02i24 | DY231713 | ependymin-I | SKF | -1.6 |
| 24a12 | CA969719 | eph receptor a7 | SKF | -2.1 |
| 19k02 | CA965044 | epoxide hydrolasecytoplasmic | SKF | 2.9 |
| 15a10 | CA965545 | equilibrative nucleoside transporter 1 | SKF | -1.6 |
| 11e01 | CA965933 | er lipid raft associated 2 | SKF | 1.8 |
| 02f15 | AF177465 | estrogen receptor beta 2 | SKF | 2 |
| 09h08 | CF662582 | ets variant gene 6 (tel oncogene) | SKF | -1.6 |
| 05e11 | CA967511 | eukaryotic translation elongation factor 1 alpha 1 | SKF | -1.8 |
| 04c03 | FG392704 | eukaryotic translation elongation factor 1 delta (guanine nucleotide exchange protein) | SKF | 1.5 |
| 07b01 | CA966738 | eukaryotic translation elongation factor 1 gamma | SKF | 1.7 |
| 05f07 | CA965388 | eukaryotic translation elongation factor 1 gamma | LY | 1.3 |
| 06a19 | CA965406 | eukaryotic translation initiation factor 1b | LY | 1.6 |
| 10d03 | CA968850 | eukaryotic translation initiation factor 1b | SKF | -1.6 |
| 11e23 | CA965942 | eukaryotic translation initiation factor 4e | LY | 1.5 |
| 20j14 | CA966561 | eukaryotic translation initiation factorsubunit 1 alpha | SKF | 2.3 |
| 19m10 | CA965059 | eukaryotic translation initiation factorsubunit 10 | LY | 1.4 |
| 10n04 | CA964246 | eukaryotic translation initiation factorsubunit g | SKF | -1.5 |
| 15d02 | CA965572 | eukaryotic translation initiation factorsubunit h | SKF | 1.8 |
| 15d02 | CA965572 | eukaryotic translation initiation factorsubunit h | LY | 1.4 |
| 12b11 | CA965987 | f11 receptor | LY | 1.3 |
| 09p05 | CF662595 | family with sequence similaritymember a | SKF | 1.5 |
| 21i13 | CA967937 | family with sequence similaritymember b | SKF | 2 |
| 06g21 | CA965427 | family with sequence similaritymember b | LY | 1.5 |
| 20m17 | CA966317 | fast myotomal muscle troponin-t-2 | SKF | -1.6 |
| 24k10 | CA969920 | fatty acid binding proteinintestinal | LY | 1.3 |
| 11p02 | CA966136 | fatty acid binding proteinliver | SKF | 1.7 |
| 10g02 | CA970443 | fatty acid binding proteinliver basic | SKF | -1.5 |
| 08a17 | CA968596 | fatty acid binding proteinliver basic | LY | 1.4 |
| 08k05 | CA968748 | fatty acid desaturase 2 | SKF | 1.5 |
| 09c18 | CA964784 | fau | SKF | 1.7 |
| 22l23 | CA969460 | f-box only protein 21 | SKF | -2.2 |
| 21b10 | CF662881 | f-box protein 48 | LY | 1.6 |
| 16f17 | CA966401 | ferritin heavy chain | LY | 1.3 |
| 15h01 | CA965620 | fetal globin inducing factor | SKF | 2.6 |
| 06b16 | CF662468 | fetuin family protein precursor | LY | 1.6 |
| 06m15 | CF662519 | fibrinogen gamma polypeptide | LY | 1.5 |
| 09e01 | CA964120 | fibronectin 1 | SKF | 2 |
| 09e01 | CA964120 | fibronectin 1 | LY | 1.3 |
| 10c01 | CA967610 | fk506 binding protein25kda | SKF | 1.4 |
| 13f07 | CF662696 | fk506 binding protein25kda | LY | 1.4 |
| 24j21 | CA966789 | fk506-binding protein 1a | SKF | -1.5 |
| 20e05 | CA966267 | folylpolyglutamate synthase | LY | 1.3 |
| 05p01 | CF662456 | formaldehyde dehydrogenase | SKF | 1.5 |
| 09k09 | CA964153 | formiminotransferase cyclodeaminase | LY | 1.5 |
| 06b05 | CF662466 | fructose--bisphosphatase 1 | LY | 1.5 |
| 05l15 | CF662445 | fructose--bisphosphatase 1 | SKF | -1.6 |
| 21p15 | CA969217 | fructose-bisphosphate aldolase c | SKF | 3.2 |
| 03o09 | FG392624 | fructose-bisphosphate aldolase c | SKF | -1.6 |
| 21p15 | CA969217 | fructose-bisphosphate aldolase c | LY | 1.3 |
| 13g10 | CA967121 | fth1 protein | LY | 1.9 |
| 15e04 | CA965532 | fumarate hydratase | SKF | 1.4 |
| 24d21 | CA969786 | g protein-coupled receptor 108 | LY | 1.4 |
| 15h17 | CA965628 | g protein-coupled receptor 141 | SKF | 1.9 |
| 10m11 | CA967701 | g protein-coupledfamilygroupmember c | SKF | -1.6 |
| 17d17 | CA967028 | galactocerebrosidase | SKF | -1.5 |
| 21o14 | CA966569 | galactose mutarotase (aldose 1-epimerase) | SKF | 2.1 |
| 03l02 | FG392606 | galanin | SKF | 1.8 |
| 03n01 | DY231957 | galanin | SKF | 1.5 |
| 04p14 | FG393018 | gamma 1 | SKF | 1.8 |
| 14d02 | CA964322 | gamma-aminobutyric acid receptor-associated1 | SKF | 2.3 |
| 14d02 | CA964322 | gamma-aminobutyric acid receptor-associated1 | LY | 1.3 |
| 23n12 | CA969678 | gamma-aminobutyric acidaalpha 5 | SKF | -1.7 |
| 03b02 | DY231797 | gamma-glutamyl cyclotransferase | SKF | 2.4 |
| 02l17 | DY231739 | gamma-glutamyl cyclotransferase | SKF | 1.8 |
| 19l21 | CA969138 | gamma-glutamyl cyclotransferase | LY | 1.6 |
| 03j24 | FG392643 | gamma-glutamyl cyclotransferase | SKF | -1.6 |
| 17n11 | CA965786 | gamma-glutamyl cyclotransferase | SKF | -1.7 |
| 20e06 | CA966778 | gamma-glutamyl hydrolase | LY | 1.4 |
| 04g03 | FG392747 | gdp dissociation inhibitor 2 | SKF | 1.5 |
| 06i05 | CF662496 | gelsolin (finnish type) | LY | 1.5 |
| 22d15 | CA969297 | gene model | LY | 1.5 |
| 20o10 | CA965909 | general transcription factorpolypeptide30kda | LY | 1.8 |
| 20o10 | CA965909 | general transcription factorpolypeptide30kda | SKF | -2.2 |
| 04k07 | FG392878 | ghrh pacap precursor | LY | 1.6 |
| 02b01 | L23876 | glial fibrillary acidic protein | SKF | 1.9 |
| 09b15 | CF662572 | glucan (-alpha-)branching enzyme 1 | SKF | 2.2 |
| 21d23 |  | glucose-6-phosphate isomerase | LY | 1.6 |
| 02b02 | DY231527 | glutamate decarboxylase 1 | SKF | 1.8 |
| 02f01 | DY231566 | glutamate decarboxylase 2 | SKF | 3 |
| 03i02 | DY231932 | glutamate ampa 2a | SKF | 2.4 |
| 03j01 | FG392592 | glutamate ampa 2a | SKF | 2.4 |
| 03o02 | FG392621 | glutamate ampa 2a | LY | 1.3 |
| 03i01 | DY231931 | glutamate ampa 3 | SKF | 1.5 |
| 20m06 | CA965899 | glutamate kainate 2 | SKF | 1.4 |
| 11l11 | CA966342 | glutamate n-methyl d-aspartate 1a | SKF | -1.8 |
| 19d16 | CA965811 | glutamic pyruvate transaminase (alanine aminotransferase) 2 | SKF | 2 |
| 06c24 | CA965414 | glutamic pyruvate transaminase (alanine aminotransferase) 2 | LY | 1.3 |
| 08k15 | CA970404 | glutamic-oxaloacetic transaminasemitochondrial (aspartate aminotransferase 2) | SKF | 1.8 |
| 06i09 | CA967016 | glutamic-oxaloacetic transaminasesoluble (aspartate aminotransferase 1) | LY | 1.5 |
| 15k14 | CA965665 | glutaminase | SKF | 1.9 |
| 04p13 | FG393017 | glutamine synthetase | SKF | 1.9 |
| 05g15 | CA967530 | glutaryl-coenzyme a dehydrogenase | SKF | -1.6 |
| 08b02 | CA968603 | glutathione peroxidase 3 | SKF | 1.7 |
| 10d04 | CA964192 | glutathione peroxidase 3 | SKF | -1.5 |
| 11b22 | CA966074 | glutathione s-theta 3 | LY | 1.4 |
| 10j22 | CA964231 | glutathione s-transferase | SKF | -1.6 |
| 10c06 | CA967612 | glutathione s-transferase m3 | SKF | -1.5 |
| 14d01 | CA964321 | glyceraldehyde-3-phosphate dehydrogenase | LY | 1.7 |
| 07h15 | CA968515 | glyceraldehyde-3-phosphate dehydrogenase | SKF | 1.6 |
| 23o12 | CA968103 | glyceraldehyde-3-phosphate dehydrogenase | SKF | -2.1 |
| 02p13 | DY231775 | glyceraldehyde-3-phosphate dehydrogenase | SKF | 1.6 |
| 14h21 | CA964412 | glyceraldehyde-3-phosphate dehydrogenase | SKF | -1.6 |
| 13o08 | CA966396 | glyceraldehyde-3-phosphate dehydrogenase | SKF | -1.6 |
| 13c09 | CA967821 | glyceraldehyde-3-phosphate dehydrogenase | SKF | -1.5 |
| 24f03 | CA969810 | glycerol-3-phosphate dehydrogenase | LY | 1.5 |
| 08h01 | CA968696 | glyceronephosphate o-acyltransferase | SKF | 2.2 |
| 24p12 | CF662923 | glycerophosphodiester phosphodiesterase domain containing 1 | SKF | -2.4 |
| 18k04 | CA964685 | glycogen phosphorylase | SKF | -1.6 |
| 15g24 | CF662733 | glycogenin 1 | SKF | -1.6 |
| 14j22 | CA964454 | glycyl-trna synthetase | SKF | -1.6 |
| 09d04 | CA964788 | glyoxylate reductase hydroxypyruvate reductase | SKF | 2.3 |
| 10j05 | CA968885 | glyoxylate reductase hydroxypyruvate reductase | SKF | -1.5 |
| 20b05 | CF662846 | gnas complex locus | LY | 1.5 |
| 17j10 | CF662796 | gram domain containing 3 | LY | 1.7 |
| 08c20 | CA968631 | g-rich rna sequence binding factor 1 | LY | 1.4 |
| 20i14 | CA965891 | groucho 2 | SKF | 2.2 |
| 13n09 | CA966360 | growth and transformation-dependent protein | SKF | -1.6 |
| 12g03 | CA967760 | growth hormone inducible transmembrane protein | SKF | 1.9 |
| 08g21 | CA970394 | gtp binding protein 4 | SKF | -1.7 |
| 09c23 | CA964119 | gtp binding protein 4 | SKF | 1.4 |
| 09g15 | CA964135 | guanine deaminase | SKF | 1.8 |
| 11e05 | CA965935 | guanine nucleotide binding protein (g protein)beta polypeptide 2-like 1 | SKF | 1.5 |
| 14f05 | CA964366 | guanine nucleotide binding protein (g protein)beta polypeptide 2-like 1 | SKF | 1.4 |
| 09j22 | CA964852 | guanine nucleotide binding3 | SKF | -1.5 |
| 17h09 | CA965763 | guanine nucleotide bindingalpha activating polypeptide o | SKF | -1.5 |
| 12g02 | CA970247 | guanosine monophosphate reductase 2 | LY | 1.4 |
| 02a03 | DY231518 | h+lysosomal 56 v1 subunit b2 | SKF | 1.5 |
| 03k13 | FG392644 | h+lysosomal accessory protein 2 | SKF | 1.4 |
| 23p04 | CA969690 | h+lysosomalv0 subunit c | LY | 1.5 |
| 04d02 | FG392714 | h+lysosomalv1 subunit a | SKF | 2.1 |
| 19m14 | CA965061 | h2a histonemember y2 | SKF | 1.6 |
| 11d22 | CA966085 | hd domain-containing protein 3 | LY | 1.5 |
| 19d05 | CA969090 | heart alpha-kinase | SKF | 1.7 |
| 23k05 | CF662915 | heat shock 27kda protein 2 | LY | 1.7 |
| 14d21 | CA964339 | heat shock protein 30 | SKF | -1.6 |
| 23i10 | CA968044 | heat shock protein 8 | LY | 1.6 |
| 09k16 | CA964860 | heat shock protein 90 | SKF | 1.7 |
| 14k03 | CA964458 | heat shock protein 90 beta | SKF | 1.7 |
| 05p12 | CF662458 | heat shock protein 90 beta | SKF | -1.6 |
| 14i04 | CA964417 | hect domain containing 1 | SKF | 1.5 |
| 15k22 | CF662739 | hematological and neurological expressed 1 | LY | 1.7 |
| 11a05 | CA965915 | heme responsive gene 1 | SKF | -1.8 |
| 08n23 | CA968808 | heme-binding protein 2 | SKF | 1.4 |
| 08n23 | CA968808 | heme-binding protein 2 | LY | 1.3 |
| 17e19 | CA964944 | hemoglobin subunit alpha-d | LY | 1.4 |
| 20i13 | CA966293 | heterogeneous nuclear ribonucleoprotein a b | SKF | 1.9 |
| 05d10 | CF662422 | heterogeneous nuclear ribonucleoprotein c (c1 c2) | LY | 1.4 |
| 24d12 | CA967242 | heterogeneous nuclear ribonucleoprotein k | SKF | -1.8 |
| 20h13 | CF662862 | heterogeneous nuclear ribonucleoprotein m | LY | 1.5 |
| 24o12 | CA969997 | hexokinase 1 | SKF | -1.9 |
| 21l23 | CA969208 | hexokinase 1 | LY | 1.3 |
| 08g14 | CA968690 | high density lipoproteinbinding protein | SKF | 1.6 |
| 17a06 | CF662781 | hira interacting protein 5 | SKF | -1.4 |
| 17g21 | CA964956 | histone | LY | 1.8 |
| 10h08 | CA964212 | histone 2b | SKF | -1.5 |
| 08h08 | CA968702 | histone clusterh2bb | SKF | 1.5 |
| 11p15 | CF662660 | hiu hydrolase | SKF | 2.3 |
| 11a14 | CA965451 | homolog 1 | SKF | 1.9 |
| 11d16 | CA966082 | huntingtin interacting protein 1 | SKF | 2.6 |
| 12i16 | CA970262 | hyaluronan binding protein 2 | SKF | 1.8 |
| 06a17 | CF662461 | hydroxyacyl-coenzyme amitochondrial precursor | LY | 1.4 |
| 06o10 |  | hydroxypyruvate isomerase homolog | LY | 1.5 |
| 19d02 | CA965806 | hydroxysteroid (17-beta) dehydrogenase 10 | SKF | 2.2 |
| 19d02 | CA965806 | hydroxysteroid (17-beta) dehydrogenase 10 | LY | 1.3 |
| 08b22 | CA968619 | hydroxysteroid (17-beta) dehydrogenase 12 | SKF | -1.7 |
| 09e24 | CA964806 | hydroxysteroid (17-beta) dehydrogenase 4 | LY | 1.3 |
| 05a22 | CA965509 | hydroxysteroid dehydrogenase like 2 | LY | 1.8 |
| 12c05 | CA967739 | hypothetical loc792613 | LY | 1.3 |
| 12n02 | CA966426 | hypoxanthine phosphoribosyltransferase 1 | LY | 1.6 |
| 12b08 | CA966422 | hypoxanthine phosphoribosyltransferase 1 | SKF | 1.8 |
| 22l14 | CA969453 | iclp2 protein | LY | 1.4 |
| 15h04 | CA965623 | ikaros family zinc finger 5 | SKF | 1.8 |
| 18i10 | CA964668 | immediate early response 3 interacting protein 1 | SKF | -1.5 |
| 20i09 | CA966291 | immunoglobulin d | LY | 1.8 |
| 09o24 | CA964906 | immunoglobulin mu binding protein 2 | SKF | 1.6 |
| 03i10 | FG392590 | immunoglobulin mu heavy chain | SKF | -1.5 |
| 18k24 | CA964701 | immunoglobulin z heavy chain | SKF | -1.6 |
| 08k12 | CA968753 | imp2 inner mitochondrial membrane peptidase-like | LY | 1.4 |
| 09f04 | CA967020 | importin 7 | SKF | 1.8 |
| 09o13 | CA966717 | inhbb protein | SKF | 1.8 |
| 14k05 | CA964460 | inhibitor of dna binding 3 | SKF | 1.5 |
| 04n05 | FG392910 | inhibitor of dna bindingdominant negative helix-loop-helix protein | SKF | 1.6 |
| 12i22 | CA970265 | inhibitor of dna bindingdominant negative helix-loop-helix protein | LY | 1.3 |
| 22p21 | CA969540 | inhibitor of kappa light polypeptide gene enhancer in b-kinase beta | LY | 1.6 |
| 13a07 | CA967810 | inositol polyphosphate 4-phosphatase | SKF | -1.6 |
| 20m07 | CA966312 | inositol polyphosphate-5-phosphatase f | LY | 1.3 |
| 24k12 | CA969921 | inositol-triphosphate receptor 2 | SKF | -1.5 |
| 19i02 | CA965033 | insulin induced gene 1 | SKF | 1.5 |
| 19i02 | CA965033 | insulin induced gene 1 | LY | 1.3 |
| 06n06 | CF662524 | integrin alpha l | LY | 1.5 |
| 24i12 | CA969878 | integrin beta 1 binding protein 3 | SKF | -1.7 |
| 06k20 | CA965195 | inter-alpha trypsinheavy chain 3 | LY | 1.5 |
| 09l14 | CA964869 | inter-alphainhibitor h5-like | SKF | 2.5 |
| 04p17 | FG393021 | interferon-related developmental regulator 1 | SKF | 1.7 |
| 15e18 | CA965533 | interferon-related developmental regulator 1 | LY | 1.5 |
| 15m16 | CA965691 | interleukin 22alpha 1 | SKF | -1.5 |
| 12d02 | CA966343 | interleukin 6 receptor | SKF | 1.6 |
| 06j01 | CF662504 | interleukin enhancer binding factor 2 | LY | 1.5 |
| 18o14 | CA964734 | intraflagellar transport 81 homolog | SKF | 1.5 |
| 12h23 | CA966026 | intraflagellar transport protein ift20 | LY | 1.3 |
| 09c10 | CA964780 | iodothyronine deiodinase type i | LY | 1.4 |
| 11b16 | CA966072 | iodothyronine deiodinase type i | SKF | 1.5 |
| 08g18 | CA968692 | iron-sulfur cluster scaffold homolog | SKF | 1.6 |
| 19a05 | CA966169 | iron-sulfur cluster scaffold homolog | LY | 1.4 |
| 11l15 | CA967220 | isoform cra_a | SKF | 2.1 |
| 05c04 | CA967486 | isoform cra_a | SKF | 1.4 |
| 09k15 | CA964156 | isoform cra_b | SKF | 2.1 |
| 14j02 | CA964435 | isoform cra_e | SKF | 3 |
| 23k24 | CA968074 | itchy e3 ubiquitin protein ligase homolog | SKF | -1.6 |
| 08e13 | CA968659 | j-type co-chaperone hsc20 | SKF | 1.9 |
| 08e13 | CA968659 | j-type co-chaperone hsc20 | LY | 1.3 |
| 04p02 | FG392931 | jumonji domain containing 3 | SKF | 1.7 |
| 03k15 | FG392646 | jumonji domain containing 3 | SKF | 1.5 |
| 04j23 | FG392963 | jumonji domain containing 3 | SKF | -1.5 |
| 15i19 | CA966728 | jun b proto-oncogene | LY | 1.5 |
| 04d05 | FG392717 | kainate binding protein | SKF | 1.4 |
| 16e07 | CF662756 | katanin p60 subunit a-like 1 | LY | 1.6 |
| 17l04 | CA966554 | kelch-like 31 | LY | 1.4 |
| 21l05 | CA969202 | kelch-like 6 | SKF | 1.5 |
| 07c11 | CA963995 | keratin 8 | LY | 1.4 |
| 07d11 | CA968477 | keratin 8 | SKF | 1.4 |
| 20k23 | CA966309 | kiaa0999 protein | LY | 1.4 |
| 20d12 | CF662851 | kin of irre like 3 | SKF | -1.6 |
| 18b04 | CA968929 | kti12chromatin associated | LY | 1.3 |
| 12h24 | CA966348 | kynureninase (l-kynurenine hydrolase) | SKF | -1.7 |
| 08o06 | CA968812 | lactate dehydrogenase b | SKF | 1.7 |
| 10h24 | CA964220 | lactate dehydrogenase b | LY | 1.3 |
| 20n02 | CA967004 | lactate dehydrogenase-a | SKF | 2.8 |
| 10d12 | CA964196 | lambda 1 | SKF | -2 |
| 22g01 | CA969344 | lambda-recombinase-like protein | LY | 1.4 |
| 19d10 | CA965809 | lamin bpartial | LY | 1.3 |
| 20m01 | CA966310 | latent transforming growth factor beta binding protein 1 | SKF | 2.3 |
| 13o14 | CF662717 | latexin | SKF | 1.6 |
| 14j11 | CA964444 | lck interacting transmembrane adaptor 1 | SKF | -1.5 |
| 08a07 | CA970377 | leucine aminopeptidase 3 | LY | 1.5 |
| 22g07 | CA969350 | leucine rich repeat (in flii) interacting protein 1 | SKF | -1.7 |
| 21k09 | CA967946 | leucine rich repeat containing 2 | LY | 1.5 |
| 11p01 | CF662658 | leucine rich repeat containing 58 | SKF | 2.2 |
| 11f13 | CF662622 | leucine-rich ppr-motif containing | SKF | 1.7 |
| 19l03 | CA969130 | light chainalkaliembryonic | SKF | 2.3 |
| 20d16 | CA966478 | -likemitochondrial | SKF | 2.2 |
| 20j06 | CA966520 | lim and sh3 protein 1 | LY | 1.4 |
| 21g21 | CA967931 | lim domain binding 3 | LY | 1.5 |
| 14n13 | CA964529 | limb and neural patterns | SKF | 1.8 |
| 20a04 | CA965858 | lim-domain binding factor 3 | SKF | 2.2 |
| 22a06 | CA969223 | lim-domain binding factor 3 | LY | 1.5 |
| 20a04 | CA965858 | lim-domain binding factor 3 | LY | 1.3 |
| 06o08 | CF662533 | lipin 1 | LY | 1.6 |
| 11o14 | CF662655 | liver-expressed antimicrobial peptide 2 | LY | 1.5 |
| 19f13 | CA969104 | loc548392 protein | SKF | 2 |
| 05f06 | CF662428 | loc567732 protein | LY | 2.1 |
| 07c13 | CA963997 | loc567732 protein | SKF | 1.8 |
| 09e21 | CF662580 | loc567732 protein | LY | 1.5 |
| 05f06 | CF662428 | loc567732 protein | SKF | 1.5 |
| 05d21 | CA965385 | loc567732 protein | LY | 1.3 |
| 06i18 | CF662499 | loc567732 protein | LY | 1.3 |
| 19e14 | CA965019 | loc568410 protein | SKF | 2.2 |
| 20e01 | CA966265 | loc570315 protein | LY | 1.4 |
| 07n14 | CA968564 | loc795332 protein | SKF | 1.8 |
| 08h19 | CA968711 | loc799918 protein | LY | 1.5 |
| 14k17 | CA964471 | loc799918 protein | SKF | 1.5 |
| 15g14 | CA966746 | lsm7u6 small nuclear rna associated | SKF | 2.1 |
| 12n17 | CA966051 | lsm7u6 small nuclear rna associated | LY | 1.3 |
| 16e13 | CA966368 | ly1 antibody reactive homolog | SKF | 2.5 |
| 07m16 | CA964089 | lymphocyte cytosolic plastin 1 | SKF | 1.5 |
| 18j18 | CA969016 | lyr motif containing 1 | SKF | -1.7 |
| 23g10 | CA968024 | lysosomal-associated protein transmembrane 4 alpha | LY | 1.3 |
| 24k19 | CA969926 | lysozyme g | LY | 1.3 |
| 07d03 | CA968469 | lysyl-trna synthetase | SKF | 2 |
| 07c03 | CA963989 | lysyl-trna synthetase | LY | 1.3 |
| 09g16 | CA964824 | macrophage myristoylated alanine-rich c kinase-like protein | SKF | 1.7 |
| 06m02 | CF662515 | macrophage stimulating 1 (hepatocyte growth factor-like) | LY | 1.3 |
| 20j03 | CA967102 | mads box transcription enhancer factorpolypeptide c (myocyte enhancer factor 2c) | SKF | 1.8 |
| 20j03 | CA967102 | mads box transcription enhancer factorpolypeptide c (myocyte enhancer factor 2c) | LY | 1.3 |
| 07i13 | CF662549 | malate dehydrogenasenad | SKF | 2.1 |
| 14m01 | CA964750 | malate dehydrogenasenad | SKF | 1.8 |
| 14m01 | CA964750 | malate dehydrogenasenad | LY | 1.3 |
| 19i14 | CA965039 | male germ cell-associated kinase | SKF | 2.5 |
| 12k14 | CA970272 | male-specific protein | SKF | 1.9 |
| 10j03 | CA968884 | male-specific protein | SKF | -1.6 |
| 08d10 | CA968644 | malonyl:acp acyltransferase | SKF | 1.7 |
| 08d07 | CA968641 | mannose-binding lectin-associated serine protease-3a | SKF | 1.6 |
| 11n17 | CF662652 | mantle gene 8 | SKF | 2 |
| 23d11 | CF662912 | map microtubule affinity-regulating kinase 1 | SKF | -1.8 |
| 22o11 | CA969512 | map microtubule affinity-regulating kinase 4 | SKF | -2 |
| 02i15 | DY231704 | map-kinase activating death domain | SKF | 2.2 |
| 22c20 | CA969280 | matrix metallopeptidase 13 | SKF | -1.9 |
| 24m12 | CA969960 | mediator complex subunit 22 | SKF | -2 |
| 24m12 | CA969960 | mediator complex subunit 22 | LY | 1.4 |
| 16i02 | CA966159 | meis homeobox 1 | SKF | 1.4 |
| 18n01 | CA969041 | melanocortin 2 receptor | SKF | 1.9 |
| 03h01 | DY231855 | member ras oncogene family | SKF | 2.1 |
| 17n12 | CA966471 | member ras oncogene family | SKF | -1.6 |
| 09n20 | CA964893 | member ras oncogene family | LY | 1.4 |
| 23n19 | CA969682 | member ras oncogene family | LY | 1.4 |
| 21l16 | CA966525 | membranepalmitoylated | SKF | -1.6 |
| 21l16 | CA966525 | membranepalmitoylated | LY | 1.3 |
| 20p16 | CA966563 | meprinalpha (paba peptide hydrolase) | SKF | 1.9 |
| 24b17 | CA969744 | metaxin 2 | LY | 1.4 |
| 24b17 | CA969744 | metaxin 2 | SKF | -2 |
| 20i02 | CA965887 | methionine aminopeptidase 2 | SKF | -1.6 |
| 06i19 | CF662500 | methyl-binding domain protein 3 | LY | 1.8 |
| 09p22 | CA964915 | methylcrotonoyl-coenzyme a carboxylase 2 | SKF | -1.8 |
| 08g10 | CA968689 | methylenetetrahydrofolate dehydrogenase (nadp+ dependent)methenyltetrahydrofolateformyltetrahydrofolate synthetase | SKF | -1.4 |
| 07b23 | CA968465 | methylmalonate-semialdehyde dehydrogenase | SKF | -1.6 |
| 11b05 | CF662609 | methyltransferase domain containing 1 | SKF | -1.5 |
| 12f15 | CA966011 | methyltransferase like 7a | LY | 1.4 |
| 07j21 | CA968534 | mgc82060 protein | LY | 1.5 |
| 22i10 | CA969391 | mgc84259 protein | SKF | -1.6 |
| 22k08 | CA969424 | mhc class i antigen | SKF | -2 |
| 21f13 | CA969180 | mhc class i antigen | SKF | 1.6 |
| 22m08 | CA969467 | mhc class i antigen | LY | 1.4 |
| 22b12 | CA969251 | mhc class ii beta antigen | SKF | -1.5 |
| 21h24 | CF662888 | microsomal glutathione s-transferase 3 | SKF | -1.8 |
| 13g01 | CA967834 | microtubule-associated protein tau | SKF | 1.9 |
| 08n22 | CA968807 | microtubule-associatedrp ebmemberlike | LY | 1.4 |
| 09c02 | CA964776 | mid1 interacting g12-like protein | SKF | 2.5 |
| 10j18 | CA964229 | mid1 interacting g12-like protein | SKF | -1.7 |
| 09c02 | CA964776 | mid1 interacting g12-like protein | LY | 1.3 |
| 05o21 | CA967591 | mid1-interacting protein 1 | LY | 1.3 |
| 08l01 | CA965449 | middle subunit | SKF | 2.5 |
| 03k10 | FG392604 | midkine-related growth factor b | SKF | -1.5 |
| 05k03 | CA967552 | minor histocompatibility antigen h13 | SKF | 1.6 |
| 22k17 | CA969433 | minor histocompatibility antigen ha-1 | LY | 1.4 |
| 22k17 | CA969433 | minor histocompatibility antigen ha-1 | SKF | -1.8 |
| 06m20 | CF662521 | mitochondrial atph+ transporting f1 complex beta subunit | LY | 1.6 |
| 17j18 | CA966516 | mitochondrial atph+ transporting f1 complex beta subunit | SKF | -1.6 |
| 17j18 | CA966516 | mitochondrial atph+ transporting f1 complex beta subunit | LY | 1.3 |
| 12n01 | CA966046 | mitochondrial ribosomal protein l19 | SKF | 1.5 |
| 24n13 | CF662920 | mitochondrial ribosomal protein l20 | LY | 1.6 |
| 19p16 | CA967272 | mitochondrial ribosomal protein l20 | SKF | 2 |
| 19e17 | CA966188 | mitochondrial ribosomal protein l24 | SKF | 1.4 |
| 24p14 | CA970018 | mitochondrial ribosomal protein s34 | LY | 1.7 |
| 11b01 | CA966410 | mitochondrial ubiquitin ligase activator of nfkb 1 | SKF | 2.6 |
| 19a06 | CA964994 | mitogen-activated protein kinase 11 | LY | 1.4 |
| 19d20 | CA965813 | mitogen-activated protein kinase 3 | SKF | 1.5 |
| 19d20 | CA965813 | mitogen-activated protein kinase 3 | LY | 1.3 |
| 24b02 | CA969730 | mitogen-activated protein kinase kinase 1 | LY | 1.4 |
| 11j11 | CF662634 | mitogen-activated protein kinase kinase kinase 7 interacting protein 3 | SKF | -1.7 |
| 19o07 | CA966235 | moesin | SKF | -1.5 |
| 03l05 | DY231946 | monoamine oxidase b | SKF | 1.5 |
| 12p13 | CA966058 | m-phase phosphoprotein 6 | SKF | 2.1 |
| 12o08 | CA970287 | mps one binder kinase activator-like 1a | LY | 1.5 |
| 21n16 | CA966527 | mps one binder kinase activator-like 1b | SKF | 1.9 |
| 21c15 | CA967910 | myelin basic protein | SKF | 2 |
| 22c11 | CA969271 | myelin basic protein | LY | 1.3 |
| 23m15 | CA968088 | myoglobin | LY | 1.4 |
| 21a20 | CA966565 | myo-inositol 1-phosphate synthase a1 | SKF | 1.5 |
| 04p11 | FG392940 | myo-inositol 1-phosphate synthase a1 | LY | 1.4 |
| 08i11 | CA968720 | myo-inositol oxygenase | LY | 1.4 |
| 21o18 | CA966528 | myosin binding protein h | LY | 1.7 |
| 21c13 | CA967909 | myosin binding proteincardiac | SKF | 2.1 |
| 16i16 | CA966162 | myosin binding proteinfast type | SKF | 1.7 |
| 21m18 | CF662898 | myosin binding proteinfast-type | LY | 1.5 |
| 14h04 | CA964400 | myosin heavy chain | SKF | 1.5 |
| 14g03 | CA964384 | myosin light chain 2 | SKF | 1.9 |
| 14i08 | CA964420 | myosin light chain 2 | LY | 1.5 |
| 14f11 | CA964371 | myosin light chain 2 | SKF | -1.5 |
| 14a05 | CA964274 | myosin light chain 3 | LY | 1.4 |
| 13c21 | CA967824 | myosin light chain 3 | SKF | -1.5 |
| 13o03 | CA967872 | myosin regulatory light chainsmooth muscle isoform | SKF | 1.7 |
| 11m13 | CA965975 | myosin viia | LY | 1.4 |
| 08h14 | CA968706 | myotrophin | SKF | 2.2 |
| 07o08 | CF662555 | myst histone acetyltransferase 2 | LY | 1.4 |
| 04a01 | FG392680 | na+ k+alpha 1b polypeptide | SKF | 2.2 |
| 24a05 | CA969713 | na+ k+alphapolypeptide | LY | 1.4 |
| 11k05 | CA965964 | n-acylsphingosine amidohydrolase 3-like | SKF | 1.5 |
| 19k18 | CA965052 | nadh dehydrogenasefe-s protein 2 | SKF | 1.6 |
| 15g02 | CA965615 | nadh dehydrogenaseflavoprotein24kda | SKF | 2.5 |
| 22i14 | CA969395 | nadhquinone 2 | SKF | -1.5 |
| 12m01 | CA970276 | nascent polypeptide-associated complex subunit alpha | SKF | 2 |
| 21n01 | CF662899 | nattectin precursor | LY | 1.6 |
| 24b19 | CA969746 | nck adaptor protein 2 | SKF | -1.5 |
| 20a14 | CA965863 | ndrg family member 3 | SKF | 1.9 |
| 14g05 | CA964386 | necap endocytosis associated 1 | SKF | 1.4 |
| 19j16 | CA965835 | nedd4 family interacting protein 1 | SKF | 2 |
| 24e17 | CA969801 | nedd4 family interacting protein 1 | LY | 1.4 |
| 19l18 | CA965844 | negative elongation factor d | SKF | 1.5 |
| 03i12 | FG392591 | nel-like 2 | SKF | -1.7 |
| 04j18 | FG392958 | n-ethylmaleimide-sensitive factor | SKF | 1.4 |
| 24a13 | CA966813 | netrin 4 | SKF | -1.7 |
| 19c06 | CA965005 | neurexin 2 isoform alpha-1 precursor | LY | 1.3 |
| 22e08 | CA969310 | neuritin 1 | SKF | -1.9 |
| 02f13 | DY231623 | neuronal nitric oxide synthase | SKF | 1.7 |
| 08h16 | CA968708 | neuropilin 1 | SKF | 1.7 |
| 22p17 | CA969537 | neuroserpin precursor | SKF | -1.6 |
| 09m15 | CA964168 | neurotoxin c59 ly-6-like protein | SKF | 1.4 |
| 09m15 | CA964168 | neurotoxin c59 ly-6-like protein | LY | 1.3 |
| 19f03 | CA969100 | n-formyl peptide receptor | SKF | 1.5 |
| 19f03 | CA969100 | n-formyl peptide receptor | LY | 1.3 |
| 19j17 | CA969126 | nhl repeat-containing protein 3 precursor | SKF | 1.6 |
| 22h12 | CA969375 | nhs-like 2 | SKF | -1.6 |
| 24e21 | CA969805 | nicotinamide riboside kinase 2 | LY | 1.4 |
| 10e19 | CA967640 | nidogen 1 | SKF | -1.6 |
| 19p09 | CA969150 | nin one binding protein | LY | 1.4 |
| 16k15 | CF662774 | nlrcard domain containing 3 | SKF | 1.8 |
| 12g14 | CA966718 | nmda receptor-regulated gene 1 | SKF | 2.2 |
| 22e20 | CA969320 | N-methyl d-aspartate-associated protein 1 (glutamate binding) | LY | 1.6 |
| 12o15 | CA967803 | n-myc downstream regulated gene 1 | SKF | 1.8 |
| 18c18 | CA964613 | nol1 nop2 sun domainmember 2 | SKF | -1.5 |
| 22k20 | CA969436 | non-erythrocytic 1 (alpha-fodrin) | LY | 1.5 |
| 07d01 | CA968467 | non-metastatic cellsprotein expressed in | SKF | 1.9 |
| 07m24 | CA964093 | non-metastatic cellsprotein expressed in | SKF | -1.7 |
| 15h18 | CA965629 | non-smc elementmms21 homolog | LY | 1.3 |
| 20h23 | CF662865 | nonspecific cytotoxic cell receptor protein 1 | SKF | 1.6 |
| 21o07 | CA967275 | nonspecific cytotoxic cell receptor protein 1 | LY | 1.4 |
| 12a15 | CA967735 | nop16 nucleolar protein homolog | SKF | 1.9 |
| 12a15 | CA967735 | nop16 nucleolar protein homolog | LY | 1.4 |
| 15p05 | CA965721 | novel 2og-feoxygenase superfamily protein | SKF | 1.7 |
| 06f19 | CA965425 | novel carboxylesterase domain containing protein | LY | 1.5 |
| 20c12 | CA965870 | novel connexin family protein | SKF | -1.7 |
| 13o04 | CA966362 | novel laminin egf-like (domains iii and v) containing protein | SKF | 1.9 |
| 06e08 | CF662478 | novel protein | LY | 1.7 |
| 06b03 | CF662464 | novel protein | LY | 1.6 |
| 08e03 | CA970383 | novel protein | SKF | 2.1 |
| 18m13 | CA964713 | novel protein | SKF | 2.2 |
| 08k03 | CA970400 | novel protein | SKF | 2.2 |
| 11h02 | CA966098 | novel protein | SKF | 2.2 |
| 14i13 | CA964425 | novel protein | SKF | 2 |
| 24h01 | CA969847 | novel protein | SKF | 2 |
| 09k18 | CA964861 | novel protein | SKF | -1.6 |
| 02a15 | DY231592 | novel protein | SKF | 1.7 |
| 14p02 | CA964558 | novel protein | SKF | 1.7 |
| 12b19 | CA965990 | novel protein | SKF | 1.7 |
| 11b17 | CA966412 | novel protein | SKF | 1.6 |
| 14m17 | CA964512 | novel protein | SKF | -1.9 |
| 14g07 | CA964746 | novel protein | LY | 1.5 |
| 12n10 | CA965470 | novel protein | SKF | 1.5 |
| 19b18 | CA965802 | novel protein | SKF | 1.5 |
| 21l19 | CA969207 | novel protein | SKF | -1.7 |
| 20a16 | CA965864 | novel protein | LY | 1.5 |
| 06d20 | CF662475 | novel protein | SKF | -1.8 |
| 24d08 | CA969777 | novel protein | SKF | -1.8 |
| 08o01 | CA968809 | novel protein | SKF | 1.5 |
| 17m02 | CF662801 | novel protein | SKF | 1.5 |
| 08g02 | CA968683 | novel protein | LY | 1.4 |
| 12b19 | CA965990 | novel protein | LY | 1.4 |
| 24d08 | CA969777 | novel protein | LY | 1.5 |
| 11d07 | CF662614 | novel protein | SKF | -1.5 |
| 18g02 | CA964640 | novel protein | SKF | 1.4 |
| 14i03 | CA964748 | novel protein (zgc:103442) | SKF | 1.6 |
| 15i06 | CA965636 | novel protein (zgc:136439) | SKF | 1.6 |
| 11g05 | CA965945 | novel protein (zgc:158868) | SKF | 1.7 |
| 14l13 | CA964490 | novel protein containing an inosine-uridine preferring nucleoside hydrolase domain | SKF | 2.1 |
| 09n04 | CA964886 | novel protein containing multiple sushi domains (scr repeat) | SKF | 1.8 |
| 08n05 | CA968793 | novel protein containing multiple sushi domains (scr repeat) | SKF | 1.6 |
| 08c06 | CF662560 | novel protein containing multiple sushi domains (scr repeat) | LY | 1.4 |
| 09f06 | CA964807 | novel protein containing trypsin domains | SKF | 1.5 |
| 15h10 | CA965626 | novel proteinhairy-related | LY | 1.5 |
| 19g15 | CA966197 | novel proteinhuman titin | SKF | 1.5 |
| 15b13 | CA965552 | novel proteinlim domain only 3 (rhombotin-like 2) (zgc:110149) | SKF | 2 |
| 24f05 | CA969812 | novel proteinvertebrate activating transcription factor 5 | SKF | -1.5 |
| 07d10 | CA968476 | novel proteinvertebrate ankyrin repeat domain 15 | LY | 1.3 |
| 11m15 | CA965976 | novel proteinvertebrate apolipoprotein b (including agantigen) | SKF | 1.8 |
| 07g14 | CA964033 | novel proteinvertebrate apolipoprotein b (including agantigen) | SKF | 1.7 |
| 07g14 | CA964033 | novel proteinvertebrate apolipoprotein b (including agantigen) | LY | 1.3 |
| 08l03 | CA968763 | novel proteinvertebrate complement component 3 | SKF | 2.9 |
| 19b16 | CA965801 | novel proteinvertebrate dab2 interacting protein | SKF | 2.1 |
| 22o08 | CA969510 | novel proteinvertebrate galectins | SKF | -2 |
| 09g14 | CA964823 | novel proteinvertebrate interferon-induced protein 44 | SKF | 1.8 |
| 12o21 | CA967806 | novel proteinvertebrate interferon-induced protein 44 | SKF | 1.4 |
| 14n05 | CA964523 | novel proteinvertebrate muscle derived protein | SKF | 1.6 |
| 08n20 | CA968805 | novel proteinvertebrate quiescin q6 sulfhydryl oxidase 1 | LY | 1.5 |
| 24e24 | CA969807 | novel proteinvertebrate sarcospan (kras oncogene-associated gene) | SKF | -1.7 |
| 19h03 | CA969110 | novel proteinvertebrate synaptotagmin ii | SKF | 2.6 |
| 19h03 | CA969110 | novel proteinvertebrate synaptotagmin ii | LY | 1.3 |
| 07a06 | CA970428 | novel proteinvitellogenin 1 | SKF | 1.7 |
| 11e15 | CA965939 | novel sulfotransferase family protein | SKF | 1.9 |
| 16f16 | CF662761 | nuclear factor i a | SKF | 2.1 |
| 08m20 | CA966758 | nuclear receptor subfamilygroupmember 1 | SKF | 1.5 |
| 08m20 | CA966758 | nuclear receptor subfamilygroupmember 1 | LY | 1.4 |
| 12a01 | CA967729 | nuclear receptor subfamilygroupmember 2 | SKF | 1.9 |
| 19e01 | CA966183 | nuclear receptor subfamilygroupmember 2 | SKF | 2.1 |
| 11g08 | CA966336 | nuclear transcription factorbeta | SKF | -1.5 |
| 17e21 | CA964945 | nuclear transcriptionx-box binding 1 | LY | 1.4 |
| 24b11 | CA969739 | nuclearinterleukin 3 regulated | LY | 1.3 |
| 18h10 | CA968990 | nucleobindin 2a | SKF | -1.8 |
| 08b23 | CA968620 | nucleoporin 155 | SKF | 1.5 |
| 10f04 | CA964203 | nucleoside diphosphate kinase | SKF | -1.6 |
| 12i01 | CA967769 | nucleoside phosphorylase | SKF | 1.6 |
| 18f13 | CA968974 | odd oz\ ten-m homolog 2 | SKF | 1.5 |
| 23b09 | CA969549 | oligophrenin 1 | LY | 1.6 |
| 12o10 | CA970288 | o-linked n-acetylglucosaminetransferase (udp-n-acetylglucosamine:polypeptide-n-acetylglucosaminyl transferase) | LY | 1.3 |
| 23p22 | CA969706 | orf2-encoded protein | LY | 1.7 |
| 23m09 | CA968082 | orf2-encoded protein | LY | 1.5 |
| 08c18 | CF662561 | orm1-like 1 | SKF | 1.6 |
| 09b06 | CA964767 | ornithine aminotransferase | LY | 1.4 |
| 09a02 | CA964755 | ovary-specific c1q-like factor | SKF | 2.5 |
| 09a02 | CA964755 | ovary-specific c1q-like factor | LY | 1.3 |
| 20f20 | CF662859 | overexpressed breast tumor protein homolog | LY | 1.5 |
| 09d01 | CF662575 | oxoglutarate (alpha-ketoglutarate) dehydrogenase | SKF | 2.2 |
| 09d01 | CF662575 | oxoglutarate (alpha-ketoglutarate) dehydrogenase | LY | 1.3 |
| 23a20 | CA967277 | oxysterol-bindingprotein 10 | LY | 1.3 |
| 11b14 | CA966071 | p53-associated parkin-like cytoplasmic protein | SKF | 1.9 |
| 19m09 | CA966225 | palmitoyl-protein thioesterase 1 | LY | 1.5 |
| 24f10 | CA969816 | parvalbumin | LY | 1.6 |
| 23p21 | CA969705 | parvalbumin | LY | 1.5 |
| 24e10 | CA969796 | parvalbumin | LY | 1.4 |
| 24m24 | CA969969 | parvalbumin | SKF | -1.6 |
| 20p03 | CF662876 | pdz and lim domain 5 | LY | 1.4 |
| 23b02 | CA969542 | pdz domain containing 11 | SKF | 1.4 |
| 15k16 | CA965666 | pdz domain containing 3 | SKF | 1.5 |
| 22e03 | CA969306 | pdz domain-containing guanine nucleotide exchange factor i isoform 2 | LY | 1.3 |
| 10o12 | CA967718 | peptidoglycan recognition protein ii | SKF | -1.5 |
| 10k12 | CA967686 | peptidylprolyl isomerase b (cyclophilin b) | SKF | -1.6 |
| 17j19 | CA965773 | peroxiredoxin 6 | LY | 1.5 |
| 08l19 | CA968775 | peroxisomal short-chain alcohol dehydrogenase | LY | 1.5 |
| 22l17 | CA969455 | pest proteolytic signal-containing nuclear protein | SKF | -1.7 |
| 23o02 | CA968097 | phf23a protein | SKF | -1.6 |
| 15e23 | CA966723 | phosducin-like 3 | SKF | -1.6 |
| 06p19 | CA967117 | phosphate cytidylyltransferaseethanolamine | LY | 1.5 |
| 04k14 | FG392966 | phosphatidylinositol binding clathrin assembly protein | SKF | 1.6 |
| 21k05 | CF662895 | phosphatidylinositol glycan anchorclass s | SKF | 1.7 |
| 20c02 | CA965868 | phosphatidylinositol transfercytoplasmicpartial | SKF | 1.6 |
| 07p14 | CA968584 | phosphoenolpyruvate carboxykinase | SKF | 1.7 |
| 19f02 | CA965816 | phosphofructokinase | SKF | 1.8 |
| 19f02 | CA965816 | phosphofructokinase | LY | 1.4 |
| 18j15 | CA969013 | phosphoglucomutase 1 | LY | 1.3 |
| 21n17 | CA969214 | phosphoglucose isomerase | SKF | -1.9 |
| 07h14 | CF662548 | phosphoglycerate mutase 1 | SKF | 1.7 |
| 06d21 | CA965418 | phosphoglycerate mutase 1 | LY | 1.6 |
| 06o19 | CF662537 | phosphoglycerate mutase 1 | SKF | -1.5 |
| 08k13 | CA970403 | phosphohistidine phosphatase 1 | SKF | 1.9 |
| 09m03 | CA964162 | phospholemman precursor | SKF | 1.5 |
| 09m03 | CA964162 | phospholemman precursor | LY | 1.3 |
| 23i20 | CA968051 | phospholipasegroup xiia | SKF | -1.8 |
| 20o06 | CA965907 | phospholipid hydroperoxide glutathione peroxidase | SKF | 1.6 |
| 23d21 | CA969582 | phospholipid hydroperoxide glutathione peroxidase | LY | 1.3 |
| 19n18 | CA967036 | phosphoribosyl pyrophosphate synthetase 1 | SKF | 1.5 |
| 19k11 | CA966215 | phosphorylase kinase alpha 1 | LY | 1.7 |
| 21h13 | CA969189 | phosphorylase kinase gamma subunit 1 | LY | 1.5 |
| 08b11 | CA968611 | photosystem i assembly | LY | 1.3 |
| 14g15 | CA964392 | pi-class glutathione s-transferase | SKF | -1.8 |
| 20e13 | CA966271 | pkm2a protein | SKF | 1.6 |
| 08c01 | CA968621 | pl-5283 protein | SKF | 2 |
| 19d13 | CA969093 | plac8-like 1 | SKF | 3.5 |
| 24b24 | CA969750 | plasma membrane proteolipid | SKF | -1.6 |
| 12l11 | CA966039 | plasma retinol-binding protein 1 | SKF | -1.5 |
| 11g13 | CA965948 | plasminogen | SKF | 2.7 |
| 24d20 | CA969785 | plasticity related gene 3 | LY | 1.4 |
| 11e03 | CA965934 | platelet-activating factorisoformbeta subunit 30kda | SKF | 1.5 |
| 12l03 | CA966036 | platelet-activating factorisoformgamma subunit 29kda | SKF | 1.7 |
| 12l03 | CA966036 | platelet-activating factorisoformgamma subunit 29kda | LY | 1.4 |
| 14k15 | CA964469 | platelet-derived growth factor alpha polypeptide | LY | 1.3 |
| 22e05 | CA969307 | platelet-derived growth factor receptor beta | LY | 1.3 |
| 04d18 | FG392801 | pleckstrin homology domainfamily j member 1 | SKF | 1.5 |
| 19a10 | CA966999 | polo-like kinase 2 | LY | 1.3 |
| 22k19 | CA969435 | poly a bindingcytoplasmic 1 b | LY | 1.4 |
| 20m02 | CF662871 | polybindingcytoplasmic 1 | SKF | 2.9 |
| 24a23 | CA969728 | polybindingnuclear 1 | LY | 1.6 |
| 12a19 | CA967737 | polymeraseii (dna directed) polypeptide h | SKF | -1.7 |
| 22m20 | CA969478 | poly-u binding splicing factor 60kda | LY | 1.6 |
| 03j14 | FG392639 | potassium channel tetramerisation domain containing 4 | SKF | 2 |
| 17n22 | CF662806 | potassium channel tetramerisation domain containing 6 | SKF | -1.7 |
| 24b01 | CA969729 | ppp1r13b protein | LY | 1.4 |
| 12e11 | CA967752 | pre-mrna processing factor 8 | SKF | 1.5 |
| 08j20 | CA968740 | preproelastase i | LY | 1.4 |
| 05e19 | CA967516 | proactivator polypeptide precursor | SKF | -2 |
| 10b19 | CA968846 | procollagen c-endopeptidase enhancer | SKF | -1.7 |
| 14k18 | CA964472 | profilin 1 | SKF | 1.4 |
| 12a03 | CA967730 | progesterone receptor membrane component 1 | SKF | 2.2 |
| 10e13 | CA967636 | progesterone receptor membrane component 1 | SKF | -1.5 |
| 10n17 | CA968910 | programmed cell death 10 | LY | 1.3 |
| 24f07 | CA969814 | programmed cell death 6 | LY | 1.3 |
| 22e11 | CA969313 | prolactin releasing hormone receptor | LY | 1.5 |
| 24a19 | CA969725 | proline rich 6 | LY | 1.3 |
| 22c10 | CA969270 | prosaposin | LY | 1.6 |
| 08o11 | CA970419 | prostaglandin d synthase | LY | 1.5 |
| 14c02 | CA964306 | prostaglandin e synthase 3 | SKF | 2 |
| 12o01 | CA967797 | prostaglandin h2 d-isomerase | SKF | 2.1 |
| 15a04 | CA965542 | prostaglandin-endoperoxide synthase 2 (prostaglandin g h synthase and cyclooxygenase) | SKF | 1.6 |
| 03l03 | FG392607 | protachykinin 1 precursor | SKF | 2.4 |
| 15p14 | CA965729 | proteasome 26s atpase subunit 3 | SKF | 2.2 |
| 22c01 | CA969261 | proteasome 26s non-atpase subunit 11 | LY | 1.4 |
| 14j21 | CA964453 | proteasome 26s non-atpase subunit 4 | SKF | -1.9 |
| 05c08 | CA967489 | proteasome 26s non-atpase subunit 8 | LY | 1.3 |
| 19k09 | CA966214 | proteasome activator subunit 2 | LY | 1.5 |
| 14d07 | CA964327 | proteasome subunit alpha type-2 | SKF | -1.5 |
| 13g13 | CA967839 | proteasome subunit alpha type-3 | SKF | 1.4 |
| 06h06 | CA965429 | proteasome subunit alpha type-7 | LY | 1.5 |
| 23k10 | CA968062 | proteasome subunit beta type-6 precursor | LY | 1.4 |
| 16j16 | CF662772 | proteasome subunit beta type-9 precursor | SKF | 1.5 |
| 16j14 | CA966407 | proteasome26s1 | SKF | 1.4 |
| 15f22 | CA965612 | proteasome26s1 | LY | 1.3 |
| 16a14 | CA967223 | proteasome26s5 | SKF | 2.7 |
| 13f16 | CA966430 | proteasome26snon-10 | SKF | 1.4 |
| 22p03 | CA969527 | proteasome26snon-4 | SKF | -1.6 |
| 12b01 | CA965983 | proteasomealpha type 5 | SKF | 2 |
| 12b01 | CA965983 | proteasomealpha type 5 | LY | 1.3 |
| 11i10 | CA966384 | proteasomealpha1 | LY | 1.3 |
| 22e12 | CA969314 | proteasomebeta type 5 | SKF | -2 |
| 22i12 | CA969393 | protein | SKF | -1.8 |
| 24m09 | CA969958 | protein | LY | 1.4 |
| 11o07 | CA965979 | protein | LY | 1.3 |
| 12n14 | CA966427 | protein disulfide isomerase familymember 3 | SKF | 2.5 |
| 12n14 | CA966427 | protein disulfide isomerase familymember 3 | LY | 1.3 |
| 21j01 | CF662891 | protein kinase c and casein kinase substrate in neurons 2 | SKF | 1.7 |
| 11m21 | CA965978 | protein phosphatase 1 regulatory subunit 16a (myosin phosphatase targeting subunit 3) | LY | 1.3 |
| 17d19 | CF662788 | protein phosphatase 1k (pp2c domain containing) | LY | 1.5 |
| 17d15 | CA967266 | protein phosphatasecatalyticgamma isoform | SKF | -1.5 |
| 12g01 | CA967759 | protein phosphataseregulatory subunitdelta isoform | SKF | 1.6 |
| 09h18 | CA964834 | protein phosphataseregulatorysubunit 3c | SKF | 1.9 |
| 17h01 | CA965759 | protein tyrosine phosphatase typemember 1 | SKF | 1.5 |
| 24h18 | CA969863 | protein tyrosine phosphatase-member a | SKF | -1.7 |
| 16g02 | CA966157 | protein tyrosinereceptorb | SKF | 3.5 |
| 12i24 | CA970266 | protein tyrosinereceptor-f interactingbinding proteinisoform cra_f | SKF | -1.7 |
| 04e14 | FG392809 | protein tyrosinereceptorn polypeptide 2 | SKF | 1.9 |
| 07l24 | CA965514 | proteinamp-alpha 2 catalytic subunit | SKF | -1.5 |
| 22d19 | CA969300 | protocadherin 1 gamma 31 | LY | 1.6 |
| 19p14 | CF662842 | prp3 pre-mrna processing factor 3 homolog | LY | 1.5 |
| 09a21 | CA964110 | prp40 pre-mrna processing factor 40 homolog a | LY | 1.4 |
| 09a21 | CA964110 | prp40 pre-mrna processing factor 40 homolog a | SKF | 1.4 |
| 16k13 | CF662773 | prp6 pre-mrna splicing factor 6 homolog | SKF | 2.1 |
| 22k03 | CA969422 | psmd7 protein | SKF | -2 |
| 23o07 | CA967043 | purinergic receptorligand-gated ion5 | LY | 1.5 |
| 23o07 | CA967043 | purinergic receptorligand-gated ion5 | SKF | -1.7 |
| 08h23 | CA968714 | pyruvate dehydrogenasebeta | LY | 1.3 |
| 20f04 | CF662853 | pyruvate kinase | SKF | 1.9 |
| 17o03 | CA964987 | pyruvate kinase | LY | 1.3 |
| 05k01 | CA967550 | pyruvateliver and rbc | SKF | 1.9 |
| 09g11 | CA964133 | quiescin q6-like 1 | LY | 1.3 |
| 24h10 | CA969855 | r3h domain containing 2 | LY | 1.5 |
| 24h10 | CA969855 | r3h domain containing 2 | SKF | -1.7 |
| 07e04 | CA964009 | rab acceptor 1 | SKF | 1.5 |
| 18c02 | CA964598 | rab gtpase binding effector protein 2 | LY | 1.3 |
| 18e20 | CA964634 | rab6 interacting protein 1 | SKF | -1.7 |
| 09e12 | CA964801 | ran binding protein 1 | LY | 1.3 |
| 08f08 | CA968670 | ran gtpase activating protein 1 | LY | 1.3 |
| 14k10 | CA964464 | ras homolog genemember t1b | LY | 1.3 |
| 24p13 | CA970017 | ras-gtpase-activating protein sh3-domain-binding protein | LY | 1.5 |
| 18c09 | CA964604 | ras-related c3 botulinum toxin substrate 1 (rhosmall gtp binding protein rac1) | LY | 1.6 |
| 18c09 | CA964604 | ras-related c3 botulinum toxin substrate 1 (rhosmall gtp binding protein rac1) | SKF | -1.7 |
| 15p17 | CA965731 | Ras-related protein Rap-1b precursor | SKF | -1.6 |
| 14f03 | CA964364 | receptor accessory protein 5 | SKF | 1.5 |
| 14f03 | CA964364 | receptor accessory protein 5 | LY | 1.3 |
| 24j19 | CA969907 | receptor tyrosine kinase | LY | 1.3 |
| 22a07 | CA969224 | RecQ protein-like DNA helicase Q1-like | SKF | -1.6 |
| 06b19 | CA965411 | regucalcin | LY | 1.3 |
| 24j20 | CA969908 | regulating synaptic membrane exocytosis 2 | SKF | -1.5 |
| 24i20 | CA969886 | regulator of g-protein signaling 3 | LY | 1.3 |
| 16a08 | CA967222 | regulatory factor x-associated ankyrin-containing protein | LY | 1.4 |
| 17a21 | CA964924 | remodeling and spacing factor 1 | LY | 1.5 |
| 08e02 | CA966755 | replication factor c (activator 1) 3 | SKF | 2.3 |
| 22b23 | CA969259 | response gene to complement 32 | SKF | -2 |
| 16e08 | CA966156 | response gene to complement 32 | LY | 1.4 |
| 03k04 | DY231940 | reticulon 4 | SKF | 1.8 |
| 12b17 | CA965989 | retinoblastoma binding protein 4 | SKF | 1.5 |
| 12b17 | CA965989 | retinoblastoma binding protein 4 | LY | 1.3 |
| 08n01 | CA968790 | retinoblastoma binding protein 5 | SKF | 3 |
| 20a15 | CA966249 | retinoic acid induced 17 | LY | 1.3 |
| 15j09 | CA965649 | retinoic acid receptor responder protein 3 | LY | 1.3 |
| 12b06 | CA966421 | retinol binding proteincellular | SKF | 1.7 |
| 08d01 | CA968636 | retinol dehydrogenase 10 | SKF | 1.4 |
| 08d01 | CA968636 | retinol dehydrogenase 10 | LY | 1.4 |
| 10b11 | CA968843 | reverse transcriptase ribonuclease h methyltransferase | SKF | -1.5 |
| 12a04 | CA970217 | rgm domainmember b | LY | 1.4 |
| 12a04 | CA970217 | rgm domainmember b | SKF | 1.5 |
| 13a17 | CA967815 | rh type b glycoprotein | SKF | -1.5 |
| 13f04 | CA966429 | rhamnose binding lectin | SKF | -1.7 |
| 13c11 | CF662690 | rhamnose binding lectin | LY | 1.3 |
| 22b08 | CA969248 | rho guanine nucleotide exchange factor 4 | SKF | -1.6 |
| 12n13 | CA966049 | ribokinase | SKF | 2.2 |
| 08o17 | CA970422 | ribonuclease like 2 | SKF | -1.7 |
| 11m18 | CF662647 | ribonuclease like 2 | SKF | 1.5 |
| 21j11 | CA969197 | ribonuclease p mrp 30kda subunit | LY | 1.7 |
| 16k02 | CA966163 | ribophorin i | SKF | 1.6 |
| 08h21 | CA968713 | ribophorin ii | SKF | 1.5 |
| 19h20 | CA965828 | ribose 5-phosphate isomerase a (ribose 5-phosphate epimerase) | SKF | 1.4 |
| 08n06 | CA968794 | ribosomal protein | SKF | 1.7 |
| 09b10 | CA964769 | ribosomal protein l10 | SKF | 1.4 |
| 11c13 | CA965929 | ribosomal protein l10a | SKF | 2.8 |
| 12c03 | CA970229 | ribosomal protein l10a | SKF | 1.6 |
| 08n21 | CA968806 | ribosomal protein l10a | LY | 1.4 |
| 22g21 | CA969362 | ribosomal protein l13 | SKF | -1.7 |
| 08c10 | CA968627 | ribosomal protein l13 | LY | 1.3 |
| 08b14 | CA968613 | ribosomal protein l13a | SKF | 1.7 |
| 09e13 | CA964126 | ribosomal protein l18 | SKF | 2.2 |
| 11p21 | CF662662 | ribosomal protein l23 | SKF | 1.7 |
| 08p04 | CA968823 | ribosomal protein l26 | SKF | 1.6 |
| 08p04 | CA968823 | ribosomal protein l26 | LY | 1.4 |
| 12d13 | CA965998 | ribosomal protein l27a | SKF | 1.6 |
| 19c22 | CA965012 | ribosomal protein l27a | LY | 1.4 |
| 08b15 | CA968614 | ribosomal protein l30 | SKF | 3 |
| 12a12 | CA970221 | ribosomal protein l30 | LY | 1.3 |
| 08p08 | CA968827 | ribosomal protein l32 | SKF | 1.5 |
| 08p03 | CF662570 | ribosomal protein l36 | SKF | 2.5 |
| 08g08 | CA968687 | ribosomal protein l36a | SKF | 1.5 |
| 08h04 | CA968699 | ribosomal protein l37 | SKF | 1.8 |
| 08j16 | CA968738 | ribosomal protein l5 | LY | 1.4 |
| 10e04 | CA967630 | ribosomal protein l5 | SKF | -1.5 |
| 14l05 | CA964483 | ribosomal protein l7a | SKF | 1.5 |
| 09e07 | CA964123 | ribosomal protein l8 | LY | 1.4 |
| 14c04 | CA964307 | ribosomal protein s20 | SKF | 1.7 |
| 09e15 | CA964127 | ribosomal protein s21 | SKF | 2 |
| 08e14 | CA968660 | ribosomal protein s27 | SKF | 1.7 |
| 11p06 | CA966138 | ribosomal protein s5 | SKF | 1.8 |
| 12f05 | CA966006 | ribosomal protein s6 | SKF | 1.7 |
| 22l01 | CA969441 | ribosomal protein s6polypeptide 2 | SKF | -1.6 |
| 09a06 | CA964757 | ribosomal protein s8 | SKF | 1.6 |
| 14m13 | CA964508 | ribosomal protein sa | SKF | 1.7 |
| 22e14 | CA969316 | ribosomal protein sa | SKF | -1.6 |
| 09j03 | CF662588 | ribosomallarge p2 | SKF | 2.5 |
| 09j03 | CF662588 | ribosomallarge p2 | LY | 1.4 |
| 22e01 | CA969305 | riken cdna 2400001e08 gene | LY | 1.3 |
| 22l06 | CA969446 | riken cdna 2610030h06 gene | LY | 1.4 |
| 08p06 | CA968825 | ring finger and chy zinc finger domain containing 1 | SKF | 2.1 |
| 24h03 | CA969849 | ring finger protein 185 | SKF | -1.7 |
| 24h03 | CA969849 | ring finger protein 185 | LY | 1.3 |
| 17g11 | CA964952 | ring finger protein 29 | LY | 1.5 |
| 06p20 | CA967019 | ripply2 protein | LY | 1.3 |
| 24d23 | CA966814 | rna binding motif protein 10 | SKF | -1.5 |
| 10j14 | CA964227 | rna binding motif protein 34 | SKF | -1.5 |
| 19d03 | CA969089 | ryanodine receptor 3 | SKF | 1.5 |
| 02i14 | DY231703 | s100 calcium binding protein b | SKF | 1.6 |
| 20k19 | CA966307 | s100 calcium binding protein b | LY | 1.5 |
| 19g17 | CA966198 | s100 calcium binding protein v2-like | LY | 1.3 |
| 10c11 | CA967617 | sar1a gene homolog 2 | SKF | -1.5 |
| 19d21 | CA969097 | sar1a gene homolog 2 | SKF | 1.4 |
| 14b02 | CA964285 | sarcoendoplasmic reticulum calcium atpase | SKF | 1.9 |
| 14b02 | CA964285 | sarcoendoplasmic reticulum calcium atpase | LY | 1.5 |
| 22e06 | CA969308 | sdcg3_danre serologically defined colon cancer antigen 3 homolog | LY | 1.3 |
| 08a20 | CA968598 | sec14-like 2 | LY | 1.4 |
| 16m02 | CA966164 | sec23 interacting protein | SKF | 1.5 |
| 03d05 | FG392558 | secretogranin ii | SKF | 1.7 |
| 17o09 | CA964989 | secretogranin ii | SKF | -1.7 |
| 22i03 | CA969386 | selenophosphate synthetase 2 | SKF | -1.6 |
| 05j09 | CF662443 | selenoprotein p precursor | LY | 1.5 |
| 23d13 | CA969575 | semaphorin 7a | LY | 1.7 |
| 23d13 | CA969575 | semaphorin 7a | SKF | -1.6 |
| 22j03 | CA969405 | sept2 protein | LY | 1.4 |
| 20l16 |  | septin 7b | SKF | 2.4 |
| 08b12 | CF662558 | serglycin precursor | LY | 1.4 |
| 09l22 | CF662592 | serine peptidasekazal type 2 (acrosin-trypsin inhibitor) | LY | 1.3 |
| 02i01 | DY231646 | serine proteinaseclademember 1 | SKF | 1.7 |
| 10k23 | CA967691 | serinelong chain base subunit 2 | SKF | -1.9 |
| 07c21 | CA964004 | serpina1 protein | LY | 1.6 |
| 07b03 | CA968446 | serpina1 protein | LY | 1.5 |
| 05p21 | CA965402 | serpina1 protein | SKF | -1.6 |
| 07b03 | CA968446 | serpina1 protein | SKF | 1.5 |
| 05i09 | CF662440 | serpina1 protein | LY | 1.3 |
| 17o01 | CA964986 | set and mynd domain containing 1 | SKF | 1.7 |
| 08i06 | CA968718 | set binding factor 2 | SKF | 1.8 |
| 08i06 | CA968718 | set binding factor 2 | LY | 1.3 |
| 21m03 | CA967953 | set translocation (myeloid leukemia-associated) | SKF | 1.6 |
| 22b22 | CA969258 | set translocation (myeloid leukemia-associated) | SKF | -1.6 |
| 08d11 | CA968645 | set translocation (myeloid leukemia-associated) | SKF | 1.4 |
| 08d11 | CA968645 | set translocation (myeloid leukemia-associated) | LY | 1.3 |
| 07i22 | CA964058 | sex hormone binding globulin | LY | 1.4 |
| 20f05 |  | sh3 and cysteine rich domain 3 | LY | 1.5 |
| 21a01 | CA967895 | sh3-domain grb2-like 2 | SKF | 1.7 |
| 21a01 | CA967895 | sh3-domain grb2-like 2 | LY | 1.3 |
| 19b03 | CA969080 | sh3-domain grb2-like endophilin b2 | SKF | 1.5 |
| 19b03 | CA969080 | sh3-domain grb2-like endophilin b2 | LY | 1.3 |
| 19c13 | CA966179 | signal peptide peptidase-like 2a | SKF | 1.5 |
| 11l19 | CF662641 | signal recognition particle receptor (docking protein) | SKF | 1.6 |
| 15f10 | CA965601 | signal sequencedelta | SKF | -1.8 |
| 20a21 | CA966252 | signal sequencedelta | LY | 1.3 |
| 21b04 | CA967238 | sin3a-associated18kda | SKF | 1.7 |
| 10e02 | CA967628 | slain motifmember 2 | SKF | 2 |
| 23d12 | CA969574 | slit and ntrk-likemember 6 | SKF | -1.9 |
| 15b01 | CA965548 | slow myosin heavy chain 1 | SKF | 1.7 |
| 06d05 | CF662471 | small glutamine-rich tetratricopeptide repeat-alpha | LY | 1.3 |
| 22o19 | CA969520 | small nuclear ribonucleoprotein polypeptide n | SKF | -1.5 |
| 22h08 | CA969371 | small subunit 1 | SKF | -1.9 |
| 10d19 | CA968857 | smc6 protein | SKF | -1.8 |
| 21h23 | CA969193 | smooth muscle alpha actin | LY | 1.3 |
| 18m09 | CA964709 | smt3 suppressor of mif two 3 homolog 3 | SKF | -1.8 |
| 11a15 | CA965919 | sodefrin-like protein precursor | SKF | 1.8 |
| 03h02 | DY231856 | solute carrier family 1 (glial high affinity glutamate transporter)member 3 | SKF | 1.8 |
| 08o03 | CA970416 | solute carrier family 13 (sodium-dependent citrate transporter)member 5 | SKF | 1.6 |
| 17n04 | CF662804 | solute carrier family 16 (monocarboxylic acid transporters)member 9 | SKF | -1.8 |
| 06k18 | CA965438 | solute carrier family 25 (mitochondrial carrier adenine nucleotide translocator)member 6 | LY | 1.5 |
| 17e15 | CA964942 | solute carrier family 25 (mitochondrial carrier phosphate carrier)member 3 | SKF | -1.5 |
| 15o04 | CA966771 | solute carrier family 25member 14 | LY | 1.5 |
| 05i23 | CA967248 | solute carrier family 25member 5 | LY | 1.5 |
| 10k08 | CA967682 | solute carrier family 27 (fatty acid transporter)member 1 | LY | 1.4 |
| 11f06 | CA966088 | solute carrier family 31 (copper transporters)member 2 | SKF | 1.5 |
| 24p05 | CA970010 | solute carrier family 6 (neurotransmitter transporter)member 19 | SKF | -1.7 |
| 24p05 | CA970010 | solute carrier family 6 (neurotransmitter transporter)member 19 | LY | 1.4 |
| 16c06 | CA966149 | solute carrier family 7 (cationic amino acidy+ system)member 10 | SKF | -1.5 |
| 17n07 | CA965784 | solute carrier familymember 19 | SKF | -1.5 |
| 19l01 | CA969129 | solute carrier familymember 2 | SKF | 2.1 |
| 08j22 | CA968742 | solute carrier familymember 3 | LY | 1.3 |
| 21i09 | CA967936 | solute carrier familymember 36 | LY | 1.5 |
| 09n01 | CF662593 | solute carrier familymember e2 | SKF | 2.3 |
| 18j12 | CF662822 | solute carrier familysodium bicarbonatemember 8 | SKF | -2.1 |
| 03j03 | DY231936 | somatostatin receptor 1 | SKF | 2.2 |
| 03p01 | DY231966 | somatostatin receptor 2 | SKF | 1.5 |
| 03i03 | DY231933 | somatostatin receptor 3 | SKF | 1.4 |
| 03k02 | FG392600 | somatostatin receptor 5 | SKF | 1.5 |
| 20b10 | CA966558 | sorcin | LY | 1.6 |
| 07k12 | CA964069 | sorting nexin 12 | SKF | -1.5 |
| 23k21 | CA968072 | sp1 transcription factor | LY | 1.7 |
| 04h06 | FG392760 | spectrin alpha 2 | SKF | 1.4 |
| 09k14 | CA964859 | spermidine spermine n1-acetyltransferase | SKF | 1.8 |
| 17b12 | CA967308 | sphingosine-1-phosphate receptor 4 | SKF | -1.8 |
| 17b01 | CA965738 | splicingarginine serine-rich 3 | SKF | 1.6 |
| 19d04 | CF662836 | splicingarginine serine-rich 5 | SKF | 1.6 |
| 15h02 | CA965621 | sry (sex determining region y)-box 2 | SKF | 1.5 |
| 14c03 | CA964744 | st3 beta-galactoside alpha--sialyltransferase 2 | SKF | 1.6 |
| 21h15 | CA969190 | stan | LY | 1.3 |
| 08m05 | CA970407 | staphylococcal nuclease domain containing 1 | SKF | 1.5 |
| 24i19 | CA969885 | StAR-related lipid transferdomain containing 4 | LY | 1.6 |
| 24i19 | CA969885 | StAR-related lipid transferdomain containing 4 | SKF | -2.1 |
| 19a07 | CA966170 | stathmin 1 oncoprotein 18 variant 8 | SKF | 1.6 |
| 24e15 | CA969799 | stathmin 1 oncoprotein 18 variant 8 | LY | 1.4 |
| 15h03 | CA965622 | stathmin 1 oncoprotein 18 variant 8 | SKF | 1.4 |
| 20o20 | CA966805 | stathmin 1 oncoprotein 18 variant 8 | SKF | -1.5 |
| 05e09 | CA965510 | stearoyl-desaturaseame | SKF | -2 |
| 16f18 | CF662762 | step ii splicing factor slu7 | SKF | 1.7 |
| 16f18 | CF662762 | step ii splicing factor slu7 | LY | 1.3 |
| 12a17 | CA967736 | steroid 5-alpha-reductase 2 | SKF | 1.5 |
| 09i03 | CA964141 | sterol carrier protein 2 | SKF | 1.9 |
| 05g10 | CA967525 | sterol carrier protein 2 | SKF | -1.6 |
| 09n02 | CA964885 | sterol-c5-desaturase (fungaldelta-5-desaturase) homolog | SKF | 2.5 |
| 15l02 | CA965668 | structural maintenance of chromosomes 3 | LY | 1.5 |
| 15l02 | CA965668 | structural maintenance of chromosomes 3 | SKF | 1.5 |
| 19a02 | CA964992 | sub1 homolog | SKF | 1.9 |
| 10e10 | CA967634 | subfamilymember 12 | SKF | -1.6 |
| 16k01 | CA965490 | subfamilymember 3b | SKF | 2.3 |
| 18i12 | CA964670 | subfamilymember 5 | SKF | -1.7 |
| 24m05 | CA969954 | succinate dehydrogenasesubunitintegral membrane protein | LY | 1.5 |
| 24n12 | CA969980 | succinate dehydrogenasesubunitintegral membrane protein | SKF | -1.7 |
| 24i17 | CA969883 | sucrase-isomaltase (alpha-glucosidase) | SKF | -2 |
| 10d10 | CA964195 | sulfotransferase familymember 1 | SKF | -2.1 |
| 18e11 | CA964628 | superoxide dismutasesoluble | LY | 1.5 |
| 19g02 | CA965023 | suppression of tumorigenicity 13 (colon carcinoma) (hsp70 interacting protein) | SKF | 2.6 |
| 19h01 | CA969109 | suppression of tumorigenicity 13 (colon carcinoma) (hsp70 interacting protein) | LY | 1.3 |
| 12b04 | CF662664 | suppressor of cytokine signaling 1 | SKF | 1.6 |
| 06h08 | CF662490 | suppressor ofdomain containing 1 | LY | 1.4 |
| 12p21 | CA966062 | surfeit 4 | SKF | 1.6 |
| 20l10 | CA966521 | survival motor neuron domain containing 1 | LY | 1.5 |
| 20f03 | CA967126 | sympk protein | SKF | 2.6 |
| 20f03 | CA967126 | sympk protein | LY | 1.3 |
| 18g15 | CA964653 | synaptic ras gtpase activating protein 1 homologisoform cra_c | LY | 1.4 |
| 14g04 | CA964385 | synaptogyrin 2 | SKF | 1.5 |
| 15b14 | CA965553 | synaptogyrin 3 | SKF | 2.6 |
| 03j17 | DY231978 | synaptosomal-associated protein 25 | SKF | 1.5 |
| 19n13 | CA969142 | synaptosomal-associated protein 25 | LY | 1.4 |
| 06o17 | CF662536 | syndecan binding protein | LY | 1.7 |
| 17i07 | CA964959 | syne2 protein | SKF | -1.7 |
| 21d01 | CA967076 | syntaxin 4 | SKF | -1.5 |
| 24h15 | CA969860 | synuclein, beta | SKF | 1.5 |
| 12c20 | CA970236 | tbc1 domainmember 23 | LY | 1.3 |
| 08d02 | CA968637 | tbt-binding protein | LY | 1.3 |
| 23l20 | CA969665 | tcb1 transposase | LY | 1.4 |
| 24m22 | CA967079 | t-cellhomeobox 3b | SKF | -1.5 |
| 23p12 | CA969697 | temporarily assigned gene name family member (tag-241) | SKF | -2.4 |
| 07i14 | CA964052 | testis enhanced gene transcript (bax inhibitor 1) | SKF | 2.2 |
| 06j21 | CF662507 | testis enhanced gene transcript (bax inhibitor 1) | SKF | -2.2 |
| 06j21 | CF662507 | testis enhanced gene transcript (bax inhibitor 1) | LY | 1.6 |
| 15n19 | CA965708 | tetraspanin 1 | LY | 1.3 |
| 15p01 | CA965719 | tetraspanin 18 | SKF | 2.5 |
| 16n11 | CA965499 | tetraspanin 3 | LY | 1.5 |
| 18n24 | CA969057 | tetraspanin 33 | SKF | -1.6 |
| 20o02 | CA965906 | tetraspanin 9 | SKF | 1.6 |
| 23j22 | CA969645 | tetratricopeptide repeat domain 17 | LY | 1.5 |
| 13l13 | CA966391 | tgf beta-inducible nuclear protein 1 | SKF | 1.7 |
| 09d18 | CA964792 | thioredoxin | LY | 1.4 |
| 22l10 | CA967067 | thioredoxin 2 | LY | 1.5 |
| 08j23 | CA968743 | thioredoxin domain containing 17 | LY | 1.5 |
| 22p05 | CA969528 | thioredoxin domain containing 9 | LY | 1.3 |
| 07k18 | CA964074 | thioredoxin interacting protein | LY | 1.7 |
| 07k18 | CA964074 | thioredoxin interacting protein | SKF | -2 |
| 23k11 | CA968063 | thioredoxin-like 1 | SKF | -1.5 |
| 07h03 | CA968505 | threonyl-trna synthetase | SKF | 2.2 |
| 09p19 | CF662599 | tight junction protein 2 | SKF | -1.7 |
| 23i21 | CA968052 | titin-cap | SKF | -1.7 |
| 21j07 | CA966806 | titin-cap | LY | 1.3 |
| 22j09 | CA969410 | tmem214 protein | LY | 1.4 |
| 23b22 | CA969561 | tnf receptor-associated factor 1 | LY | 1.5 |
| 11p13 | CF662659 | tnf receptor-associated factor 4 | SKF | 2.1 |
| 20a13 | CA966248 | tnnt3b protein | SKF | 2.6 |
| 18l16 | CA969033 | tnnt3b protein | LY | 1.6 |
| 08m07 | CA970408 | tocopheroltransfer protein | SKF | 1.6 |
| 21f09 | CF662887 | tp53 dependent g2 arrest mediator candidate | LY | 1.7 |
| 22l21 | CA969459 | tp53 regulating kinase | SKF | -1.9 |
| 21j09 | CA969196 | tpm1 protein | LY | 1.4 |
| 24i22 | CA969888 | transaldolase 1 | SKF | -1.6 |
| 05c17 | CA967498 | transducer of1 | LY | 1.3 |
| 07o03 | CA964096 | transferrin variant c | SKF | 2 |
| 08a13 | CA968595 | transferrin variant c | SKF | 1.9 |
| 06l19 | CA967017 | transferrin variant c | LY | 1.5 |
| 09k21 | CA964159 | transferrin variant c | LY | 1.4 |
| 07b16 | CA968458 | transketolase-like 2 | LY | 1.5 |
| 12f02 | CA966346 | translocase of inner mitochondrial membrane 17 homolog a | SKF | 1.6 |
| 07o17 | CA965516 | translocase of inner mitochondrial membrane 23 homolog | SKF | 1.6 |
| 21a17 | CA967902 | translocase of outer mitochondrial membrane 22 homolog | LY | 1.3 |
| 19c02 | CA965003 | translocase of outer mitochondrial membrane 34 | SKF | 3.1 |
| 09j06 | CA964845 | translocase of outer mitochondrial membrane 34 | LY | 1.3 |
| 09c06 | CA964778 | translocation associated membrane protein 1 | SKF | 1.4 |
| 09c06 | CA964778 | translocation associated membrane protein 1 | LY | 1.3 |
| 14l04 | CA964482 | transmembrane 4 superfamily member 4 | SKF | 1.6 |
| 18l05 | CA969024 | transmembrane 4 superfamily member 4 | SKF | 1.6 |
| 20k02 | CA965894 | transmembrane 4 superfamily member 4 | LY | 1.3 |
| 10l05 | CA968894 | transmembrane 6 superfamily member 2 | SKF | -1.5 |
| 22h16 | CA969377 | transmembrane and tetratricopeptide repeat containing 4 | SKF | -1.6 |
| 12h17 | CA966023 | transmembrane emp24 protein transport domain containing 3 | SKF | 1.6 |
| 09a03 | CA964105 | transmembrane protein 106b | LY | 1.5 |
| 08f07 | CA968669 | transmembrane protein 115 | LY | 1.4 |
| 08f07 | CA968669 | transmembrane protein 115 | SKF | 1.4 |
| 19a03 | CA966168 | transmembrane protein 179 | SKF | 2.2 |
| 23o04 | CA968098 | transmembrane protein 179 | LY | 1.5 |
| 05e13 | CA967512 | transmembrane protein 188 | SKF | 1.7 |
| 08j15 | CA968737 | transmembrane protein 209 | SKF | 2 |
| 08j15 | CA968737 | transmembrane protein 209 | LY | 1.6 |
| 19b02 | CA965795 | transmembrane protein 27 | SKF | 2.1 |
| 19b02 | CA965795 | transmembrane protein 27 | LY | 1.3 |
| 20c16 | CA965872 | transmembrane protein 47 | SKF | 1.6 |
| 13k04 | CA966437 | transmembrane protein 49 | SKF | 1.4 |
| 07i18 | CA964055 | transmembrane protein 50a | LY | 1.3 |
| 09m24 | CA964884 | transmembrane protein 56 | LY | 1.3 |
| 11i05 | CA965955 | transmembrane protein 85 | SKF | 1.8 |
| 11n14 | CA966132 | transmembrane protein 90a | SKF | 1.9 |
| 23n15 | CA969680 | transmembrane protein 93 | LY | 1.4 |
| 22m16 | CA969474 | transmembrane protein with egf-like and two follistatin-like domains 1 | LY | 1.4 |
| 12f01 | CA966004 | transthyretin | SKF | 3 |
| 09m06 | CA964876 | transthyretin | SKF | 1.6 |
| 09m06 | CA964876 | transthyretin | LY | 1.4 |
| 13p22 | CF662720 | triosephosphate isomerase | SKF | -2 |
| 07h01 | CA968504 | triosephosphate isomerase 1b | SKF | 1.8 |
| 06b07 | CF662467 | tripartite motif-containing 33 | LY | 1.4 |
| 14p06 | CA964561 | tripartite motif-containing 54 | LY | 1.4 |
| 14p06 | CA964561 | tripartite motif-containing 54 | SKF | 1.5 |
| 23n20 | CA969683 | tripartite motif-containing 63 | LY | 1.3 |
| 06g17 | CF662487 | tripartite motif-containing protein 16 (estrogen-responsive b box protein) | LY | 1.4 |
| 12p08 | CA966349 | trna selenocysteine associated protein 1 | SKF | 1.5 |
| 12a22 | CA970226 | trna-yw synthesizing protein 1 homolog | LY | 1.5 |
| 13a10 | CF662682 | tropomodulin 4 | SKF | 1.5 |
| 14f24 | CA964383 | troponin c type 2 | SKF | -2.1 |
| 21g17 | CA967929 | troponin c type 2 | SKF | 1.6 |
| 14n04 | CA964522 | troponin i | SKF | 1.8 |
| 14e04 | CA964344 | troponinfast | SKF | 2 |
| 13p14 | CF662719 | troponinfast | LY | 1.4 |
| 13h19 | CF662705 | troponinfast | SKF | -1.5 |
| 12l23 | CA966045 | trypsinogen i | LY | 1.7 |
| 06n01 | CF662522 | tryptophan-dioxygenase | LY | 1.6 |
| 06m17 | CF662520 | tryptophan-dioxygenase | LY | 1.4 |
| 03e03 | DY231829 | tubulin alpha 6 | SKF | 1.5 |
| 03o23 | FG392672 | tubulin beta 1 | SKF | -1.5 |
| 20e09 | CA966269 | tubulin beta 2c | LY | 1.5 |
| 11a13 | CA965918 | tubulin-specific chaperone a | SKF | 1.4 |
| 16g14 | CA967225 | tubulin-specific chaperone e | SKF | 2 |
| 09o01 | CA964172 | tumor differentially expressed 2-like | LY | 1.5 |
| 19b14 | CA965800 | tumor protein p53 binding protein 1 | SKF | 2.3 |
| 05o02 | CA967581 | tumortranslationally-controlled 1 | SKF | 1.5 |
| 22o12 | CA969513 | tweety homolog 3 | SKF | -1.7 |
| 14f02 | CA964363 | u2small nuclear rna auxiliary factor 1 | SKF | 2.4 |
| 14f02 | CA964363 | u2small nuclear rna auxiliary factor 1 | LY | 1.3 |
| 14a16 | CA964279 | ubiquinol-cytochrome c reductase core protein i | SKF | 1.6 |
| 10l04 | CA964234 | ubiquinol-cytochrome crieske iron-sulfur polypeptide 1 | SKF | -1.9 |
| 22k12 | CA969428 | ubiquitin fusion degradation 1-like | SKF | -1.6 |
| 16n18 | CA966457 | ubiquitin specific peptidase 12 | SKF | 1.8 |
| 17n15 | CF662805 | ubiquitin specific protease 1 | SKF | -1.4 |
| 24n09 | CA969977 | ubiquitin-activating enzyme e1 | LY | 1.4 |
| 12h15 | CA966022 | ubiquitin-conjugating enzyme e2 variant 1 | SKF | 2.2 |
| 12h15 | CA966022 | ubiquitin-conjugating enzyme e2 variant 1 | LY | 1.3 |
| 22j08 | CA969409 | ubiquitin-conjugating enzyme e2b | SKF | -1.7 |
| 09a14 | CA964759 | ubiquitin-conjugating enzyme e2d 1 (ubc4 5yeast) | SKF | 1.9 |
| 24b06 | CA969734 | ubiquitin-conjugating enzyme e2d 1 (ubc4 5yeast) | LY | 1.4 |
| 12h03 | CA966017 | ubiquitin-conjugating enzyme e2g 1 | SKF | 1.9 |
| 08b10 | CA968610 | ubiquitin-conjugating enzyme e2g 1 | LY | 1.6 |
| 16b02 | CF662745 | ubiquitin-conjugating enzyme e2g 2 | SKF | 2 |
| 08c14 | CA968630 | ubiquitin-conjugating enzyme e2h | SKF | 1.4 |
| 03c04 | DY231811 | ubiquitin-conjugating enzyme e2n | SKF | 2.1 |
| 08o20 | CA968819 | udp glucuronosyltransferase 2polypeptide a3 | SKF | 1.8 |
| 08o20 | CA968819 | udp glucuronosyltransferase 2polypeptide a3 | LY | 1.3 |
| 05c10 | CA967491 | udp-glucose pyrophosphorylase 2 | LY | 1.4 |
| 16e15 | CA966398 | unc-45 homolog b | SKF | 1.6 |
| 19n14 | CA965850 | unc-84 homolog b | SKF | 2.5 |
| 16a01 | CF662742 | uncharacterized proteinpartial | SKF | 2.4 |
| 06n03 | CF662523 | uncoupling protein 2 | LY | 1.7 |
| 12f06 | CF662667 | urocanase domain containing 1 | SKF | 2.4 |
| 09o11 | CA964176 | urocanase domain containing 1 | LY | 1.4 |
| 22l09 | CA969449 | vacuolar protein sorting 13c | SKF | -1.5 |
| 19n15 | CA967270 | vacuolar protein sorting 26 homolog a | SKF | 1.6 |
| 23e20 | CA968011 | vacuolar protein sorting 28 | LY | 1.5 |
| 20j02 | CA966560 | vacuolar protein sorting 4a | LY | 1.6 |
| 20j02 | CA966560 | vacuolar protein sorting 4a | SKF | 1.7 |
| 24o22 | CA970006 | valosin containing protein | LY | 1.3 |
| 11o11 | CA965980 | vamp (vesicle-associated membrane protein)-associated protein33kda | SKF | -1.6 |
| 24c22 | CA969769 | v-erb-a erythroblastic leukemia viral oncogene homolog 4 | LY | 1.6 |
| 21o19 | CA967969 | villin 1 | SKF | -1.7 |
| 24i24 | CA969890 | vimentin | SKF | -1.7 |
| 14j12 | CA964445 | vimentin | SKF | -1.5 |
| 05a04 | CA967474 | vitamin d-binding protein | SKF | 1.4 |
| 08d13 | CA968647 | vitamin k epoxide reductasesubunit 1-like 1 | SKF | 2.4 |
| 09e16 | CA964803 | vitamin k epoxide reductasesubunit 1-like 1 | SKF | 1.6 |
| 06a05 | CA965405 | vitelline membrane outer layer 1 homolog | LY | 1.6 |
| 12o07 | CA967800 | vitelline membrane outer layer 1 homolog | SKF | 1.4 |
| 08j01 | CA968726 | vitellogenin 1 | LY | 1.3 |
| 08j01 | CA968726 | vitellogenin 1 | SKF | 1.5 |
| 12i13 | CA967775 | vitellogenin 2 | SKF | 1.4 |
| 06d17 | CF662474 | vitronectin | LY | 1.3 |
| 11a01 | CA965541 | voltage-dependent anion channel 2 | SKF | 1.7 |
| 06h05 | CA965192 | warm-temperature-acclimation-related-65 kda-protein | SKF | -1.7 |
| 06e15 | CA965420 | warm-temperature-acclimation-related-65 kda-protein | LY | 1.3 |
| 15d13 | CA965580 | wd repeat domain 44 | SKF | 3 |
| 04n16 | FG392999 | wd repeat domain 68 | SKF | 1.5 |
| 12n20 | CA966428 | wilms tumor 1 associated protein | SKF | 1.4 |
| 20p11 | CA966862 | wnt1 inducible signaling pathway protein 1 | SKF | 1.5 |
| 08p05 | CA968824 | ww domain binding protein 2 | SKF | -1.8 |
| 08p05 | CA968824 | ww domain binding protein 2 | LY | 1.5 |
| 20a19 | CA966251 | xerodermacomplementation group a | SKF | 1.5 |
| 08d15 | CA968648 | yip1 domainmember 4 | SKF | 2.3 |
| 08d15 | CA968648 | yip1 domainmember 4 | LY | 1.3 |
| 10n12 | CA964250 | ylp motif containing 1 | SKF | -2 |
| 08c07 | CA968624 | zeta (quinone reductase)-like 1 | SKF | 1.6 |
| 08c07 | CA968624 | zeta (quinone reductase)-like 1 | LY | 1.3 |
| 12e13 | CA967753 | zgc:101710 protein | SKF | 2.2 |
| 11j03 | CA966339 | zgc:110410 protein | SKF | 1.7 |
| 11j03 | CA966339 | zgc:110410 protein | LY | 1.3 |
| 06o09 | CF662534 | zgc:112265 protein | LY | 1.3 |
| 04p15 | FG393019 | zgc:123194 | SKF | 1.6 |
| 08d09 | CA968643 | zgc:152778 protein | SKF | 1.6 |
| 17j23 | CA965774 | zgc:153426 | SKF | -1.5 |
| 11g15 | CA965949 | zgc:154001 | SKF | 1.8 |
| 22l16 | CA969454 | zgc:162162 protein | LY | 1.3 |
| 08b08 | CA968608 | zgc:162255 protein | LY | 1.3 |
| 12l15 | CA966041 | zgc:162613 protein | SKF | 1.4 |
| 12e15 | CA967754 | zgc:162730 protein | SKF | 1.7 |
| 21o21 | CA967970 | zgc:163001 protein | LY | 1.4 |
| 05n01 | CA965396 | zgc:163027 protein | SKF | 1.6 |
| 12p17 | CA966060 | zgc:171563 protein | SKF | 2.4 |
| 12d03 | CA965994 | zgc:171680 protein | SKF | 2.1 |
| 20b22 | CA966519 | zgc:172056 protein | SKF | 1.8 |
| 20b22 | CA966519 | zgc:172056 protein | LY | 1.3 |
| 16g03 | CA966372 | zgc:172067 protein | SKF | 1.6 |
| 21l01 | CA966807 | zgc:55557 protein | SKF | 3.3 |
| 08p15 | CA968833 | zgc:92624 protein | SKF | 2.2 |
| 08p15 | CA968833 | zgc:92624 protein | LY | 1.3 |
| 19o08 | CA965067 | zinc and double phd fingers family 2 | SKF | 1.5 |
| 23a24 | CA967982 | zinc finger ccch-type containing 7a | SKF | -2.3 |
| 23j13 | CA969637 | zinc finger protein 135 (zinc finger protein 61) (zinc finger protein 78-like 1) | LY | 1.4 |
| 23l22 | CA969667 | zinc finger protein 366 | LY | 1.4 |
| 15i14 | CA965639 | zinc finger protein 782 | SKF | 2 |
| 11l13 | CF662639 | zinc metallopeptidase | SKF | 2.2 |
| 08o10 | CA968814 | zinccchc domain containing 12 | SKF | 2 |
| 08o10 | CA968814 | zinccchc domain containing 12 | LY | 1.3 |
| 19n11 | CA966799 | zinccchc domain containing 13 | SKF | -1.5 |
| 09e06 | CA964798 | zinccchc domain containing 9 | LY | 1.3 |
| 20c13 | CA966260 | zona pellucida glycoprotein | SKF | 1.7 |
| 10p12 | CA964260 | zyg-11 homolog b | SKF | -1.6 |
| 13j05 | CA966436 | zymogen granule membrane glycoprotein 2 | SKF | 1.8 |
