# Supplementary material for: Rapid Dopaminergic Modulation of the Fish Hypothalamic Transcriptome and Proteome
Source: PLoS One. 2010 Aug 20;5(8):e12338. doi: 10.1371/journal.pone.0012338 (PMC2924890; doi:10.1371/journal.pone.0012338)
Supplement: Table S2 — All goldfish proteins identified in the hypothalamus in this study. Proteins in which a single peptide was used in identification are also presented in this table. % Cov is the amount of amino acid coverage (%) by peptides. Ratios (e.g. 115∶114) are each treatment (tag 115 or 117) divided by control (tag 114) to obtain relative fold change. Pval is the p-value after all peptides for a protein were used for quantitation. The Error Factor (EF) expresses the 95% uncertainty range for a reported ratio. The true protein ratio is expected to be found between the (reported ratio)*(EF) and the (reported ratio)/(EF) 95% of the time. Peptides used in quantification also included all peptides with post-translational modifications and all charge states (Dataset S1). Peptides that do not have a Ratio or P-value were not quantified because 1) peptide signal was too low; 2) peptide did not meet standard for quantitation; or 3) peptide belonged to more than one unique protein. (0.75 MB DOC) [file pone.0012338.s006.doc]

| **NCBI Accession** | **Name of Protein** | **% Cov** | **115:114** | **PVal 115:114** | **EF 115:114** | **117:114** | **PVal 117:114** | **EF 117:114** |
| --- | --- | --- | --- | --- | --- | --- | --- | --- |
| gi|58802481 | 14 kDa apolipoprotein [Carassius auratus gibelio] | 48.94 | 0.667 | 0.001 | 1.175 | 0.796 | 0.003 | 1.123 |
| gi|45602838 | 14-3-3 protein [Carassius auratus] | 57.79 | 1.209 |  |  | 1.452 |  |  |
| gi|46326988 | 14-3-3 protein [Oncorhynchus mykiss] | 69.02 |  |  |  |  |  |  |
| gi|34452065 | 14-3-3C1 protein [Oncorhynchus mykiss] | 51.84 |  |  |  |  |  |  |
| gi|7768841 | 20S proteasome beta 6 subunit [Carassius auratus] | 25.74 |  |  |  |  |  |  |
| gi|55925442 | 3-oxoacid CoA transferase 1 [Danio rerio] | 14.29 |  |  |  |  |  |  |
| gi|60417184 | 40S ribosomal protein S14 [Platichthys flesus] | 27.81 |  |  |  |  |  |  |
| gi|41018101 | 40S ribosomal protein S18 | 30.26 | 1.395 |  |  | 1.507 |  |  |
| gi|82188463 | 40S ribosomal protein S21 | 22.22 |  |  |  |  |  |  |
| gi|51316652 | 40S ribosomal protein S25 | 13.25 | 0.952 |  |  | 0.852 | 0.740 | 111.008 |
| gi|54039530 | 40S ribosomal protein S8 | 11.54 | 1.137 | 0.751 | 51.433 | 1.485 | 0.504 | 161.665 |
| gi|48476439 | 40S ribosomal protein Sa-like protein [Sparus aurata] | 14.97 | 1.140 | 0.088 | 1.261 | 0.745 | 0.223 | 3.920 |
| gi|21263935 | 60S ribosomal protein L18 | 10.11 |  |  |  |  |  |  |
| gi|67764180 | 60S ribosomal protein L30 [Siniperca chuatsi] | 24.14 |  |  |  |  |  |  |
| gi|51230650 | acetyl-Coenzyme A acetyltransferase 1 (acetoacetyl Coenzyme A thiolase) [Danio rerio] | 19.52 | 1.029 | 0.904 | 10.971 | 0.898 | 0.733 | 21.787 |
| gi|5880681 | acidic ribosomal phophoprotein P0 [Danio rerio] | 3.13 | 1.799 |  |  | 2.133 |  |  |
| gi|77993336 | aconitase 1, soluble [Danio rerio] | 5.06 |  |  |  |  |  |  |
| gi|38707983 | aconitase 2, mitochondrial [Danio rerio] | 21.99 | 1.125 | 0.461 | 1.494 | 1.021 | 0.925 | 1.924 |
| gi|47086645 | actin related protein 2/3 complex, subunit 2 [Danio rerio] | 12.37 | 0.762 |  |  | 0.999 |  |  |
| gi|92087016 | Actin, cytoplasmic 2 (Beta-actin-2) | 58.13 | 1.122 | 0.199 | 1.199 | 1.093 | 0.159 | 1.136 |
| gi|41054603 | actinin alpha 4 [Danio rerio] | 13.65 |  |  |  |  |  |  |
| gi|47086877 | adaptor protein complex AP-2, mu1 [Danio rerio] | 15.83 | 1.618 |  |  | 2.289 |  |  |
| gi|46559756 | adaptor-related protein complex 2, beta 1 subunit [Danio rerio] | 9.99 | 1.321 | 0.176 | 1.601 | 1.268 | 0.005 | 1.103 |
| gi|59276030 | ADP-ribosylation factor 3 [Danio rerio] | 43.65 | 0.815 | 0.240 | 1.468 | 0.895 | 0.337 | 1.297 |
| gi|94734006 | alanyl-tRNA synthetase [Danio rerio] | 7.35 |  |  |  |  |  |  |
| gi|41282154 | aldolase a, fructose-bisphosphate [Danio rerio] | 44.78 | 0.742 | 0.491 | 40.065 | 0.646 | 0.504 | 276.713 |
| gi|6683668 | alpha 4 subunit of 20S proteasome [Carassius auratus] | 26.29 | 0.911 | 0.815 | 4.480 | 1.173 |  |  |
| gi|27733675 | alpha actin [Notothenia coriiceps] | 45.62 |  |  |  |  |  |  |
| gi|449151 | alpha globin | 48.25 | 1.377 | 0.082 | 1.522 | 1.004 | 0.991 | 27.229 |
| gi|10242174 | alpha tubulin [Notothenia coriiceps] | 58.02 |  |  |  |  |  |  |
| gi|55962545 | alpha-tropomyosin [Danio rerio] | 41.94 |  |  |  |  |  |  |
| gi|62955353 | aminopeptidase-like 1 [Danio rerio] | 4.57 | 1.502 |  |  | 1.306 |  |  |
| gi|62199406 | annexin [Oncorhynchus tshawytscha] | 9.44 | 0.820 |  |  | 0.877 |  |  |
| gi|46329651 | Annexin A11b [Danio rerio] | 4.12 |  |  |  |  |  |  |
| gi|32451741 | Annexin A4 [Danio rerio] | 8.41 | 0.936 |  |  | 1.364 |  |  |
| gi|129560469 | anserinase [Anguilla japonica] | 6.49 |  |  |  |  |  |  |
| gi|53733942 | Apoa protein [Danio rerio] | 11.98 |  |  |  | 1.023 |  |  |
| gi|6688890 | apolipoprotein E [Oncorhynchus mykiss] | 17.09 | 0.996 | 0.971 | 3.145 | 0.892 | 0.588 | 6.819 |
| gi|47087281 | ARP2 actin-related protein 2 homolog [Danio rerio] | 4.06 | 1.699 |  |  |  |  |  |
| gi|54400426 | ATP synthase, H+ transporting, mitochondrial F0 complex, subunit b, isoform 1 [Danio rerio] | 19.84 |  |  |  |  |  |  |
| gi|41152334 | ATP synthase, H+ transporting, mitochondrial F0 complex, subunit d [Danio rerio] | 41.61 | 0.723 | 0.223 | 2.220 | 0.685 | 0.134 | 1.948 |
| gi|49904605 | ATP synthase, H+ transporting, mitochondrial F0 complex, subunit g [Danio rerio] | 29.13 | 0.731 | 0.413 | 20.560 | 0.738 | 0.144 | 2.441 |
| gi|116325975 | ATP synthase, H+ transporting, mitochondrial F1 complex, alpha subunit 1, cardiac muscle [Danio rerio] | 37.75 |  |  |  |  |  |  |
| gi|33504537 | ATPase, H+ transporting, lysosomal 56/58kDa, V1 subunit B2 [Danio rerio] | 41.45 | 1.097 | 0.592 | 1.881 | 1.117 | 0.498 | 1.580 |
| gi|47086697 | ATPase, H+ transporting, lysosomal V0 subunit a isoform 1 [Danio rerio] | 14.63 |  |  |  |  |  |  |
| gi|47940367 | ATPase, H+ transporting, lysosomal, V1 subunit B, member a [Danio rerio] | 37.94 |  |  |  |  |  |  |
| gi|55962503 | ATPase, H+ transporting, lysosomal, V1 subunit E isoform 1 [Danio rerio] | 47.35 | 1.039 | 0.785 | 1.411 | 1.225 | 0.159 | 1.362 |
| gi|47937977 | ATPase, H+ transporting, lysosomal, V1 subunit H [Danio rerio] | 24.62 | 1.056 | 0.736 | 4.793 | 1.264 | 0.557 | 35.108 |
| gi|55249973 | ATPase, Na+/K+ transporting, alpha 1b polypeptide [Danio rerio] | 46.34 | 1.200 |  |  | 1.076 |  |  |
| gi|94733516 | ATPase, Na+/K+ transporting, alpha 3a polypeptide [Danio rerio] | 48.00 | 1.186 | 0.310 | 1.724 | 1.160 | 0.258 | 1.504 |
| gi|40352936 | ATPase, Na+/K+ transporting, beta 2b polypeptide [Danio rerio] | 7.88 | 1.025 | 0.858 | 3.954 | 0.993 | 0.984 | 43.986 |
| gi|71564498 | beta thymosin-like protein [Danio rerio] | 61.36 | 0.751 | 0.018 | 1.237 | 0.717 | 0.231 | 1.923 |
| gi|5923889 | beta-2 tubulin [Gadus morhua] | 57.66 | 1.040 |  |  | 1.611 |  |  |
| gi|81097752 | Bhmt protein [Danio rerio] | 5.50 |  |  |  |  |  |  |
| gi|84570141 | brain-specific fatty acid binding protein [Oryzias latipes] | 49.24 | 0.745 | 0.248 | 4.630 | 0.820 | 0.455 | 8.923 |
| gi|8809798 | brain-type fatty-acid binding protein; B-Fabp [Danio rerio] | 78.03 | 0.699 | 0.001 | 1.156 | 0.820 | 0.155 | 1.347 |
| gi|50540420 | branched chain aminotransferase 2, mitochondrial [Danio rerio] | 8.19 | 1.405 |  |  | 0.948 |  |  |
| gi|94733104 | calbindin 2, like [Danio rerio] | 33.95 | 0.910 | 0.140 | 1.140 | 0.828 | 0.015 | 1.152 |
| gi|71834408 | calcium/calmodulin-dependent protein kinase (CaM kinase) II gamma a [Danio rerio] | 27.86 |  |  |  |  |  |  |
| gi|82524371 | calcium/calmodulin-dependent protein kinase II alpha [Danio rerio] | 41.21 | 1.773 | 0.003 | 1.305 | 2.336 | 0.004 | 1.474 |
| gi|78099194 | Calmodulin (CaM) | 65.10 | 0.818 | 0.004 | 1.142 | 0.862 | 0.059 | 1.167 |
| gi|41054003 | Cap1 CAP, adenylate cyclase-associated protein 1 [Danio rerio] | 9.50 | 1.419 |  |  | 0.929 |  |  |
| gi|41053959 | capping protein (actin filament) muscle Z-line, beta [Danio rerio] | 23.44 | 1.396 | 0.549 | 140.697 | 1.968 |  |  |
| gi|213020 | Carassius auratus gene, complete cds.], gene product | 14.79 | 1.004 | 0.983 | 5.410 | 0.971 | 0.283 | 1.193 |
| gi|73762632 | carbonic anhydrase [Cyprinus carpio] | 15.00 | 0.673 | 0.550 | 368.995 | 0.619 |  |  |
| gi|94983869 | catalase [Anguilla anguilla] | 14.78 |  |  |  |  |  |  |
| gi|9622234 | catalase [Danio rerio] | 12.55 |  |  |  |  |  |  |
| gi|66392176 | cell division cycle 42 [Danio rerio] | 31.41 |  |  |  |  |  |  |
| gi|41055439 | cell division cycle 42 [Danio rerio] | 30.89 |  |  |  |  |  |  |
| gi|40217926 | cellular retinoic acid-binding protein; CRABP [Astatotilapia burtoni] | 22.63 |  |  |  |  |  |  |
| gi|42627881 | chaperonin containing TCP1, subunit 2 (beta) [Danio rerio] | 11.40 | 1.340 |  |  |  |  |  |
| gi|31419227 | Chaperonin containing TCP1, subunit 3 (gamma) [Danio rerio] | 17.86 | 1.425 |  |  | 1.290 |  |  |
| gi|47939393 | Chaperonin containing TCP1, subunit 6A (zeta 1) [Danio rerio] | 18.27 | 1.161 |  |  | 1.064 |  |  |
| gi|41055032 | chaperonin containing TCP1, subunit 8 (theta) [Danio rerio] | 15.57 |  |  |  |  |  |  |
| gi|41054571 | citrate synthase [Danio rerio] | 17.74 | 0.967 | 0.772 | 1.347 | 0.891 | 0.519 | 1.655 |
| gi|53292609 | clathrin, heavy polypeptide (Hc) [Danio rerio] | 14.58 |  |  |  |  |  |  |
| gi|85719983 | coactosin-like 1 [Ictalurus punctatus] | 17.78 | 0.926 | 0.535 | 2.968 | 0.840 | 0.139 | 1.632 |
| gi|47271384 | cofilin 2, like [Danio rerio] | 44.85 | 1.109 | 0.349 | 1.345 | 0.996 | 0.955 | 1.212 |
| gi|14164347 | collagen a1(I) [Oncorhynchus mykiss] | 10.35 |  |  |  |  |  |  |
| gi|56790315 | collagen, type I, alpha 1 [Danio rerio] | 14.93 |  |  |  |  |  |  |
| gi|66736126 | collapsin response mediator protein 2 [Danio rerio] | 38.57 | 1.117 | 0.146 | 1.175 | 1.053 | 0.723 | 1.530 |
| gi|24210478 | connexin-43 [Carassius auratus] | 9.24 | 1.987 |  |  | 2.067 |  |  |
| gi|94732345 | COP9 constitutive photomorphogenic homolog subunit 7A [Danio rerio] | 13.65 |  |  |  |  |  |  |
| gi|51870938 | cortactin [Danio rerio] | 9.33 | 0.518 |  |  | 0.878 |  |  |
| gi|31322097 | creatine kinase muscle isoform 1 [Chaenocephalus aceratus] | 32.11 |  |  |  |  |  |  |
| gi|31322099 | creatine kinase muscle isoform 2 [Chaenocephalus aceratus] | 34.65 | 1.050 |  |  | 0.670 |  |  |
| gi|55962715 | creatine kinase, brain [Danio rerio] | 61.68 | 0.917 | 0.737 | 12.287 | 0.785 | 0.078 | 1.461 |
| gi|38488694 | creatine kinase, mitochondrial 1 [Danio rerio] | 33.33 | 1.239 | 0.028 | 1.193 | 1.190 | 0.186 | 1.355 |
| gi|47087305 | cullin-associated and neddylation-dissociated 1 [Danio rerio] | 4.07 | 2.373 |  |  | 2.855 |  |  |
| gi|60392159 | Cytochrome c | 71.15 | 0.880 | 0.658 | 2.923 | 0.987 | 0.735 | 1.469 |
| gi|86450352 | cytochrome c oxidase subunit II [Opsariichthys bidens] | 6.09 | 1.214 |  |  | 1.082 |  |  |
| gi|94733032 | cytochrome c oxidase subunit IV isoform 1 [Danio rerio] | 28.40 | 0.999 | 0.995 | 1.801 | 1.210 | 0.042 | 1.174 |
| gi|41393167 | dihydrolipoamide dehydrogenase [Danio rerio] | 11.05 | 0.857 | 0.634 | 3.302 | 1.384 |  |  |
| gi|47086703 | dihydrolipoamide S-acetyltransferase (E2 component of pyruvate dehydrogenase complex) [Danio rerio] | 12.73 |  |  |  |  |  |  |
| gi|66472750 | dihydropyrimidinase-like 3 [Danio rerio] | 37.04 | 1.028 | 0.752 | 1.214 | 0.880 | 0.069 | 1.150 |
| gi|66392186 | dihydropyrimidinase-like 5 [Danio rerio] | 30.85 | 1.097 | 0.481 | 1.366 | 1.054 | 0.730 | 1.481 |
| gi|66472778 | dihydropyrimidinase-like 5b [Danio rerio] | 19.89 | 1.338 |  |  |  |  |  |
| gi|125743203 | DJ-1 [Carassius auratus] | 14.19 | 0.750 |  |  | 0.680 |  |  |
| gi|42406385 | Dlst protein [Danio rerio] | 8.53 | 0.892 | 0.446 | 1.687 | 1.060 | 0.235 | 1.160 |
| gi|92097700 | Dnl2 protein [Danio rerio] | 40.48 | 0.655 |  |  | 0.771 |  |  |
| gi|84627200 | dynein cytoplasmic 1 heavy chain 1 [Danio rerio] | 10.04 | 1.124 | 0.492 | 1.613 | 0.933 | 0.850 | 4.020 |
| gi|57526740 | dynein, light chain, LC8-type 2 [Danio rerio] | 38.20 | 1.001 |  |  | 1.260 |  |  |
| gi|7739753 | eIF2 alpha subunit [Danio rerio] | 16.51 | 0.616 |  |  | 0.666 |  |  |
| gi|55583970 | Elongation factor 1-gamma (EF-1-gamma) (eEF-1B gamma) | 3.62 |  |  |  |  |  |  |
| gi|3986186 | embryonic beta-type globin [Oncorhynchus mykiss] | 36.05 |  |  |  |  |  |  |
| gi|93115123 | enolase 1 alpha-like [Oreochromis mossambicus] | 43.60 |  |  |  |  |  |  |
| gi|48762657 | enolase 1, (alpha) [Danio rerio] | 50.23 | 0.897 |  |  | 1.385 |  |  |
| gi|51467931 | enolase 2 [Danio rerio] | 44.47 | 0.880 | 0.244 | 1.401 | 0.635 | 0.120 | 2.996 |
| gi|68086449 | Enolase 3, (beta, muscle) [Danio rerio] | 43.88 |  |  |  |  |  |  |
| gi|98979415 | enolase A [Acipenser baerii] | 50.00 |  |  |  |  |  |  |
| gi|98979417 | enolase A [Polypterus senegalus] | 50.00 |  |  |  |  |  |  |
| gi|52218912 | enoyl Coenzyme A hydratase, short chain, 1, mitochondrial [Danio rerio] | 12.71 |  |  |  |  |  |  |
| gi|585104 | Ependymin-2 precursor (Ependymin II) (EPD-II) | 40.00 | 1.261 | 0.550 | 31.653 | 1.335 | 0.421 | 17.399 |
| gi|41393175 | eukaryotic translation initiation factor 4A, isoform 1B [Danio rerio] | 9.11 |  |  |  |  |  |  |
| gi|48843356 | extracellular signal regulated protein kinase 2 [Cyprinus carpio] | 28.73 | 1.015 | 0.931 | 1.634 | 1.253 | 0.435 | 2.725 |
| gi|94732836 | family with sequence similarity 91, member A1 [Danio rerio] | 3.61 |  |  |  |  |  |  |
| gi|47085903 | far upstream element binding protein 1 [Danio rerio] | 4.21 | 1.126 |  |  |  |  |  |
| gi|409468 | fast myotomal muscle tropomyosin [Salmo salar] | 34.51 |  |  |  |  |  |  |
| gi|46849415 | fructose-bisphosphate aldolase A-2 [Acipenser baerii] | 46.53 |  |  |  |  |  |  |
| gi|1703244 | Fructose-bisphosphate aldolase C (Brain-type aldolase) | 70.25 | 0.979 | 0.825 | 1.221 | 0.869 | 0.098 | 1.186 |
| gi|46849403 | fructose-bisphosphate aldolase C [Amia calva] | 48.64 |  |  |  |  |  |  |
| gi|83320410 | gamma-aminobutyrate aminotransferase [Carassius auratus] | 17.46 |  |  |  |  |  |  |
| gi|56718619 | GAPDH [Danio rerio] | 61.49 | 0.943 | 0.791 | 9.004 | 0.641 | 0.205 | 6.583 |
| gi|304479 | gefiltin | 29.24 | 1.058 |  |  |  |  |  |
| gi|18858755 | gefiltin [Danio rerio] | 24.42 |  |  |  |  |  |  |
| gi|46329793 | Gfap protein [Danio rerio] | 52.60 | 0.196 |  |  | 0.127 |  |  |
| gi|46403243 | glutamate dehydrogenase 1 [Danio rerio] | 33.95 | 0.814 |  |  | 1.035 |  |  |
| gi|47086875 | glutamate dehydrogenase 1a [Danio rerio] | 26.29 | 0.892 |  |  | 1.409 |  |  |
| gi|41053395 | glutamate oxaloacetate transaminase 2 [Danio rerio] | 15.42 | 1.070 | 0.704 | 1.948 | 1.103 | 0.693 | 2.522 |
| gi|8132032 | glutamic acid decarboxylase isoform 67 [Carassius auratus] | 6.30 | 0.751 |  |  | 1.622 |  |  |
| gi|55508965 | glutamine synthetase [Carassius auratus] | 28.26 | 0.750 | 0.008 | 1.180 | 0.733 | 0.046 | 1.351 |
| gi|20799646 | glutamine synthetase [Oreochromis niloticus] | 17.79 | 1.115 | 0.391 | 1.369 | 0.737 | 0.225 | 1.892 |
| gi|47086689 | glutathione S-transferase M [Danio rerio] | 31.96 | 1.045 |  |  | 1.042 |  |  |
| gi|112901127 | glutathione S-transferase rho [Cyprinus carpio] | 36.28 | 0.860 | 0.039 | 1.151 | 0.791 | 0.049 | 1.263 |
| gi|55508980 | glyceraldehyde-3-phosphate dehydrogenase [Carassius auratus] | 54.29 | 0.794 | 0.062 | 1.282 | 0.730 | 0.009 | 1.179 |
| gi|24571224 | glyceraldehyde-3-phosphate dehydrogenase [Ctenopharyngodon idella] | 63.73 |  |  |  | 0.761 |  |  |
| gi|124300853 | glyceraldehyde-3-phosphate dehydrogenase [Solea senegalensis] | 47.76 |  |  |  |  |  |  |
| gi|94734233 | glycerol-3-phosphate dehydrogenase 1 (soluble) [Danio rerio] | 19.20 |  |  |  |  |  |  |
| gi|44662809 | glycoprotein M6Ba [Danio rerio] | 16.61 | 1.128 |  |  | 1.271 |  |  |
| gi|49899197 | Gmfb protein [Danio rerio] | 7.75 |  |  |  |  |  |  |
| gi|212954 | growth-associated protein GAP-43 | 29.11 | 1.070 | 0.322 | 1.249 | 0.835 | 0.725 | 145.618 |
| gi|41387190 | guanine nucleotide binding protein (G protein), alpha activating activity polypeptide O [Danio rerio] | 42.66 | 1.129 | 0.139 | 1.213 | 1.276 | 0.477 | 3.343 |
| gi|50370063 | Guanine nucleotide binding protein (G protein), alpha inhibiting activity polypeptide 2, like [Danio rerio] | 17.46 | 2.225 |  |  | 1.686 |  |  |
| gi|47087315 | guanine nucleotide binding protein (G protein), beta polypeptide 1, like [Danio rerio] | 30.00 |  |  |  |  |  |  |
| gi|49901135 | Guanine nucleotide binding protein (G protein), gamma 3 [Danio rerio] | 37.33 | 1.013 |  |  | 1.318 |  |  |
| gi|76445919 | guanine nucleotide binding protein G(o), alpha subunit 1 splice variant b [Salmo salar] | 37.94 |  |  |  |  |  |  |
| gi|41054141 | guanine nucleotide-binding protein Gi2 alpha-subunit [Danio rerio] | 23.34 |  |  |  |  |  |  |
| gi|77999572 | heat shock cognate 70 [Fundulus heteroclitus macrolepidotus] | 39.01 |  |  |  |  |  |  |
| gi|28569550 | heat shock cognate 70 kDa protein [Carassius auratus gibelio] | 39.29 | 1.107 |  |  | 0.889 |  |  |
| gi|229274 | hemoglobin alpha | 32.39 |  |  |  |  |  |  |
| gi|223199 | hemoglobin beta | 86.39 | 0.959 | 0.562 | 1.158 | 0.882 | 0.075 | 1.149 |
| gi|122370 | Hemoglobin subunit alpha (Hemoglobin alpha chain) (Alpha-globin) | 50.70 | 0.804 | 0.364 | 1.808 | 0.938 | 0.486 | 1.244 |
| gi|67972636 | heterogeneous nuclear ribonucleoprotein A/B [Danio rerio] | 15.29 | 0.393 |  |  | 0.544 |  |  |
| gi|47550715 | heterogeneous nuclear ribonucleoprotein A0 [Danio rerio] | 22.93 |  |  |  |  |  |  |
| gi|6840980 | hexokinase I [Cyprinus carpio] | 33.68 | 0.866 |  |  | 0.996 |  |  |
| gi|90103305 | high mobility group box 1-like [Ictalurus punctatus] | 12.09 | 0.960 |  |  | 0.811 |  |  |
| gi|82206956 | Histone H2AV (H2A.F/Z) | 32.81 | 0.916 | 0.392 | 1.324 | 0.895 | 0.655 | 2.039 |
| gi|112950077 | HSP60 [Carassius auratus] | 20.35 | 0.878 | 0.617 | 2.589 | 0.948 | 0.869 | 3.407 |
| gi|40807203 | Hsp90ab1 protein [Danio rerio] | 26.48 | 0.995 | 0.954 | 2.511 | 0.949 | 0.844 | 14.391 |
| gi|54400698 | hydroxysteroid (17-beta) dehydrogenase 10 [Danio rerio] | 5.77 |  |  |  |  |  |  |
| gi|47086749 | hypothetical protein LOC323529 [Danio rerio] | 25.57 |  |  |  |  |  |  |
| gi|41152406 | hypothetical protein LOC335335 [Danio rerio] | 39.81 | 0.979 | 0.905 | 1.697 | 0.834 | 0.422 | 1.861 |
| gi|41053909 | hypothetical protein LOC335836 [Danio rerio] | 20.14 | 0.909 | 0.446 | 1.548 | 0.995 | 0.983 | 2.315 |
| gi|41053339 | hypothetical protein LOC336637 [Danio rerio] | 32.41 | 0.662 | 0.231 | 7.361 | 0.431 | 0.576 | 813636.688 |
| gi|41054760 | hypothetical protein LOC393573 [Danio rerio] | 11.07 | 0.722 |  |  | 1.334 |  |  |
| gi|41055010 | hypothetical protein LOC393577 [Danio rerio] | 12.15 |  |  |  |  |  |  |
| gi|41152185 | hypothetical protein LOC393722 [Danio rerio] | 12.93 | 0.947 |  |  | 0.476 |  |  |
| gi|41055752 | hypothetical protein LOC393944 [Danio rerio] | 29.13 | 0.845 |  |  |  |  |  |
| gi|45387799 | hypothetical protein LOC402996 [Danio rerio] | 44.49 | 1.133 |  |  | 1.556 |  |  |
| gi|47086229 | hypothetical protein LOC405841 [Danio rerio] | 10.04 |  |  |  |  |  |  |
| gi|47086181 | hypothetical protein LOC405865 [Danio rerio] | 5.02 |  |  |  |  |  |  |
| gi|47085667 | hypothetical protein LOC406278 [Danio rerio] | 10.00 |  |  |  |  |  |  |
| gi|57526731 | hypothetical protein LOC406307 [Danio rerio] | 20.29 |  |  |  |  |  |  |
| gi|57526699 | hypothetical protein LOC406318 [Danio rerio] | 30.64 | 0.976 | 0.903 | 2.122 | 0.818 | 0.501 | 2.897 |
| gi|47085765 | hypothetical protein LOC406325 [Danio rerio] | 10.58 |  |  |  |  |  |  |
| gi|47085773 | hypothetical protein LOC406330 [Danio rerio] | 26.10 | 1.054 | 0.689 | 1.374 | 0.919 | 0.225 | 1.170 |
| gi|47085883 | hypothetical protein LOC406405 [Danio rerio] | 46.59 |  |  |  |  |  |  |
| gi|47086001 | hypothetical protein LOC406485 [Danio rerio] | 25.21 | 1.058 | 0.786 | 7.664 | 0.898 | 0.618 | 7.445 |
| gi|47086069 | hypothetical protein LOC406529 [Danio rerio] | 58.66 | 1.100 | 0.228 | 1.223 | 1.122 | 0.394 | 1.446 |
| gi|50344982 | hypothetical protein LOC415253 [Danio rerio] | 29.37 | 0.589 | 0.088 | 2.536 | 1.134 | 0.174 | 1.563 |
| gi|50539808 | hypothetical protein LOC436647 [Danio rerio] | 15.81 | 1.490 |  |  | 0.796 |  |  |
| gi|50539996 | hypothetical protein LOC436741 [Danio rerio] | 55.33 | 0.607 |  |  | 1.000 |  |  |
| gi|50540358 | hypothetical protein LOC436918 [Danio rerio] | 8.96 |  |  |  |  |  |  |
| gi|50540366 | hypothetical protein LOC436922 [Danio rerio] | 9.43 |  |  |  |  |  |  |
| gi|50540440 | hypothetical protein LOC436959 [Danio rerio] | 7.29 |  |  |  |  |  |  |
| gi|51010945 | hypothetical protein LOC445033 [Danio rerio] | 19.08 |  |  |  |  |  |  |
| gi|51230594 | hypothetical protein LOC445285 [Danio rerio] | 10.34 |  |  |  |  |  |  |
| gi|51571925 | hypothetical protein LOC445486 [Danio rerio] | 7.22 |  |  |  |  |  |  |
| gi|52218952 | hypothetical protein LOC447814 [Danio rerio] | 27.06 | 1.386 |  |  | 1.581 |  |  |
| gi|55925393 | hypothetical protein LOC492811 [Danio rerio] | 30.82 |  |  |  |  |  |  |
| gi|61806564 | hypothetical protein LOC541370 [Danio rerio] | 29.41 |  |  |  |  |  |  |
| gi|62122857 | hypothetical protein LOC541522 [Danio rerio] | 14.68 | 0.924 |  |  | 1.108 |  |  |
| gi|62955215 | hypothetical protein LOC550286 [Danio rerio] | 19.58 | 2.186 |  |  | 1.746 |  |  |
| gi|62955297 | hypothetical protein LOC550353 [Danio rerio] | 17.41 | 0.852 | 0.119 | 1.469 | 0.589 | 0.115 | 3.397 |
| gi|62955535 | hypothetical protein LOC550478 [Danio rerio] | 9.22 |  |  |  | 1.822 |  |  |
| gi|62955673 | hypothetical protein LOC550548 [Danio rerio] | 30.87 | 0.792 | 0.032 | 1.225 | 0.684 | 0.018 | 1.324 |
| gi|62955685 | hypothetical protein LOC550554 [Danio rerio] | 16.83 | 1.183 | 0.206 | 1.394 | 1.288 | 0.224 | 1.693 |
| gi|62955737 | hypothetical protein LOC550580 [Danio rerio] | 4.94 | 1.090 |  |  | 0.770 |  |  |
| gi|66472672 | hypothetical protein LOC553559 [Danio rerio] | 8.08 |  |  |  |  |  |  |
| gi|66472494 | hypothetical protein LOC553655 [Danio rerio] | 22.33 | 0.890 | 0.585 | 6.918 | 0.860 | 0.387 | 3.800 |
| gi|66472444 | hypothetical protein LOC553679 [Danio rerio] | 37.72 | 0.659 | 0.391 | 5.225 | 0.655 | 0.046 | 1.499 |
| gi|66472434 | hypothetical protein LOC553682 [Danio rerio] | 32.88 |  |  |  |  |  |  |
| gi|66472420 | hypothetical protein LOC553691 [Danio rerio] | 20.74 |  |  |  |  |  |  |
| gi|66773134 | hypothetical protein LOC554104 [Danio rerio] | 6.96 |  |  |  |  |  |  |
| gi|66773128 | hypothetical protein LOC554105 [Danio rerio] | 6.79 | 1.013 |  |  | 0.949 |  |  |
| gi|66773118 | hypothetical protein LOC554118 [Danio rerio] | 4.75 |  |  |  |  |  |  |
| gi|66773102 | hypothetical protein LOC554127 [Danio rerio] | 57.02 | 1.095 | 0.540 | 1.424 | 1.074 | 0.674 | 1.512 |
| gi|121582316 | hypothetical protein LOC555511 [Danio rerio] | 12.55 | 1.092 |  |  | 0.942 |  |  |
| gi|148232800 | hypothetical protein LOC557717 [Danio rerio] | 11.74 | 0.877 |  |  | 1.104 |  |  |
| gi|115494998 | hypothetical protein LOC558271 [Danio rerio] | 11.61 |  |  |  |  |  |  |
| gi|70887615 | hypothetical protein LOC558711 [Danio rerio] | 17.71 |  |  |  |  |  |  |
| gi|71834412 | hypothetical protein LOC559217 [Danio rerio] | 8.88 |  |  |  |  |  |  |
| gi|82658260 | hypothetical protein LOC560553 [Danio rerio] | 2.50 |  |  |  |  |  |  |
| gi|122114571 | hypothetical protein LOC562813 [Danio rerio] | 14.93 |  |  |  |  |  |  |
| gi|82658182 | hypothetical protein LOC564379 [Danio rerio] | 15.31 |  |  |  |  |  |  |
| gi|134133238 | hypothetical protein LOC569993 [Danio rerio] | 17.41 |  |  |  |  |  |  |
| gi|130486462 | hypothetical protein LOC570314 [Danio rerio] | 16.60 | 1.245 | 0.279 | 1.898 | 1.085 | 0.712 | 2.271 |
| gi|147905884 | hypothetical protein LOC573145 [Danio rerio] | 8.41 |  |  |  |  |  |  |
| gi|74315945 | hypothetical protein LOC613246 [Danio rerio] | 2.84 |  |  |  |  |  |  |
| gi|75750522 | hypothetical protein LOC619203 [Danio rerio] | 9.77 |  |  |  |  |  |  |
| gi|83025094 | hypothetical protein LOC641578 [Danio rerio] | 22.71 | 1.259 | 0.134 | 1.872 | 1.628 | 0.224 | 9.732 |
| gi|94536645 | hypothetical protein LOC678611 [Danio rerio] | 14.07 | 1.639 |  |  | 1.158 |  |  |
| gi|113679026 | hypothetical protein LOC751694 [Danio rerio] | 11.90 | 1.193 |  |  | 0.992 |  |  |
| gi|113679909 | hypothetical protein LOC751725 [Danio rerio] | 8.52 |  |  |  |  |  |  |
| gi|115529301 | hypothetical protein LOC767746 [Danio rerio] | 57.66 | 0.815 |  |  |  |  |  |
| gi|116268041 | hypothetical protein LOC768183 [Danio rerio] | 4.36 |  |  |  |  |  |  |
| gi|118150474 | hypothetical protein LOC777641 [Danio rerio] | 20.24 |  |  |  |  |  |  |
| gi|123701145 | hypothetical protein LOC791220 [Danio rerio] | 27.80 | 1.014 |  |  | 0.824 |  |  |
| gi|51467938 | Iglon1 [Danio rerio] | 8.38 | 0.870 |  |  | 0.641 |  |  |
| gi|49618965 | immunoglobulin binding protein [Danio rerio] | 15.85 | 0.812 | 0.149 | 1.879 | 0.965 | 0.933 | 73.334 |
| gi|124740 | Intermediate filament protein ON3 | 46.73 | 1.260 | 0.194 | 1.510 | 1.093 | 0.258 | 1.196 |
| gi|41393155 | isocitrate dehydrogenase 1 (NADP+), soluble [Danio rerio] | 33.57 | 0.738 | 0.333 | 9.218 | 0.839 | 0.732 | 147.772 |
| gi|41054651 | isocitrate dehydrogenase 2 (NADP+), mitochondrial [Danio rerio] | 45.21 | 1.229 |  |  | 0.820 |  |  |
| gi|46358344 | isocitrate dehydrogenase 3 (NAD+) alpha [Danio rerio] | 6.85 | 0.838 |  |  | 1.100 |  |  |
| gi|515481 | kainate receptor alpha subunit | 28.54 | 1.325 | 0.001 | 1.125 | 1.823 | 0.000 | 1.222 |
| gi|212997 | keratin | 29.66 | 1.031 |  |  | 0.683 |  |  |
| gi|41351240 | Keratin 18 [Danio rerio] | 36.66 | 0.752 |  |  | 0.925 |  |  |
| gi|32452095 | keratin type I [Acipenser baerii] | 25.66 | 4.101 | 0.228 | 818.796 | 6.623 |  |  |
| gi|3183052 | Keratin, type I cytoskeletal 50 kDa (GK50) | 31.91 | 0.980 | 0.937 | 1.915 | 0.830 | 0.459 | 2.015 |
| gi|39645432 | Krt5 protein [Danio rerio] | 42.50 | 2.636 | 0.022 | 1.543 | 2.863 |  |  |
| gi|56207279 | lactate dehydrogenase B4 [Danio rerio] | 34.43 | 0.996 | 0.974 | 1.469 | 0.930 | 0.560 | 1.421 |
| gi|115343222 | lactate dehydrogenase isoform A [Misgurnus fossilis] | 29.13 | 0.839 | 0.081 | 1.233 | 0.878 | 0.408 | 1.477 |
| gi|20386748 | liver-basic fatty acid binding protein [Acanthopagrus schlegelii] | 32.79 | 3.623 | 0.026 | 2.468 | 8.561 | 0.015 | 3.158 |
| gi|37590410 | LOC402880 protein [Danio rerio] | 16.37 |  |  |  |  |  |  |
| gi|92096413 | LOC569631 protein [Danio rerio] | 37.96 | 0.849 | 0.531 | 1.832 | 0.783 | 0.248 | 1.650 |
| gi|134026424 | macrophage migration inhibitory factor [Danio rerio] | 26.96 | 0.684 |  |  | 0.483 |  |  |
| gi|41053939 | malate dehydrogenase 1a, NAD (soluble) [Danio rerio] | 35.08 | 1.064 | 0.262 | 1.128 | 1.167 | 0.048 | 1.166 |
| gi|57525624 | malic enzyme 2, NAD(+)-dependent, mitochondrial [Danio rerio] | 10.50 |  |  |  |  |  |  |
| gi|47085887 | MARCKS-like 1 [Danio rerio] | 38.97 |  |  |  |  |  |  |
| gi|68084823 | Methylthioadenosine phosphorylase [Danio rerio] | 12.50 |  |  |  |  |  |  |
| gi|14009437 | mitochondrial ATP synthase alpha-subunit [Cyprinus carpio] | 38.59 |  |  |  |  |  |  |
| gi|82182691 | Mitochondrial import inner membrane translocase subunit Tim8 A | 12.22 |  |  |  |  |  |  |
| gi|93115142 | mitochondrial isocitrate dehydrogenase 2-like [Oreochromis mossambicus] | 45.58 | 1.074 | 0.077 | 1.117 | 0.933 | 0.570 | 2.988 |
| gi|55250893 | Myelin protein zero [Danio rerio] | 16.75 | 0.842 |  |  | 0.309 |  |  |
| gi|85067845 | myoglobin isoform 2 [Cyprinus carpio] | 45.58 | 0.817 | 0.150 | 1.334 | 0.719 | 0.031 | 1.340 |
| gi|62632723 | myristoylated alanine rich protein kinase C substrate [Danio rerio] | 37.00 | 0.469 | 0.209 | 26.590 |  |  |  |
| gi|47086029 | myristoylated alanine-rich C kinase substrate 2 [Danio rerio] | 38.65 | 1.110 |  |  | 0.776 |  |  |
| gi|9789571 | Na+/K+ ATPase alpha subunit isoform 1 [Danio rerio] | 46.69 | 0.989 | 0.874 | 1.300 | 1.022 | 0.532 | 1.134 |
| gi|11067030 | Na+/K+ ATPase alpha subunit isoform 6 [Danio rerio] | 49.95 | 0.929 | 0.452 | 2.249 | 0.717 | 0.172 | 3.232 |
| gi|3399699 | natural killer cell enhancing factor [Cyprinus carpio] | 39.70 | 1.045 | 0.842 | 9.231 | 1.211 | 0.053 | 1.223 |
| gi|41393137 | N-ethylmaleimide-sensitive factor [Danio rerio] | 26.61 | 1.283 | 0.006 | 1.173 | 1.264 | 0.073 | 1.300 |
| gi|18859063 | neural cell adhesion molecule 1 [Danio rerio] | 11.81 | 0.850 |  |  | 0.799 |  |  |
| gi|47550793 | nicotinamide nucleotide transhydrogenase [Danio rerio] | 6.95 | 1.345 | 0.603 | 186.986 | 1.157 |  |  |
| gi|94734440 | novel protein (zgc:55440) [Danio rerio] | 2.96 |  |  |  |  |  |  |
| gi|94732569 | novel protein (zgc:55919) [Danio rerio] | 43.08 | 1.427 | 0.199 | 4.303 | 1.270 | 0.223 | 3.044 |
| gi|94733342 | novel protein (zgc:56546) [Danio rerio] | 25.57 |  |  |  |  |  |  |
| gi|94733356 | novel protein (zgc:56557) [Danio rerio] | 10.25 | 2.027 |  |  |  |  |  |
| gi|122890758 | novel protein (zgc:63516) [Danio rerio] | 37.44 |  |  |  |  |  |  |
| gi|94732272 | novel protein (zgc:73093) [Danio rerio] | 37.30 |  |  |  |  |  |  |
| gi|55251344 | novel protein (zgc:73360) [Danio rerio] | 21.98 | 0.831 |  |  | 0.660 |  |  |
| gi|126635172 | novel protein [Danio rerio] | 7.38 | 1.431 |  |  | 1.049 |  |  |
| gi|148724895 | novel protein containing an ATP synthase E chain domain (zgc:153661) [Danio rerio] | 33.80 | 0.926 |  |  | 0.919 |  |  |
| gi|56207786 | novel protein similar to heat shock protein 90-alpha (hsp90a) [Danio rerio] | 27.66 |  |  |  |  |  |  |
| gi|33284915 | novel protein similar to human catenin (cadherin-associated protein), alpha 2 (CTNNA2) [Danio rerio] | 9.97 |  |  |  |  |  |  |
| gi|55962784 | novel protein similar to microtubule-associated protein, RP/EB family [Danio rerio] | 9.06 |  |  |  |  |  |  |
| gi|148725808 | novel protein similar to vertebrate adenylate cyclase family [Danio rerio] | 7.64 |  |  |  |  |  |  |
| gi|56207852 | novel protein similar to vertebrate aldo-keto reductase family 1, member B1 (aldose reducatse) (AKR1B1) [Danio rerio] | 12.06 | 0.912 | 0.458 | 2.786 | 0.789 | 0.359 | 6.748 |
| gi|56207502 | novel protein similar to vertebrate ATPase, H+ transporting, lysosomal 14kDa, V1 subunit F (ATP6V1F) [Danio rerio] | 17.65 | 0.924 | 0.174 | 1.327 | 1.139 | 0.071 | 1.202 |
| gi|94732992 | novel protein similar to vertebrate calcium channel, voltage-dependent, alpha 2/delta subunit 2 (CACNA2D2) [Danio rerio] | 4.07 |  |  |  |  |  |  |
| gi|123227825 | novel protein similar to vertebrate calcium/calmodulin-dependent protein kinase (CaM kinase) II gamma (CAMK2G) [Danio rerio] | 27.67 |  |  |  |  |  |  |
| gi|148726424 | novel protein similar to vertebrate dynamin family [Danio rerio] | 24.85 | 1.261 |  |  | 1.720 |  |  |
| gi|94733316 | novel protein similar to vertebrate dynein, cytoplasmic, light intermediate polypeptide 2 (DNCLI2) [Danio rerio] | 7.39 |  |  |  |  |  |  |
| gi|148726029 | novel protein similar to vertebrate EF hand calcium binding protein 2 (EFCBP2, zgc:112232) [Danio rerio] | 22.90 | 0.891 | 0.036 | 1.087 | 0.843 | 0.319 | 3.279 |
| gi|122891007 | novel protein similar to vertebrate endosulfine alpha (ENSA) [Danio rerio] | 14.53 |  |  |  |  |  |  |
| gi|94734068 | novel protein similar to vertebrate guanine nucleotide binding protein (G protein), q polypeptide (GNAQ) [Danio rerio] | 11.75 |  |  |  |  |  |  |
| gi|94733986 | novel protein similar to vertebrate kalirin, RhoGEF kinase (KALRN) [Danio rerio] | 2.64 |  |  |  |  |  |  |
| gi|148726003 | novel protein similar to vertebrate plectin 1, intermediate filament binding protein 500kDa (PLEC1) [Danio rerio] | 5.00 |  |  |  |  |  |  |
| gi|56207862 | novel protein similar to vertebrate proteasome (prosome, macropain) 26S subunit, ATPase, 2 (PSMC2) (zgc:63995) [Danio rerio] | 15.01 | 1.395 |  |  | 1.259 |  |  |
| gi|94733821 | novel protein similar to vertebrate ribosomal protein S16 (RPS16) [Danio rerio] | 19.18 | 0.886 | 0.077 | 1.205 | 1.058 | 0.276 | 1.396 |
| gi|148726431 | novel protein similar to vertebrate solute carrier family 4, sodium bicarbonate cotransporter, member 4 (SLC4A4) [Danio rerio] | 3.68 | 1.317 |  |  | 1.434 |  |  |
| gi|148726372 | novel protein similar to vertebrate spectrin, beta, non-erythrocytic 2 (SPTBN2) [Danio rerio] | 11.50 | 1.029 | 0.899 | 1.691 | 1.263 | 0.167 | 1.468 |
| gi|55251299 | novel protein similar to vertebrate synaptotagmin I (SYT1) [Danio rerio] | 23.87 | 1.123 | 0.465 | 1.556 | 0.947 | 0.789 | 2.157 |
| gi|56207931 | novel protein similar to vertebratesecretory granule, neuroendocrine protein 1 (7B2 protein) (SGNE1) [Danio rerio] | 7.31 |  |  |  |  |  |  |
| gi|55925383 | NSFL1 (p97) cofactor (p47) [Danio rerio] | 5.65 |  |  |  |  |  |  |
| gi|60729607 | nucleolin - common carp | 18.76 |  |  |  |  |  |  |
| gi|33359633 | nucleolin 2 [Cyprinus carpio] | 18.66 |  |  |  |  |  |  |
| gi|17977825 | parvalbumin [Cyprinus carpio] | 44.04 |  |  |  |  |  |  |
| gi|17977827 | parvalbumin [Cyprinus carpio] | 44.95 |  |  |  |  |  |  |
| gi|94733617 | parvalbumin [Danio rerio] | 74.31 | 0.875 | 0.001 | 1.050 | 0.944 | 0.514 | 1.224 |
| gi|71152827 | pcp4a [Danio rerio] | 74.60 | 0.803 | 0.367 | 6.157 | 1.063 | 0.773 | 8.030 |
| gi|68161039 | peptidylprolyl isomerase A [Ictalurus punctatus] | 42.07 |  |  |  |  |  |  |
| gi|85719973 | peptidylprolyl isomerase B [Ictalurus punctatus] | 16.67 |  |  |  |  |  |  |
| gi|41351079 | Phb protein [Danio rerio] | 21.40 | 1.623 |  |  | 1.379 |  |  |
| gi|52218996 | phosphofructokinase, muscle [Danio rerio] | 5.23 |  |  |  |  |  |  |
| gi|41056111 | phosphoglucomutase 1 [Danio rerio] | 22.64 |  |  |  |  |  |  |
| gi|47087205 | phosphogluconate hydrogenase isoform 2 [Danio rerio] | 10.14 | 0.823 |  |  | 0.979 |  |  |
| gi|46849395 | phosphoglycerate kinase [Lepisosteus osseus] | 31.62 | 1.316 | 0.334 | 2.553 | 1.063 | 0.774 | 2.234 |
| gi|47087077 | phosphoglycerate kinase 1 [Danio rerio] | 43.17 | 1.069 | 0.693 | 1.629 | 0.898 | 0.486 | 3.696 |
| gi|29648610 | phospholipid hydroperoxide glutathione peroxidase B [Danio rerio] | 39.64 | 0.934 | 0.649 | 1.748 | 1.325 |  |  |
| gi|95832164 | Pi-class glutathione S-transferase [Carassius auratus] | 55.29 | 0.610 | 0.016 | 1.373 | 0.573 | 0.003 | 1.216 |
| gi|112901122 | Pi-class glutathione S-transferase [Cyprinus carpio] | 48.56 | 0.708 |  |  | 0.870 |  |  |
| gi|45501385 | Pkm2 protein [Danio rerio] | 42.48 |  |  |  |  |  |  |
| gi|4996224 | polypeptide elongation factor 1 alpha [Oryzias latipes] | 15.84 | 1.307 | 0.219 | 1.632 | 1.282 | 0.014 | 1.179 |
| gi|71682330 | Ppia protein [Danio rerio] | 44.51 | 0.889 | 0.367 | 2.640 | 0.877 |  |  |
| gi|125829043 | PREDICTED: aminopeptidase puromycin sensitive [Danio rerio] | 6.41 |  |  |  |  |  |  |
| gi|68437695 | PREDICTED: hypothetical protein [Danio rerio] | 32.46 | 1.218 |  |  | 1.309 |  |  |
| gi|125864153 | PREDICTED: hypothetical protein [Danio rerio] | 26.12 |  |  |  |  |  |  |
| gi|125854907 | PREDICTED: hypothetical protein [Danio rerio] | 28.21 | 1.067 | 0.756 | 7.798 | 0.878 | 0.322 | 2.494 |
| gi|68353760 | PREDICTED: hypothetical protein [Danio rerio] | 38.98 | 1.015 | 0.838 | 1.192 | 0.718 | 0.239 | 2.053 |
| gi|68361734 | PREDICTED: hypothetical protein [Danio rerio] | 39.91 | 1.017 | 0.956 | 2.477 | 0.939 | 0.741 | 1.743 |
| gi|125823046 | PREDICTED: hypothetical protein [Danio rerio] | 21.92 | 1.121 | 0.727 | 2.572 | 0.679 | 0.313 | 3.476 |
| gi|125805843 | PREDICTED: hypothetical protein [Danio rerio] | 8.70 | 0.699 |  |  | 0.712 |  |  |
| gi|125854701 | PREDICTED: hypothetical protein [Danio rerio] | 5.29 | 1.426 | 0.009 | 1.234 | 1.224 | 0.193 | 1.431 |
| gi|125836073 | PREDICTED: hypothetical protein [Danio rerio] | 19.02 | 0.657 |  |  | 1.114 | 0.868 | 685.287 |
| gi|125826331 | PREDICTED: hypothetical protein [Danio rerio] | 36.91 | 0.912 | 0.091 | 1.138 | 0.960 | 0.712 | 1.508 |
| gi|125851563 | PREDICTED: hypothetical protein [Danio rerio] | 20.23 | 1.114 |  |  | 1.103 |  |  |
| gi|125840569 | PREDICTED: hypothetical protein [Danio rerio] | 6.14 | 1.466 |  |  | 1.237 |  |  |
| gi|68436373 | PREDICTED: hypothetical protein [Danio rerio] | 18.15 | 1.205 | 0.230 | 1.598 | 1.184 | 0.265 | 1.609 |
| gi|125816311 | PREDICTED: hypothetical protein [Danio rerio] | 9.53 | 1.096 | 0.514 | 1.647 | 1.314 | 0.223 | 3.553 |
| gi|68374333 | PREDICTED: hypothetical protein [Danio rerio] | 9.39 |  |  |  |  |  |  |
| gi|125840959 | PREDICTED: hypothetical protein [Danio rerio] | 17.18 | 0.800 |  |  | 0.658 |  |  |
| gi|125818653 | PREDICTED: hypothetical protein [Danio rerio] | 5.27 |  |  |  |  |  |  |
| gi|125818422 | PREDICTED: hypothetical protein [Danio rerio] | 13.64 | 1.036 | 0.838 | 1.573 | 0.907 | 0.407 | 1.341 |
| gi|125826878 | PREDICTED: hypothetical protein [Danio rerio] | 3.72 |  |  |  |  |  |  |
| gi|125817367 | PREDICTED: hypothetical protein [Danio rerio] | 3.94 |  |  |  |  |  |  |
| gi|125827820 | PREDICTED: hypothetical protein [Danio rerio] | 16.25 |  |  |  |  |  |  |
| gi|68357754 | PREDICTED: hypothetical protein [Danio rerio] | 13.29 |  |  |  |  |  |  |
| gi|68391583 | PREDICTED: hypothetical protein [Danio rerio] | 10.85 |  |  |  |  |  |  |
| gi|125805727 | PREDICTED: hypothetical protein [Danio rerio] | 11.14 |  |  |  |  |  |  |
| gi|125803682 | PREDICTED: hypothetical protein [Danio rerio] | 4.79 |  |  |  |  |  |  |
| gi|125829846 | PREDICTED: hypothetical protein [Danio rerio] | 7.45 |  |  |  |  |  |  |
| gi|125812732 | PREDICTED: hypothetical protein [Danio rerio] | 30.77 |  |  |  |  |  |  |
| gi|125852449 | PREDICTED: hypothetical protein [Danio rerio] | 5.64 | 1.083 |  |  | 0.429 |  |  |
| gi|125824635 | PREDICTED: hypothetical protein [Danio rerio] | 5.68 |  |  |  |  |  |  |
| gi|125825483 | PREDICTED: hypothetical protein [Danio rerio] | 1.79 | 0.630 |  |  | 1.490 |  |  |
| gi|125819459 | PREDICTED: hypothetical protein [Danio rerio] | 14.68 |  |  |  |  |  |  |
| gi|125851497 | PREDICTED: hypothetical protein [Danio rerio] | 5.51 |  |  |  |  |  |  |
| gi|68359747 | PREDICTED: hypothetical protein [Danio rerio] | 51.31 |  |  |  |  |  |  |
| gi|125845769 | PREDICTED: hypothetical protein [Danio rerio] | 4.37 |  |  |  |  |  |  |
| gi|68380717 | PREDICTED: hypothetical protein isoform 1 [Danio rerio] | 20.58 |  |  |  |  |  |  |
| gi|125814548 | PREDICTED: hypothetical protein isoform 1 [Danio rerio] | 45.38 | 0.840 | 0.478 | 2.381 | 0.799 | 0.574 | 4.275 |
| gi|68438381 | PREDICTED: hypothetical protein isoform 1 [Danio rerio] | 4.27 |  |  |  |  |  |  |
| gi|68369456 | PREDICTED: hypothetical protein isoform 1 [Danio rerio] | 6.23 |  |  |  |  |  |  |
| gi|125829936 | PREDICTED: hypothetical protein isoform 2 [Danio rerio] | 11.32 |  |  |  |  |  |  |
| gi|125811718 | PREDICTED: hypothetical protein isoform 3 [Danio rerio] | 24.44 | 2.327 |  |  |  |  |  |
| gi|68374463 | PREDICTED: hypothetical protein isoform 3 [Danio rerio] | 4.63 |  |  |  |  |  |  |
| gi|125820106 | PREDICTED: hypothetical protein LOC323055 [Danio rerio] | 29.84 | 1.061 |  |  | 1.076 |  |  |
| gi|125816973 | PREDICTED: hypothetical protein LOC336168 [Danio rerio] | 4.55 | 1.022 | 0.937 | 15.477 | 1.060 |  |  |
| gi|125844880 | PREDICTED: myelin basic protein isoform 3 [Danio rerio] | 28.82 | 0.588 | 0.082 | 2.006 | 0.256 | 0.003 | 1.397 |
| gi|125863722 | PREDICTED: similar to 1-phosphatidylinositol-4,5-bisphosphate phosphodiesterase (EC 3.1.4.11) beta-1b - rat, partial [Danio rerio] | 17.07 |  |  |  |  |  |  |
| gi|125846042 | PREDICTED: similar to 26S protease regulatory subunit S10B [Danio rerio] | 14.72 |  |  |  |  |  |  |
| gi|125817179 | PREDICTED: similar to adaptor protein X11alpha [Danio rerio] | 2.17 |  |  |  |  |  |  |
| gi|125834993 | PREDICTED: similar to AHNAK nucleoprotein [Danio rerio] | 17.96 | 0.922 | 0.402 | 1.235 | 0.852 | 0.087 | 1.208 |
| gi|125815934 | PREDICTED: similar to beta-spectrin [Danio rerio] | 37.38 | 1.018 |  |  | 1.442 |  |  |
| gi|125834151 | PREDICTED: similar to calcium/calmodulin-dependent protein kinase II isoform gamma-C protein kinase II isoform 2 [Danio rerio] | 24.78 |  |  |  |  |  |  |
| gi|125840525 | PREDICTED: similar to clathrin assembly protein AP180 [Danio rerio] | 7.91 |  |  |  |  |  |  |
| gi|125837195 | PREDICTED: similar to clathrin, heavy polypeptide (Hc), [Danio rerio] | 17.82 | 0.947 | 0.749 | 5.186 | 1.084 | 0.808 | 26.641 |
| gi|68354162 | PREDICTED: similar to collapsin response mediator protein 1 isoform 1 [Danio rerio] | 15.28 | 0.668 |  |  | 0.729 |  |  |
| gi|68402425 | PREDICTED: similar to GDP dissociation inhibitor 1 [Danio rerio] | 39.15 | 1.182 | 0.348 | 1.809 | 1.206 | 0.339 | 4.068 |
| gi|125819285 | PREDICTED: similar to guanine nucleotide-binding protein G-s-alpha-2 isoform 1 [Danio rerio] | 12.41 |  |  |  |  |  |  |
| gi|125838086 | PREDICTED: similar to hCG32806, [Danio rerio] | 5.04 |  |  |  |  |  |  |
| gi|125817375 | PREDICTED: similar to heterotrimeric guanine nucleotide-binding protein alpha q subunit isoform 1 [Danio rerio] | 13.80 |  |  |  |  |  |  |
| gi|125808649 | PREDICTED: similar to histone protein Hist2h3c1 [Danio rerio] | 18.88 | 1.058 | 0.683 | 1.381 | 1.103 | 0.526 | 1.446 |
| gi|125826558 | PREDICTED: similar to KIAA0587 protein isoform 2 [Danio rerio] | 7.98 | 1.057 |  |  | 1.615 |  |  |
| gi|125864155 | PREDICTED: similar to LOC553451 protein [Danio rerio] | 14.75 |  |  |  |  |  |  |
| gi|125834064 | PREDICTED: similar to MGC145965 protein [Danio rerio] | 20.00 |  |  |  |  |  |  |
| gi|125827508 | PREDICTED: similar to MGC81043 protein [Danio rerio] | 12.13 | 1.336 |  |  | 1.362 | 0.516 | 62.604 |
| gi|125805242 | PREDICTED: similar to MGC84000 protein [Danio rerio] | 24.12 |  |  |  |  |  |  |
| gi|125812189 | PREDICTED: similar to microtubule-associated protein tau, [Danio rerio] | 18.60 | 0.993 | 0.422 | 1.068 | 0.918 | 0.040 | 1.071 |
| gi|125819055 | PREDICTED: similar to mutant Ca2+-dependent secretion activator [Danio rerio] | 6.13 |  |  |  |  |  |  |
| gi|125838862 | PREDICTED: similar to Myelin basic protein [Danio rerio] | 15.70 | 0.664 | 0.012 | 1.100 | 0.664 | 0.024 | 1.219 |
| gi|68441061 | PREDICTED: similar to neurocalcin [Danio rerio] | 47.15 | 1.204 |  |  | 0.877 |  |  |
| gi|125847831 | PREDICTED: similar to neurofilament 3, medium [Danio rerio] | 12.96 |  |  |  |  |  |  |
| gi|68433167 | PREDICTED: similar to neurogranin [Danio rerio] | 51.67 | 0.623 | 0.443 | 150.390 | 1.747 |  |  |
| gi|125836578 | PREDICTED: similar to Nj-synaphin 2 [Danio rerio] | 32.30 | 0.448 | 0.002 | 1.258 | 0.640 | 0.090 | 1.857 |
| gi|125864151 | PREDICTED: similar to non-erythrocyte beta spectrin [Danio rerio] | 28.72 | 0.781 | 0.353 | 2.426 | 0.963 | 0.922 | 4.374 |
| gi|125833659 | PREDICTED: similar to non-muscle alpha-actinin 1 isoform 4 [Danio rerio] | 10.02 |  |  |  |  |  |  |
| gi|125818121 | PREDICTED: similar to nonmuscle myosin heavy chain [Danio rerio] | 8.22 |  |  |  |  |  |  |
| gi|125814138 | PREDICTED: similar to novel proein similar to vertebrate synaptosomal-associated protein (SNAP91) [Danio rerio] | 14.59 | 0.909 | 0.258 | 1.243 | 0.999 | 0.995 | 1.514 |
| gi|125810029 | PREDICTED: similar to OTTHUMP00000028706, partial [Danio rerio] | 3.69 |  |  |  |  |  |  |
| gi|125851990 | PREDICTED: similar to plasma membrane calcium ATPase [Danio rerio] | 8.94 |  |  |  |  |  |  |
| gi|125825989 | PREDICTED: similar to polyprotein [Danio rerio] | 2.87 |  |  |  |  |  |  |
| gi|125840883 | PREDICTED: similar to Proteasome (prosome, macropain) 26S subunit, ATPase, 4 [Danio rerio] | 5.98 | 1.513 |  |  | 1.212 |  |  |
| gi|68372225 | PREDICTED: similar to Pur-alpha [Danio rerio] | 14.34 |  |  |  |  |  |  |
| gi|125834961 | PREDICTED: similar to RAPGEF2 protein [Danio rerio] | 5.26 |  |  |  |  |  |  |
| gi|125828955 | PREDICTED: similar to Solute carrier family 4, sodium bicarbonate cotransporter-like, member 10 [Danio rerio] | 6.84 |  |  |  |  |  |  |
| gi|125824530 | PREDICTED: similar to synapsin Ia [Danio rerio] | 21.43 | 1.356 | 0.177 | 1.647 | 1.325 | 0.057 | 1.342 |
| gi|125850082 | PREDICTED: similar to Transmembrane protein 163 [Danio rerio] | 5.68 |  |  |  |  |  |  |
| gi|125818111 | PREDICTED: similar to tubulin alpha 6 [Danio rerio] | 60.31 | 0.872 | 0.292 | 2.357 | 1.117 |  |  |
| gi|125832860 | PREDICTED: similar to Xm278-prov protein [Danio rerio] | 28.26 | 1.319 | 0.232 | 3.832 | 1.324 | 0.427 | 16.987 |
| gi|68380112 | PREDICTED: v-crk sarcoma virus CT10 oncogene homolog isoform 1 [Danio rerio] | 8.36 |  |  |  |  |  |  |
| gi|62578 | pre-proependymin I (AA -2 to 195) [Carassius auratus] | 57.41 | 0.668 | 0.111 | 1.775 | 0.860 | 0.224 | 1.371 |
| gi|56118638 | profilin 2 like [Danio rerio] | 28.78 | 1.065 | 0.727 | 5.736 | 1.021 |  |  |
| gi|57526509 | propionyl-Coenzyme A carboxylase, alpha polypeptide [Danio rerio] | 4.09 |  |  |  |  |  |  |
| gi|50540284 | proteasome (prosome, macropain) subunit, beta type, 2 [Danio rerio] | 19.10 |  |  |  |  |  |  |
| gi|51242139 | proteasome (prosome, macropain) subunit, beta type, 3 [Danio rerio] | 18.05 | 0.913 | 0.373 | 2.158 | 0.825 |  |  |
| gi|41152183 | protein (peptidyl-prolyl cis/trans isomerase) NIMA-interacting 1 [Danio rerio] | 12.58 | 0.828 |  |  | 1.049 |  |  |
| gi|85719991 | protein disulfide isomerase-related protein P5 precursor [Ictalurus punctatus] | 12.05 |  |  |  |  |  |  |
| gi|82180540 | Protein DJ-1 (zDJ-1) (Parkinson disease protein 7 homolog) | 17.46 |  |  |  |  |  |  |
| gi|82179750 | Protein NDRG2 | 10.87 | 1.009 |  |  | 0.793 |  |  |
| gi|78100734 | Protein NipSnap2 (Glioblastoma amplified sequence) | 3.15 |  |  |  |  |  |  |
| gi|148725496 | protein phosphatase 2 (formerly 2A), regulatory subunit A, beta isoform [Danio rerio] | 13.75 | 0.872 | 0.226 | 1.909 | 0.979 | 0.825 | 2.586 |
| gi|47271364 | protein phosphatase type 2C alpha 2 [Danio rerio] | 8.59 | 0.884 |  |  | 1.332 |  |  |
| gi|388623 | putative | 55.43 | 0.303 |  |  | 0.336 |  |  |
| gi|10180968 | putative oncoprotein nm23 [Ictalurus punctatus] | 51.63 | 0.781 | 0.343 | 2.367 | 0.772 | 0.039 | 1.256 |
| gi|51859586 | Pvalb6 protein [Danio rerio] | 57.80 | 0.949 |  |  | 0.559 |  |  |
| gi|18858695 | pyruvate carboxylase [Danio rerio] | 14.75 | 0.986 |  |  | 1.416 |  |  |
| gi|24940582 | pyruvate carboxylase [Pagrus major] | 14.33 |  |  |  |  |  |  |
| gi|53749653 | pyruvate dehydrogenase E1 alpha 1 [Danio rerio] | 13.99 | 1.074 | 0.813 | 2.425 | 0.920 | 0.586 | 1.545 |
| gi|40786398 | pyruvate kinase, muscle [Danio rerio] | 39.85 | 0.476 |  |  | 0.680 |  |  |
| gi|55742328 | RAB1A, member RAS oncogene family [Danio rerio] | 25.37 |  |  |  |  |  |  |
| gi|41393075 | RAB2A, member RAS oncogene family [Danio rerio] | 14.15 | 1.105 |  |  | 1.064 |  |  |
| gi|62955495 | RAB3A, member RAS oncogene family [Danio rerio] | 23.64 | 0.840 | 0.678 | 54.949 | 1.038 | 0.770 | 3.541 |
| gi|94733793 | RAB5A, member RAS oncogene family [Danio rerio] | 20.83 |  |  |  |  |  |  |
| gi|41393159 | RAB5C, member RAS oncogene family [Danio rerio] | 35.75 |  |  |  |  |  |  |
| gi|51972166 | radixin isoform 1 [Danio rerio] | 7.43 |  |  |  |  |  |  |
| gi|94732736 | ras homolog gene family, member Aa [Danio rerio] | 32.64 | 1.117 | 0.139 | 1.220 | 0.894 | 0.277 | 1.383 |
| gi|56207811 | RAS related protein 1b [Danio rerio] | 33.70 |  |  |  |  |  |  |
| gi|57526488 | ras-related C3 botulinum toxin substrate 3 (rho family, small GTP binding protein Rac3) [Danio rerio] | 34.90 | 1.179 | 0.748 | 151.869 | 0.841 | 0.171 | 1.834 |
| gi|62719420 | reggie protein 1a [Takifugu rubripes] | 8.96 | 1.261 |  |  | 1.006 |  |  |
| gi|62955603 | related RAS viral (r-ras) oncogene homolog 2 [Danio rerio] | 23.27 |  |  |  |  |  |  |
| gi|72535156 | reticulon 1a isoform 1 [Danio rerio] | 15.04 | 1.321 | 0.320 | 2.107 | 1.223 | 0.243 | 1.555 |
| gi|55251317 | reticulon 3 [Danio rerio] | 13.12 |  |  |  |  |  |  |
| gi|77416569 | Reticulon-4-interacting protein 1 homolog, mitochondrial precursor | 8.01 |  |  |  |  |  |  |
| gi|71051661 | Ribosomal protein L23a [Danio rerio] | 15.48 | 1.091 | 0.813 | 4.060 | 1.278 | 0.476 | 3.364 |
| gi|52430376 | ribosomal protein L27 [Fundulus heteroclitus] | 7.44 | 1.318 |  |  | 1.828 |  |  |
| gi|15293941 | ribosomal protein L36 [Ictalurus punctatus] | 10.48 |  |  |  |  |  |  |
| gi|94733359 | ribosomal protein L4 [Danio rerio] | 8.00 |  |  |  |  |  |  |
| gi|15293875 | ribosomal protein L6 [Ictalurus punctatus] | 11.92 | 1.379 |  |  | 1.441 |  |  |
| gi|124300825 | ribosomal protein S19 [Solea senegalensis] | 23.81 | 1.120 | 0.388 | 2.721 | 1.090 | 0.476 | 2.769 |
| gi|47086117 | ribosomal protein S2 [Danio rerio] | 25.36 |  |  |  |  |  |  |
| gi|74136127 | rpL14 protein [Takifugu rubripes] | 16.06 |  |  |  |  |  |  |
| gi|37779084 | S6 ribosomal protein [Pagrus major] | 20.32 |  |  |  |  |  |  |
| gi|41055754 | sarcolemma associated protein [Danio rerio] | 11.17 | 1.116 | 0.379 | 1.526 | 1.042 | 0.598 | 2.043 |
| gi|27881963 | Sb:cb825 protein [Danio rerio] | 19.72 | 1.340 | 0.281 | 5.805 | 1.453 | 0.438 | 49.822 |
| gi|94734332 | SEC13-like 1 (S. cerevisiae) [Danio rerio] | 3.44 |  |  |  |  |  |  |
| gi|82079428 | Secernin-2 | 6.51 |  |  |  |  |  |  |
| gi|48994868 | secretogranin II [Ctenopharyngodon idella] | 21.60 | 0.650 |  |  | 0.516 |  |  |
| gi|4001809 | secretogranin II precursor [Carassius auratus] | 6.14 |  |  |  |  |  |  |
| gi|45387537 | secretory carrier membrane protein 5 [Danio rerio] | 9.13 | 2.484 |  |  | 1.824 |  |  |
| gi|41055742 | selenium binding protein 1 [Danio rerio] | 9.19 |  |  |  |  |  |  |
| gi|94574481 | Sept2 protein [Danio rerio] | 10.87 |  |  |  |  |  |  |
| gi|47086783 | septin 6 [Danio rerio] | 13.35 | 1.272 | 0.298 | 1.839 | 1.572 | 0.116 | 1.930 |
| gi|439153 | serine protease inhibitor | 10.73 | 0.746 |  |  | 1.304 | 0.418 | 13.511 |
| gi|58801528 | SET translocation (myeloid leukemia-associated) A [Danio rerio] | 3.72 | 0.785 |  |  | 1.000 |  |  |
| gi|41055363 | SH3-domain GRB2-like endophilin B2 [Danio rerio] | 6.70 |  |  |  |  |  |  |
| gi|509406 | sodium /potassium-transporting ATPase, alpha subunit [Anguilla anguilla] | 43.93 | 2.216 |  |  | 1.725 |  |  |
| gi|49037292 | sodium potassium ATPase alpha subunit [Rhabdosargus sarba] | 41.25 |  |  |  |  |  |  |
| gi|45709332 | Solute carrier family 25 (mitochondrial carrier; phosphate carrier), member 3 [Danio rerio] | 9.83 | 1.045 | 0.886 | 21.925 | 1.081 | 0.335 | 1.778 |
| gi|41107664 | Solute carrier family 25 alpha, member 5 [Danio rerio] | 42.62 | 1.133 | 0.298 | 1.318 | 1.150 | 0.300 | 1.429 |
| gi|148596963 | spectrin alpha 2 [Danio rerio] | 35.65 | 1.189 | 0.134 | 1.265 | 1.314 | 0.032 | 1.277 |
| gi|49619153 | S-phase kinase-associated protein 1A [Danio rerio] | 15.34 |  |  |  |  |  |  |
| gi|41053873 | succinate dehydrogenase complex, subunit A, flavoprotein (Fp) [Danio rerio] | 8.77 | 0.955 |  |  | 1.554 |  |  |
| gi|56790262 | superoxide dismutase 1, soluble [Danio rerio] | 24.68 | 1.122 | 0.649 | 10.847 | 0.797 |  |  |
| gi|55962254 | superoxide dismutase 2, mitochondrial [Danio rerio] | 8.48 |  |  |  |  |  |  |
| gi|29611414 | synapsin 2A [Takifugu rubripes] | 6.86 | 1.123 |  |  | 1.132 |  |  |
| gi|71164796 | Synaptic vesicle membrane protein VAT-1 homolog | 38.22 | 0.979 | 0.757 | 1.158 | 0.928 | 0.338 | 1.177 |
| gi|46362505 | Synaptotagmin binding, cytoplasmic RNA interacting protein, like [Danio rerio] | 4.64 | 1.160 |  |  | 1.081 |  |  |
| gi|68448507 | syntaxin binding protein 1 [Danio rerio] | 28.76 | 0.932 | 0.591 | 1.397 | 1.109 | 0.522 | 1.576 |
| gi|94732621 | syntaxin1b [Danio rerio] | 42.71 | 1.097 | 0.366 | 1.255 | 1.043 | 0.820 | 1.567 |
| gi|53933218 | tetraspanin 7b [Danio rerio] | 6.02 |  |  |  |  |  |  |
| gi|70721616 | TPA_exp: RTN5 [Cyprinus carpio] | 29.63 | 1.080 |  |  | 1.303 |  |  |
| gi|42601270 | TPA_inf: RTN1 [Cyprinus carpio] | 22.17 |  |  |  |  |  |  |
| gi|24571214 | transaldolase [Ctenopharyngodon idella] | 8.11 |  |  |  |  |  |  |
| gi|55742472 | transaldolase 1 [Danio rerio] | 11.28 | 0.946 | 0.062 | 1.072 | 1.125 | 0.308 | 2.200 |
| gi|22087586 | transferrin variant A1 [Carassius auratus] | 35.29 | 1.042 | 0.717 | 1.318 | 1.485 | 0.009 | 1.259 |
| gi|22087589 | transferrin variant B1 [Carassius auratus] | 22.84 | 0.534 | 0.045 | 1.757 | 0.807 |  |  |
| gi|37682085 | transketolase [Danio rerio] | 17.28 | 0.933 |  |  | 0.928 |  |  |
| gi|46849397 | triose phosphate isomerase [Lepisosteus osseus] | 50.00 |  |  |  |  |  |  |
| gi|34221914 | triosephosphate isomerase [Oncorhynchus mykiss] | 48.10 | 1.113 |  |  | 0.735 |  |  |
| gi|24119234 | triosephosphate isomerase 1a [Danio rerio] | 63.71 | 0.950 | 0.865 | 20.258 | 0.904 | 0.807 | 60.790 |
| gi|47271422 | triosephosphate isomerase 1b [Danio rerio] | 70.16 | 0.837 | 0.026 | 1.160 | 0.757 | 0.011 | 1.206 |
| gi|41393141 | tropomyosin 3 [Danio rerio] | 41.94 | 1.049 | 0.876 | 3.217 | 0.762 | 0.279 | 2.218 |
| gi|28557124 | tropomyosin1-2 [Takifugu rubripes] | 42.41 | 0.681 |  |  | 0.720 |  |  |
| gi|55295396 | tryosine 3-monooxygenase/tryptophan 5-monooxygenase activation protein, zeta polypeptide [Danio rerio] | 66.12 | 1.006 | 0.970 | 1.626 | 1.196 | 0.493 | 2.076 |
| gi|64388 | trypsin III [Salmo salar] | 8.82 | 0.731 | 0.137 | 2.393 | 0.997 | 0.982 | 4.268 |
| gi|549053 | Tubulin beta-1 chain (Beta-1 tubulin) | 59.64 | 2.065 |  |  | 2.039 |  |  |
| gi|54261761 | tubulin, alpha 2 [Danio rerio] | 60.31 |  |  |  |  |  |  |
| gi|37362304 | tubulin, alpha 2 [Danio rerio] | 45.66 |  |  |  |  |  |  |
| gi|41152353 | tubulin, alpha 8 like 2 [Danio rerio] | 48.33 | 0.927 | 0.355 | 1.818 | 0.761 |  |  |
| gi|56565281 | type 1 collagen alpha 1 [Paralichthys olivaceus] | 8.91 |  |  |  |  |  |  |
| gi|15028976 | type II keratin E1 [Oncorhynchus mykiss] | 28.80 |  |  |  |  |  |  |
| gi|41152453 | tyrosine 3-monooxygenase/tryptophan 5-monooxygenase activation protein, beta polypeptide like [Danio rerio] | 61.22 | 1.511 | 0.205 | 5.756 | 1.102 |  |  |
| gi|47086819 | tyrosine 3-monooxygenase/tryptophan 5-monooxygenase activation protein, epsilon polypeptide [Danio rerio] | 69.02 | 1.039 | 0.765 | 1.621 | 1.124 | 0.047 | 1.119 |
| gi|61651838 | tyrosine 3-monooxygenase/tryptophan 5-monooxygenase activation protein, epsilon polypeptide 2 [Danio rerio] | 72.55 |  |  |  |  |  |  |
| gi|47085939 | tyrosine 3-monooxygenase/tryptophan 5-monooxygenase activation protein, gamma polypeptide [Danio rerio] | 36.99 | 0.616 |  |  | 0.925 |  |  |
| gi|56207926 | tyrosine 3-monooxygenase\/tryptophan 5-monooxygenase activation protein, theta polypeptide [Danio rerio] | 44.08 | 0.871 | 0.548 | 7.663 | 1.173 |  |  |
| gi|68085024 | Ubiquinol-cytochrome c reductase core protein II [Danio rerio] | 10.35 |  |  |  |  |  |  |
| gi|37595366 | ubiquitin C [Danio rerio] | 94.47 | 0.818 | 0.070 | 1.246 | 0.776 | 0.002 | 1.144 |
| gi|115361552 | ubiquitin C-terminal hydrolase L1 [Carassius auratus] | 7.91 |  |  |  |  |  |  |
| gi|47085781 | ubiquitin-activating enzyme E1 (A1S9T and BN75 temperature sensitivity complementing) [Danio rerio] | 10.78 | 0.983 |  |  | 1.005 |  |  |
| gi|47087259 | ubiquitin-conjugating enzyme E2 variant 2 [Danio rerio] | 47.59 | 1.000 | 0.998 | 1.667 | 1.023 | 0.886 | 1.588 |
| gi|94734326 | ubiquitin-conjugating enzyme E2N [Danio rerio] | 31.17 | 0.784 | 0.204 | 2.787 | 1.071 | 0.801 | 2.781 |
| gi|53733738 | Unknown (protein for IMAGE:7227623) [Danio rerio] | 19.36 | 1.379 | 0.020 | 1.139 | 1.743 | 0.170 | 6.870 |
| gi|120537540 | Unknown (protein for IMAGE:7294692) [Danio rerio] | 36.69 |  |  |  |  |  |  |
| gi|148745148 | Unknown (protein for MGC:165456) [Danio rerio] | 28.57 |  |  |  |  |  |  |
| gi|148745194 | Unknown (protein for MGC:165661) [Danio rerio] | 8.74 |  |  |  |  |  |  |
| gi|47218629 | unnamed protein product [Tetraodon nigroviridis] | 53.19 | 0.895 | 0.109 | 1.148 | 0.962 | 0.566 | 1.149 |
| gi|47207317 | unnamed protein product [Tetraodon nigroviridis] | 39.38 |  |  |  |  |  |  |
| gi|47223560 | unnamed protein product [Tetraodon nigroviridis] | 23.38 | 0.899 | 0.148 | 1.377 | 1.205 | 0.096 | 1.430 |
| gi|47222711 | unnamed protein product [Tetraodon nigroviridis] | 45.15 | 0.647 | 0.001 | 1.210 | 0.735 | 0.076 | 1.416 |
| gi|47227811 | unnamed protein product [Tetraodon nigroviridis] | 51.01 | 0.585 | 0.525 | 1602.874 | 0.624 | 0.585 | 2603.386 |
| gi|47225212 | unnamed protein product [Tetraodon nigroviridis] | 38.03 | 0.878 | 0.042 | 1.133 | 1.036 | 0.714 | 1.232 |
| gi|47211813 | unnamed protein product [Tetraodon nigroviridis] | 13.29 | 1.206 |  |  | 1.368 |  |  |
| gi|47221269 | unnamed protein product [Tetraodon nigroviridis] | 9.47 |  |  |  |  |  |  |
| gi|47207673 | unnamed protein product [Tetraodon nigroviridis] | 12.85 | 1.012 | 0.929 | 3.969 | 1.144 | 0.808 | 240.237 |
| gi|47221390 | unnamed protein product [Tetraodon nigroviridis] | 37.59 | 1.325 | 0.165 | 2.583 | 1.279 | 0.719 | 747.273 |
| gi|47204597 | unnamed protein product [Tetraodon nigroviridis] | 27.13 | 0.674 | 0.029 | 1.387 | 0.613 | 0.017 | 1.414 |
| gi|47219649 | unnamed protein product [Tetraodon nigroviridis] | 14.53 | 1.211 |  |  | 2.381 | 0.215 | 48.169 |
| gi|47224065 | unnamed protein product [Tetraodon nigroviridis] | 46.88 | 0.733 |  |  | 0.978 |  |  |
| gi|47224490 | unnamed protein product [Tetraodon nigroviridis] | 54.61 | 0.826 | 0.265 | 1.562 | 0.854 | 0.164 | 1.314 |
| gi|47210341 | unnamed protein product [Tetraodon nigroviridis] | 23.06 | 0.925 | 0.641 | 4.838 | 0.771 | 0.098 | 1.671 |
| gi|47214851 | unnamed protein product [Tetraodon nigroviridis] | 13.74 | 1.139 | 0.687 | 22.020 | 1.074 | 0.880 | 115.855 |
| gi|47209542 | unnamed protein product [Tetraodon nigroviridis] | 13.89 | 1.596 |  |  | 2.083 |  |  |
| gi|47221743 | unnamed protein product [Tetraodon nigroviridis] | 32.51 | 1.000 | 0.998 | 1.949 | 0.702 | 0.508 | 99.182 |
| gi|47221147 | unnamed protein product [Tetraodon nigroviridis] | 18.35 | 0.829 | 0.382 | 5.102 | 0.718 |  |  |
| gi|47217168 | unnamed protein product [Tetraodon nigroviridis] | 14.55 | 1.475 | 0.475 | 95.930 | 2.020 | 0.358 | 276.290 |
| gi|47215544 | unnamed protein product [Tetraodon nigroviridis] | 27.20 |  |  |  |  |  |  |
| gi|47229309 | unnamed protein product [Tetraodon nigroviridis] | 11.11 | 1.208 |  |  | 1.431 |  |  |
| gi|47217449 | unnamed protein product [Tetraodon nigroviridis] | 18.02 | 0.969 |  |  | 0.722 |  |  |
| gi|47213348 | unnamed protein product [Tetraodon nigroviridis] | 47.77 | 0.715 | 0.048 | 1.392 | 0.692 | 0.170 | 1.844 |
| gi|47212835 | unnamed protein product [Tetraodon nigroviridis] | 17.57 | 1.526 |  |  | 0.865 |  |  |
| gi|47192457 | unnamed protein product [Tetraodon nigroviridis] | 20.49 |  |  |  |  |  |  |
| gi|47224526 | unnamed protein product [Tetraodon nigroviridis] | 17.76 | 0.981 | 0.930 | 8.834 | 1.334 | 0.273 | 5.335 |
| gi|47221746 | unnamed protein product [Tetraodon nigroviridis] | 10.22 | 1.193 | 0.217 | 2.220 | 1.110 | 0.308 | 2.003 |
| gi|47210455 | unnamed protein product [Tetraodon nigroviridis] | 16.73 | 0.978 |  |  | 1.036 |  |  |
| gi|47225149 | unnamed protein product [Tetraodon nigroviridis] | 7.39 | 0.673 |  |  | 0.544 |  |  |
| gi|47220653 | unnamed protein product [Tetraodon nigroviridis] | 7.51 |  |  |  |  |  |  |
| gi|47211130 | unnamed protein product [Tetraodon nigroviridis] | 28.26 |  |  |  |  |  |  |
| gi|47219625 | unnamed protein product [Tetraodon nigroviridis] | 11.71 |  |  |  |  |  |  |
| gi|47228547 | unnamed protein product [Tetraodon nigroviridis] | 27.64 |  |  |  |  |  |  |
| gi|47224458 | unnamed protein product [Tetraodon nigroviridis] | 7.21 |  |  |  |  |  |  |
| gi|47227232 | unnamed protein product [Tetraodon nigroviridis] | 13.71 |  |  |  |  |  |  |
| gi|47217896 | unnamed protein product [Tetraodon nigroviridis] | 5.73 | 2.044 |  |  | 2.048 |  |  |
| gi|47211930 | unnamed protein product [Tetraodon nigroviridis] | 11.37 |  |  |  |  |  |  |
| gi|47205851 | unnamed protein product [Tetraodon nigroviridis] | 11.40 |  |  |  |  |  |  |
| gi|47216787 | unnamed protein product [Tetraodon nigroviridis] | 5.41 |  |  |  |  |  |  |
| gi|47212048 | unnamed protein product [Tetraodon nigroviridis] | 11.11 | 1.197 | 0.493 | 9.329 | 1.233 | 0.097 | 1.505 |
| gi|47227171 | unnamed protein product [Tetraodon nigroviridis] | 17.12 | 0.927 |  |  | 0.924 |  |  |
| gi|47205849 | unnamed protein product [Tetraodon nigroviridis] | 34.15 |  |  |  |  |  |  |
| gi|47210017 | unnamed protein product [Tetraodon nigroviridis] | 63.28 |  |  |  |  |  |  |
| gi|47223004 | unnamed protein product [Tetraodon nigroviridis] | 10.80 |  |  |  |  |  |  |
| gi|47212654 | unnamed protein product [Tetraodon nigroviridis] | 13.83 |  |  |  |  |  |  |
| gi|47218711 | unnamed protein product [Tetraodon nigroviridis] | 22.06 | 1.120 | 0.051 | 1.122 | 1.218 | 0.398 | 6.090 |
| gi|47221580 | unnamed protein product [Tetraodon nigroviridis] | 24.32 |  |  |  |  |  |  |
| gi|47208134 | unnamed protein product [Tetraodon nigroviridis] | 3.28 |  |  |  |  |  |  |
| gi|47215937 | unnamed protein product [Tetraodon nigroviridis] | 16.83 |  |  |  |  |  |  |
| gi|47219626 | unnamed protein product [Tetraodon nigroviridis] | 21.51 |  |  |  |  |  |  |
| gi|47225748 | unnamed protein product [Tetraodon nigroviridis] | 5.05 |  |  |  |  |  |  |
| gi|47229302 | unnamed protein product [Tetraodon nigroviridis] | 10.92 | 0.224 |  |  |  |  |  |
| gi|47224253 | unnamed protein product [Tetraodon nigroviridis] | 13.64 | 0.856 |  |  |  |  |  |
| gi|47224676 | unnamed protein product [Tetraodon nigroviridis] | 27.96 |  |  |  |  |  |  |
| gi|47217066 | unnamed protein product [Tetraodon nigroviridis] | 9.47 | 1.352 |  |  | 1.484 |  |  |
| gi|47213473 | unnamed protein product [Tetraodon nigroviridis] | 18.97 |  |  |  |  |  |  |
| gi|47225287 | unnamed protein product [Tetraodon nigroviridis] | 13.94 |  |  |  | 0.894 |  |  |
| gi|47220725 | unnamed protein product [Tetraodon nigroviridis] | 4.00 |  |  |  |  |  |  |
| gi|47218064 | unnamed protein product [Tetraodon nigroviridis] | 6.51 | 0.860 |  |  | 1.146 |  |  |
| gi|47222914 | unnamed protein product [Tetraodon nigroviridis] | 3.17 |  |  |  |  |  |  |
| gi|47222109 | unnamed protein product [Tetraodon nigroviridis] | 5.73 |  |  |  |  |  |  |
| gi|47200404 | unnamed protein product [Tetraodon nigroviridis] | 9.74 |  |  |  |  |  |  |
| gi|47193705 | unnamed protein product [Tetraodon nigroviridis] | 23.16 |  |  |  |  |  |  |
| gi|47224562 | unnamed protein product [Tetraodon nigroviridis] | 6.16 |  |  |  |  |  |  |
| gi|47219257 | unnamed protein product [Tetraodon nigroviridis] | 5.95 |  |  |  |  |  |  |
| gi|47213751 | unnamed protein product [Tetraodon nigroviridis] | 7.52 |  |  |  |  |  |  |
| gi|47213201 | unnamed protein product [Tetraodon nigroviridis] | 6.89 |  |  |  |  |  |  |
| gi|47209168 | unnamed protein product [Tetraodon nigroviridis] | 8.91 |  |  |  |  |  |  |
| gi|47229899 | unnamed protein product [Tetraodon nigroviridis] | 33.82 | 0.749 |  |  | 0.705 |  |  |
| gi|47223834 | unnamed protein product [Tetraodon nigroviridis] | 14.29 |  |  |  |  |  |  |
| gi|47211870 | unnamed protein product [Tetraodon nigroviridis] | 19.20 |  |  |  |  |  |  |
| gi|47211107 | unnamed protein product [Tetraodon nigroviridis] | 8.57 |  |  |  |  |  |  |
| gi|47227310 | unnamed protein product [Tetraodon nigroviridis] | 8.77 | 1.765 |  |  |  |  |  |
| gi|47209375 | unnamed protein product [Tetraodon nigroviridis] | 6.71 |  |  |  |  |  |  |
| gi|47215719 | unnamed protein product [Tetraodon nigroviridis] | 10.46 |  |  |  |  |  |  |
| gi|47224733 | unnamed protein product [Tetraodon nigroviridis] | 10.13 |  |  |  |  |  |  |
| gi|47223368 | unnamed protein product [Tetraodon nigroviridis] | 32.23 | 0.991 |  |  |  |  |  |
| gi|47197545 | unnamed protein product [Tetraodon nigroviridis] | 8.17 |  |  |  |  |  |  |
| gi|47221356 | unnamed protein product [Tetraodon nigroviridis] | 2.95 |  |  |  |  |  |  |
| gi|47227133 | unnamed protein product [Tetraodon nigroviridis] | 48.53 |  |  |  |  |  |  |
| gi|47214338 | unnamed protein product [Tetraodon nigroviridis] | 13.40 | 0.958 |  |  | 1.025 |  |  |
| gi|47223161 | unnamed protein product [Tetraodon nigroviridis] | 7.18 |  |  |  |  |  |  |
| gi|47209953 | unnamed protein product [Tetraodon nigroviridis] | 21.51 |  |  |  |  |  |  |
| gi|47220623 | unnamed protein product [Tetraodon nigroviridis] | 3.07 |  |  |  |  |  |  |
| gi|47223123 | unnamed protein product [Tetraodon nigroviridis] | 6.48 |  |  |  |  |  |  |
| gi|47222263 | unnamed protein product [Tetraodon nigroviridis] | 10.64 |  |  |  |  |  |  |
| gi|47220951 | unnamed protein product [Tetraodon nigroviridis] | 16.75 |  |  |  |  |  |  |
| gi|47214695 | unnamed protein product [Tetraodon nigroviridis] | 12.86 |  |  |  |  |  |  |
| gi|82188219 | Vacuolar ATP synthase subunit C 1-A (V-ATPase subunit C 1-A) (Vacuolar proton pump subunit C 1-A) | 21.15 | 1.355 | 0.414 | 3.589 | 1.428 | 0.313 | 3.146 |
| gi|41152466 | vacuolar H+ ATPase G1 [Danio rerio] | 43.22 | 0.803 | 0.526 | 3.464 | 0.741 | 0.326 | 2.722 |
| gi|5881241 | vacuolar-type H+ transporting ATPase B1 subunit [Anguilla anguilla] | 38.17 |  |  |  |  |  |  |
| gi|51949893 | valosin containing protein [Oncorhynchus mykiss] | 31.02 | 0.914 | 0.495 | 1.370 | 0.934 | 0.660 | 1.459 |
| gi|50540158 | VAMP (vesicle-associated membrane protein)-associated protein A, like [Danio rerio] | 9.35 | 1.435 |  |  | 1.589 |  |  |
| gi|49618999 | v-ATPase AC39 subunit [Danio rerio] | 3.14 |  |  |  |  |  |  |
| gi|84043154 | vesicle-associated membrane protein 2 [Scophthalmus maximus] | 56.88 |  |  |  |  |  |  |
| gi|57526694 | vesicle-associated membrane protein 3 [Danio rerio] | 33.61 |  |  |  |  |  |  |
| gi|47086745 | vesicle-associated membrane protein, associated protein B and C [Danio rerio] | 12.76 |  |  |  |  |  |  |
| gi|9457229 | vimentin [Cyprinus carpio] | 24.62 |  |  |  |  |  |  |
| gi|388286 | vimentin beta | 17.56 |  |  |  |  |  |  |
| gi|68165945 | voltage-dependent anion channel 2 [Danio rerio] | 33.92 | 0.958 | 0.778 | 1.772 | 0.821 | 0.460 | 2.542 |
| gi|801738 | warm temperature acclimation-related 65-kDa protein [Carassius auratus] | 8.99 | 0.484 | 0.058 | 2.331 | 0.614 | 0.401 | 91.573 |
| gi|47938859 | Ywhai protein [Danio rerio] | 54.51 | 0.880 | 0.498 | 5.011 | 0.870 | 0.717 | 41.182 |
| gi|63102133 | Zgc:109976 [Danio rerio] | 38.28 | 0.778 | 0.240 | 1.728 | 0.891 | 0.247 | 1.291 |
| gi|63100518 | Zgc:112425 [Danio rerio] | 44.86 | 1.005 | 0.996 | 5973.886 | 0.578 |  |  |
| gi|90112045 | Zgc:136710 protein [Danio rerio] | 25.62 | 0.683 | 0.003 | 1.226 | 0.533 | 0.000 | 1.212 |
| gi|94574493 | Zgc:136766 [Danio rerio] | 19.32 | 1.644 | 0.414 | 122.358 | 1.141 | 0.185 | 1.648 |
| gi|116284226 | Zgc:153700 protein [Danio rerio] | 29.69 |  |  |  |  |  |  |
| gi|94574333 | Zgc:153867 protein [Danio rerio] | 14.29 |  |  |  |  |  |  |
| gi|41946787 | Zgc:55876 protein [Danio rerio] | 24.55 |  |  |  |  |  |  |
| gi|41944583 | Zgc:65851 [Danio rerio] | 21.00 | 0.787 |  |  | 0.589 |  |  |
| gi|42542734 | Zgc:65861 [Danio rerio] | 9.55 | 1.183 |  |  | 0.674 |  |  |
| gi|47938873 | Zgc:65894 protein [Danio rerio] | 60.14 |  |  |  |  |  |  |
| gi|49902942 | Zgc:92704 [Danio rerio] | 65.67 | 1.193 | 0.386 | 1.994 | 1.257 | 0.205 | 1.703 |
|  |  |  |  |  |  |  |  |  |
